# Characterization of HTLV-1 Infectious Molecular Clone Isolated from Patient with HAM/TSP and Immortalization of Human Primary T-Cell Lines

**DOI:** 10.3390/v16111755

**Published:** 2024-11-09

**Authors:** Marcia Bellon, Pooja Jain, Christophe Nicot

**Affiliations:** 1 Department of Pathology and Laboratory Medicine, University of Kansas Medical Center, 3901 Rainbow Blvd, Kansas City, KS 66160, USA; mbellon@kumc.edu; 2 Department of Microbiology & Immunology, Drexel University College of Medicine, Philadelphia, PA 19129, USA; pj27@drexel.edu

**Keywords:** HTLV-1, HAM/TSP, ATL, immortalization, dendritic cells, Tax, HBZ

## Abstract

Human T-cell leukemia virus (HTLV-1) is the etiological agent of lymphoproliferative diseases such as adult T-cell leukemia and T-cell lymphoma (ATL) and a neurodegenerative disease known as HTLV-1-associated myelopathy/tropical spastic paraparesis (HAM/TSP). While several molecular clones of HTLV-1 have been published, all were isolated from samples derived from patients with adult T-cell leukemia. Here, we report the characterization of an HTLV-1 infectious molecular clone isolated from a sample of a patient with HAM/TSP disease. Genetic comparative analyses of the HAM/TSP molecular clone (pBST) revealed unique genetic alterations and specific viral mRNA expression patterns. Interestingly, our clone also harbors characteristics previously published to favor the development of HAM/TSP disease. The molecular clone is capable of infection and immortalization of human primary T cells in vitro. Our studies further demonstrate that the HTLV-1 virus produced from primary T cells transfected with pBST or ACH molecular clones cannot sustain long-term expansion, and cells cease to proliferate after 3–4 months in culture. In contrast, long-term proliferation and immortalization were achieved if the virus was transmitted from dendritic cells to primary T cells, and secondary infection of 729B cells in vitro was demonstrated. In both primary T cells and 729B cells, pBST and ACH were latent, and only *hbz* viral RNA was detected. This study suggests that HTLV-1 transmission from DC to T cells favors the immortalization of latently infected cells.

## 1. Introduction

Human T-cell leukemia virus (HTLV-1) is the only human retrovirus associated with adult T-cell leukemia/lymphoma (ATL) and with a progressive neurodegenerative disease known as tropical spastic paraparesis/HTLV-associated myelopathy (HAM/TSP) [1,2,3,4,5]. HTLV-1 infection is also associated with the development of HTLV-1-Associated Arthropathy (HAAP), HTLV-1-Associated Uveitis (HAU), infective dermatitis, and polymyositis [6].

Epidemiological studies show that more than 20 million individuals are infected with HTLV-1 worldwide; however, since many infected individuals are asymptomatic, this number is believed to be underestimated [7,8,9]. Unlike most tropical diseases, which depend on specific local environmental factors, sexually transmitted diseases can spread beyond isolated populations in a globalized world with large migrations. The recent increase in population migrations is likely to intensify in the light of future challenges associated with global climate changes [10,11,12,13]. Since HTLV-1 endemic areas overlap low-income countries, which are also the immigrating populations, the HTLV-1 infection rate in high-income countries will likely increase in the next decade. Furthermore, HTLV-1-associated diseases develop more than 20 years after initial infection. Since the initial infection by HTLV-1 is not associated with specific symptoms, most individuals do not know they have become infected, unknowingly spreading the virus for years [8,14,15]. The lifetime cumulative risk of developing an HTLV-1-associated disease is generally around 5% but may reach 20% or more in patients with ATL who have high proviral loads [16,17]. The broad distribution of the HTLV-1 receptor/coreceptor, Glut1, and Neuropilin results in a broad cellular tropism including dendritic cells, macrophages, and B-, CD4+-, and CD8+-T cells [18,19]. However, CD4 T cells frequently undergo clonal expansion following infection by HTLV-1 [20,21]. In vitro, infected T-cells usually expand through an initial phase in which cells remain strictly dependent on exogenous IL-2, referred to as immortalized. Rarely, after a prolonged period of culture in vitro, a selected clonal or oligo-clonal cell population may become independent of IL-2, referred to as transformed. The viral oncoprotein Tax is a potent transcriptional activator of the viral LTR and virus gene expression [22,23]. Tax protein is sufficient to immortalize human primary lymphocytes and plays a critical role in the T-cell transformation process [24]. Tax has been shown to activate cellular signaling pathways, inactivate cell cycle checkpoints, inhibit tumor suppressors, and indirectly activate inhibitors of apoptosis (upregulation of Bcl-xL and MCL1). In addition, Tax impairs DNA replication, creates an unstable genome, and inhibits DNA repair pathways, promoting the accumulation of genetic mutations [25,26]. Tax-mediated NF-κB activation results in cytokine receptor/cytokine autocrine loop and c-Myc-dependent reactivation of human telomerase (hTERT) expression, leading to uncontrolled cellular proliferation and an expanded lifespan of pre-tumoral cells [27,28]. Once transformed, infected cells appear to rely on both Tax and HBZ expression for survival and proliferation [29,30,31].

The clinical outcome of HTLV-1 infection is poorly understood, and the reasons why patients with infection develop leukemia/lymphoma or neurological diseases remain to be fully elucidated. Familial cases of ATL suggest that genetic predisposition factors exist [32,33]. In a large cohort of several hundred patients infected with HTLV-1, methylation of the Fragile Histidine Triad Diadenosine Triphosphatase (FHIT) gene promoter was shown to represent a predisposition factor to ATL disease [34]. Other studies have demonstrated a group of driver mutations (PLCG1, PRKCB, CCR4, TP53, and NOTCH1) associated with progression from asymptomatic carriers (AC) to ATL [35]. The risk of developing HAM/TSP has also been associated with an excessive host immune response resulting in chronic inflammation and tissue damage as well as infection with different HTLV-1 subtypes [36]. The risk of HAM/TSP increases exponentially once the HTLV-1 PVL (proviral load) exceeds 1% of PBMCs, and PVL is a risk factor for disease progression [37]. Consequently, tight regulation of virus expression and the establishment of latency are defining factors. Additional factors have been proposed to play an important role in the development of HAM/TSP disease, including host HLA polymorphism and the genetic variation of HTLV-1 subtypes [36].

While specific differences in HTLV-1 proviruses from ATL or patients with HAM/TSP have not been identified, this does not mean such alterations do not exist. For instance, it is possible that a combination of genetic alterations rather than a single change would be involved in triggering distinct pathogeneses. Viral differences that may account for the risk of ATL or HAM/TSP include higher provirus expression, which is dictated by LTR optimal sequence, Tax functions such as LTR transactivation and NF-kB stimulation, and expression of negative regulators such as Rex, HBZ, and p30. In addition, some studies have shown that HBZ subcellular localization is different in ATL versus HAM/TSP [38]. Specific alterations in p12 have also been reported to be more prevalent in patients with HAM/TSP [39].

Here, we report the first characterization of a HAM/TSP-derived HTLV-1 infectious molecular clone. Our studies identified unique characteristics and specific modifications that have been previously linked to HAM/TSP pathogenesis. This molecular clone provides a unique tool for genetic manipulation and in vivo study of HTLV-1-associated HAM/TSP pathogenesis.

## 2. Materials and Methods

Expression plasmids. HTLV-1 full-length provirus bacteriophage λ DNA P4.39 [40] was digested with the BstEII restriction enzyme, and the cohesive ends were blunted with the Klenow fragment of the DNA polymerase I for 10 min at room temperature. The gel-eluted DNA fragment was then cloned into pBR327, previously digested with EcoR1 and Sal1, blunted with Klenow, and dephosphorylated using Calf Intestinal (CIP) Alkaline Phosphatase for 30 min at 37°C. The ligated products were transformed into DH5a-competent cells, and miniprep DNA was screened using Sac1 digestion to identify a full-length pBST molecular clone. Infectious molecular clone ACH (gift from Dr. L. Ratner) has been previously described [41]. We constructed the HTLV-1 LTR-Luciferase vector by PCR amplification of the full-length LTR derived from the pBST and cloned it into the pGL3-basic vector. The HTLV-1 envelope gene was PCR amplified from pBST plasmid DNA, cloned using the TA cloning kit, and transferred to the pCDNA3.1 expression vector.

Cell lines and primary cells. Human B cell line 729B (gift from Dr. Lee Ratner) was cultured in RPMI1640 supplemented with 10% fetal calf serum (FBS) and pen/strep antibiotics. Human primary peripheral blood mononuclear cells (PBMCs) were purchased from StemCell, activated with PHA for 72 h, washed, and cultured with RPMI 1640 supplemented with 20% FBS, pen/strep, and 50 U/mL of interleukin 2 (IL-2). Human CD4+ T cells were isolated with magnetic beads (Dynabeads) according to the manufacturer’s instructions. Isolation of dendritic cells from peripheral blood was performed by the adherence method. Briefly, PBMCs were plated in 20% FBS-containing media for 8 h. Suspension cells were removed from the adhering monocytes by several PBS washes. Adherent dendritic cells were then stimulated by the addition of LPS for 24 h. 293T cells were transfected by calcium phosphate with plasmids expressing human hIL-4 and hGM-CSF (Addgene #74169 and #74168). Dendritic cells were then cultivated in filtered 293T cell-conditioned media containing the IL-4 and GM-CSF for 72 h before HTLV-1 cell-mediated infection. T cells were stimulated with PHA for 48 h, washed, and cultured with IL-2-containing media for 72 h before nucleofector transfection (Amaxa) or cell-mediated infection. Human epithelial cell lines 293T and HeLa (ATCC) were cultured in DMEM high glucose supplemented with 10% FBS and pen/strep. HTLV-1 reporter cell line BHK1E6 harboring an HTLV-1-LTR-beta-galactosidase reporter gene has been previously described [42].

Nucleic acids and clonality analyses. DNA and RNA extractions were carried out by lysing cells in DNAzol and TRIzol, respectively. Messenger RNAs were treated with DNase I to remove any DNA contamination. The reverse transcription was performed using high-capacity cDNA reverse transcription kits (Applied Biosystems, Waltham, MI, USA) according to the manufacturer’s instructions. Reverse transcription polymerase chain reaction (RT-PCR) was performed to detect the expression of viral mRNAs. The primers used for viral mRNA are reported in Table 1.

Luciferase and Syncytia assays. For luciferase assays, 293T cells were transfected with the HTLV-1-LTR-Luciferase construct along with increasing amounts of pBST molecular clones by Polyfect (Qiagen, Hilden, Germany). Then, 48 h later, cells were lysed in a passive lysis buffer and assayed using the Dual Luciferase-Reporter kit (Promega, Madison, WI, USA). Fold change was determined compared to control (empty vector) transfected cells. For syncytia assays, HeLa cells were transfected with pCDNA 3.1 or Env1 pCDNA expression vectors using Polyfect Reagent, as described by the manufacturer. After 24 h, cells were trypsinized and mixed at different ratios with non-transfected Hela cells and plated for an additional 48 h. Cells were fixed for 5 min at room temperature in 1× PBS with 1% paraformaldehyde and 0.1% glutaraldehyde, washed with PBS, and stained with Crystal Violet. Syncytia were scored for cells with 4 or more individualized nuclei.

Detection of cell-mediated infections. The previously characterized HTLV-1 reporter cell line BHK1E6 was used for cell-mediated infections [42]. The cell line carries an HTLV-1 LTR fused to the beta-galactosidase gene. Following infection, newly expressed Tax protein transactivates the LTR and releases the expression of beta-galactosidase. Newly, infected cells can then be detected by a blue color following staining with X-Gal. Briefly, co-cultivation infection assays were conducted for 48 h with 729B cells transfected by nucleofactor with pBST. Cells were cultured for an additional 24 h, washed and fixed for 5 min at room temperature, and stained for 6 h with X-gal solution.

Phylogenetic Analysis: Phylogenetic trees were obtained using the SeaView interface, version 4 [43]. The phylogenetic tree was generated with the maximum likelihood (PhyML) method on a 710-nt-long fragment of the LTR. The numbers at the nodes correspond to the bootstrap value, obtained after 1000 repeats. The branch lengths are drawn to scale. HTLV-1c strains were used as an outgroup. Phylogenetic tree generated by the maximum likelihood method (PhyML) on 6366 nt corresponding to the concatenation of gag-pol-env-tax ORFs. The numbers at the nodes correspond to the bootstrap value, obtained after 1000 repeats. The branch lengths are drawn to scale. HTLV-1c strains were used as an outgroup.

Protein Alignments. Amino acid sequence alignment of viral ORFs from the pBST clone was compared to full-length genome sequences from Japanese HAM/TSP (n = 12) or Brazilian HAM/TSP (n = 10). The list of identifiers from Genebank (with accession number) is as follows: Japan HAM/TSP isolates HAM1 LC192536.1; HAM2 LC192535.1; HAM3 LC192534.1, HAM4 LC192533.1, HAM5 LC192532.1; HAM6 LC192531.1; HAM7 LC192530.1; HAM8 LC192529.1; HAM9 LC192528.1; HAM10 LC192527.1; HAM11 LC192526.1; HAM12 LC192525.1. Brazil HAM/TSP isolates HAM1 KY007274.1; HAM2 KY007272.1; HAM3 KY007270.1; HAM4 KY007268.1; HAM5 KY007267.1; HAM6 KY007264.1; HAM7 KY007263.1; HAM8 KY007259.1; HAM9 KY007265.1; HAM10 KY007266.1. For Supplemental S12 HBZ: HAM13 MW288054.1; HAM14 MW288053.1; HAM15 MW288052.1; HAM16 MW288051.1; HAM17 MW288050.1, HAM18 MW288049.1; HAM 19 MW288048.1, HAM20 MW288046.1; HAM21 MW288045.1; HAM22 MW288044.1; HAM23 MW288043.1; HAM24 LC192517.1. Japan ATL isolates #1–17 data from PMID: 16407133. ATL18 LC842178.1, ATL19 LC183874.1, ATL20 J02029.1

Electron microscopy. Cells were transfected with molecular clones ACH or pBST. After 48 h, cell monolayers were fixed on coverslips with 2% glutaraldehyde in 0.1 M sodium cacodylate pH 7.4, post-fixed in reduced 1% osmium tetroxide, dehydrated in a graded series of ethanol, embedded in Embed 812 resin, and polymerized. Blocks were trimmed and sectioned, and ultrathin sections were placed on 250 mesh copper grids, contrasted with 3% uranyl acetate and lead citrate, then viewed using a JEOL JEM-1400 TEM at 100 KV. Digital images were acquired with an AMT digital camera magnification scale bar indicated in the figures.

## 3. Results

### 3.1. Cloning of a Full-Length HAM/TSP Molecular Clone pBST

**Figure 1:**
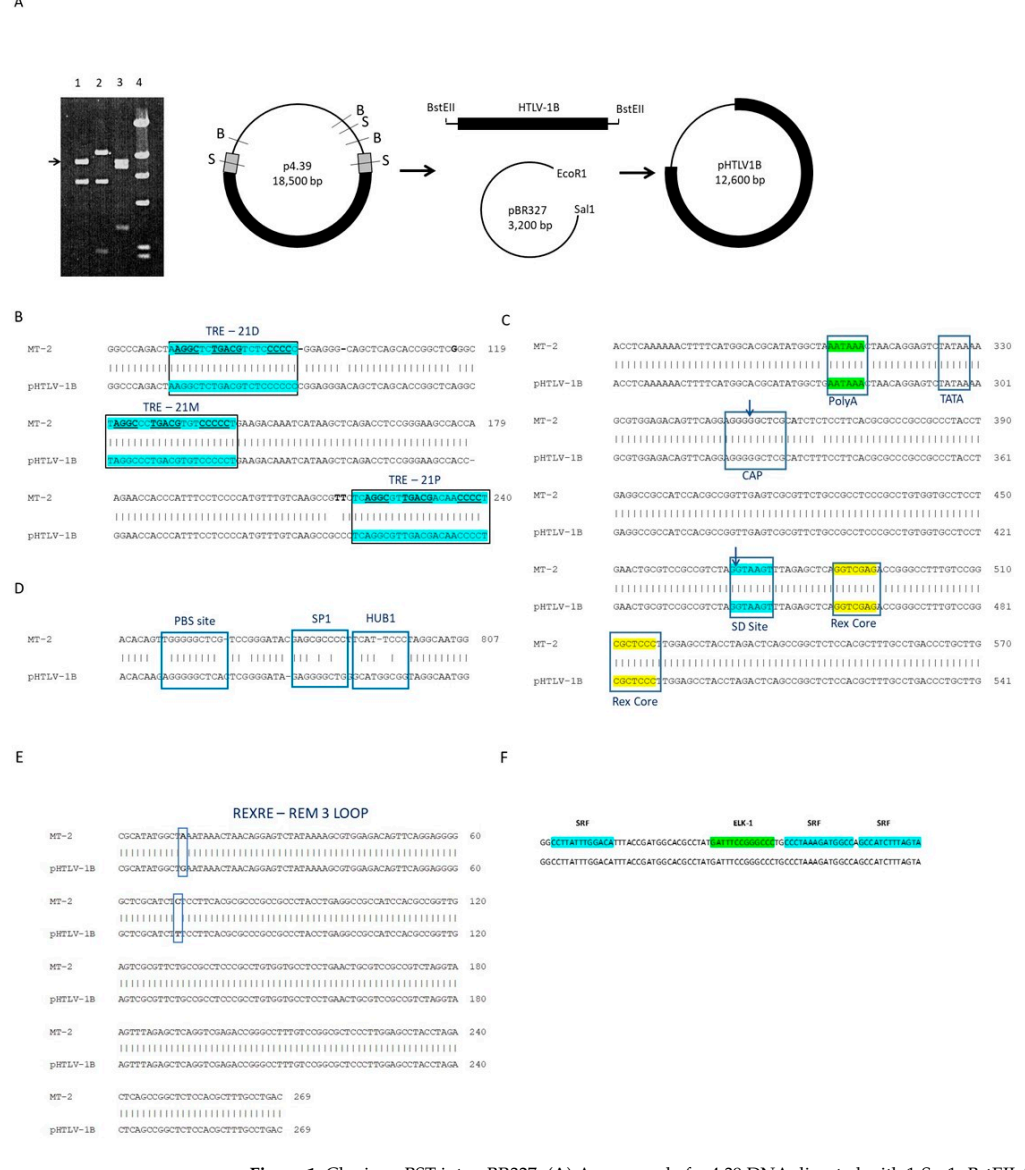
Cloning pBST into pBR327. (**A**) Agarose gel of p.4.39 DNA digested with 1-Sac1+BstEII. 2- BstEII. 3- Sac1. 4- Lambda HindIII DNA marker. Schematic representation of p4.39 with restriction sites for Sac1 (S) or BstEII (B) and pBR327 digested with EcoR1 and Sal1 for cloning. (**B**–**D**) Alignment between pBST and MT-2 sequences of different regions of the viral LTR. (**E**) Alignment between pBST and MT-2 sequence of RexRE REM3 loop. (**F**) The enhancer region conserved sites for transcription factors SRF and ELK-1 in HTLV-1 ACH (**top**) and pBST sequence (**bottom**).

Previously, an HTLV-1 molecular clone was isolated from a patient from French Guiana diagnosed with neurological HAM/TSP [40]. In that study, the CD4+ T-cell line, named 2060, was derived by the cultivation of the patient’s PBMC with heterologous activated PBMCs in vitro. A bacteriophage Lambda genomic library was then constructed with EcoR1 digested high molecular genomic DNA since this enzyme does not cut within the HTLV-1 sequence. The library was screened with two independent DNA probes for *tax* and *gag* genes of HTLV-1. A bacteriophage clone positive for both probes was amplified to isolate a DNA fragment of approximately 16 Kb encompassing the complete HTLV-1 provirus. This fragment was then cloned into a low-copy plasmid, pBR327, and named p4.39 [40]. The large size of this molecular clone, approximately 19.2 Kb, hampered most in vitro studies by making it difficult to amplify in bacteria and significantly reducing transient transfection efficiencies. Since the HTLV-1 provirus size is around 8.5 Kb this suggested that p4.39 had around 7.5 kb of human genomic flanking sequences. In this study, to reduce the size of genomic flanking sequences without altering the HTLV-1 provirus, we selected a series of restriction enzymes that do not cut within the HTLV-1 genome. As expected, digestion with the Sac1 restriction enzyme, which cuts once in the LTR sequence, confirmed the presence of a full-length HTLV-1 provirus: a Sac1 fragment of 8.5 Kb (Figure 1A, lane 3). Interestingly, restriction enzyme BstEII digestion releases a fragment of 10.5 Kb (Figure 1A, lane 2). Since double digestion with Sac1 and BstEII still produced a fragment of 8.5 Kb (Figure 1A, lane 1), these results suggested the presence of three restriction sites for BstEII within the genomic flanking sequences. The fact that Sac1 and BstEII double digestion fragments present on the gel total about 14.4 Kb, but total fragments from the same plasmid digested with either BstEII or Sac1 alone total 18.5 kb, suggested that Sac1 cut between two BstEII sites, producing the Sac1-BstEII smaller fragments, which are not apparent on the gel (Figure 1A, Lane 1). Based on these data, p4.39 was digested with BstEII, cohesive ends were blunted, and the DNA was ligated with T4 DNA ligase. After the transformation of DH5a competent cells, the pBST molecular clone was isolated (Figure 1A). Sequencing of the 5’ flanking cellular sequence revealed the integration of the provirus into the human interleukin nine receptor (IL-9R) gene 5’UTR sequence (Genebank U48256.1). Surprisingly, the same sequence was previously identified in front of the HTLV-1 5’LTR in the HUT102 clone KT1 (Genebank M32443.1).

### 3.2. Analysis of the pBST Provirus mRNA Splicing and Regulatory Signals

A number of HTLV-1, HTLV-2, and HTLV-3 molecular clones have been generated. The HTLV-2 molecular clone, pH6Neo, was derived from an HTLV-2 subtype A isolate, cloned from cDNA isolated from CEM cells infected by co-culture with the HTLV-2 infected Mo cell line [44]. In addition, an HTLV-3 molecular clone has also been characterized, the SV2_Pyl43 cl9_, derived from HTLV-3_Pyl43_, a Bakola pygmy in Southern Cameroon [45]. Although several HTLV-1A subtype molecular clones from patients with ATL have been previously characterized (ACH, pCS-HTLV, K30p, and pCS-X1MT) [46,47,48], no infectious molecular clone derived from HAM/TSP disease has been characterized. The ACH molecular clone was derived from a probable Caribbean patient with ATL, pHTLV-1_CH_ strain, flanked by LTR sequences derived from Japanese HTLV-1_ATK_ at the 5’ end and the Zairian HTLV-1_EL_ LTR at the 3’ end [46,49]. The ATK, HTLV-1 variant is from the Cosmopolitan Japanese Subgroup B. The pCS-HTLV molecular clone was constructed from the HTLV-1 provirus derived from CS-1 cells, obtained by co-cultivating cord blood lymphocytes with HTLV-I-infected cells from an American patient with ATL [47]. The pCS-HTLV variant is from the Cosmopolitan Transcontinental Subgroup A, like the HTLV-1 variant in MT-2 cells. The K30p molecular clone is derived from the rabbit HTLV-I cell line RH/K30, obtained by co-culturing rabbit primary lymphocytes with human MT-2 cells [50]. The pCS-X1MT was derived from the pCS-HTLV molecular clone, where the ORFs X-I and X-II, were replaced with ORFs X-I and X-II from MT-2 cells [51]. Therefore, pBST represents the first and only molecular clone derived from a patient with HAM/TSP.

To better understand pBST characteristics, we sequenced the entire molecular clone (Supplemental S1). HTLV-1 provirus transcription is determined both by basal activity, which varies greatly among cell types, and Tax-mediated transactivation through the three Tax-response elements (TRE) present in the long terminal repeats (LTR) [52,53,54]. Analyses suggest that the TRE motifs are 100% conserved in the pBST molecular clone when compared to the HTLV-1A, MT-2 sequence (GenBank: AB273635.1.) (Figure 1B). In addition, the TATA box, the CAP site, the polyA signal, the PBS site, the Rex core sites, and the Rex RNA responsive element (RexRE) were all conserved in the pBST molecular clone (Figure 1C,D). 

As a retrovirus, HTLV-1 must express unspliced, incompletely spliced, and fully spliced mRNAs. To produce the diverse set of alternatively spliced viral mRNAs, HTLV-1 has to bypass the cellular RNA-processing machinery, which normally removes intron-containing transcripts via splicing and degradation. This is accomplished by positive and negative cis-acting regulatory elements and the viral Rex protein, which binds to the RexRE, located at the 3′ end of viral transcripts [55,56,57]. Alignment analyses demonstrate two nucleotide substitutions in the RexRE REM 3 loop and six changes in the HTLV-1 CRS (cis-Acting Regulatory Sequence) (Figure 1E). However, these genetic alterations do not affect the predicted secondary structures of these regulatory elements. 

We then analyzed the Env splice junction because studies have shown that this region, when fused to the LTR splice donor, encompasses the p30RE and is essential for p30-mediated nuclear retention of *tax/rex* mRNA and suppression of virus replication [58]. No genetic alterations were found in the 195 nucleotides forming that region or the LTR splice donor region (Supplemental S1). Importantly, all known splice donor and splice acceptor site sequences for viral mRNAs were also conserved in pBST (Supplemental S1). Surprisingly, a region with significant divergence was found at the end of the LTR U5 region (Figure 1D). The significance is unclear, but the sequence overlaps with one of the three SP1 binding motifs. Studies have shown that the U5 repressive element (U5RE) contains Sp1 and HTLV-1 U5RE binding protein 1 (HUB1) binding sites at the end of U5 and represses viral LTR-mediated expression of sense mRNAs [59]. Therefore, this region may affect either basal or Tax-mediated activation of the viral LTR. In addition, Sp1 binding sites located at the U5 region are important for the antisense promoter activity and HBZ expression; therefore, alterations in this region may affect *hbz* mRNA expression [60]. This variable region also encompasses the PBS site (TGGGGGCTCG), but alterations do not significantly affect the secondary structure. Overall, we do not think these genetic alterations have a significant effect on virus replication because the pAB molecular clone, which was constructed as a hybrid between the ACH and pBST molecular clones (by substitution of the Sla1-Cla1 fragment of pBST with that of ACH) (Supplemental S2) remains infectious [61]. Since pAB is infectious in vitro and in vivo animal models, these data suggest that the alterations observed in the U5 region do not affect virus replication and infectivity [61]. Finally, we analyzed the previously reported HTLV-1 enhancer encompassing SRF and ELK-1 transcription sites and found no changes when compared to that of the ACH sequence (Figure 1E).

### 3.3. Genetic Characteristics of the pBST Structural and Enzymatic Genes

**Figure 2:**
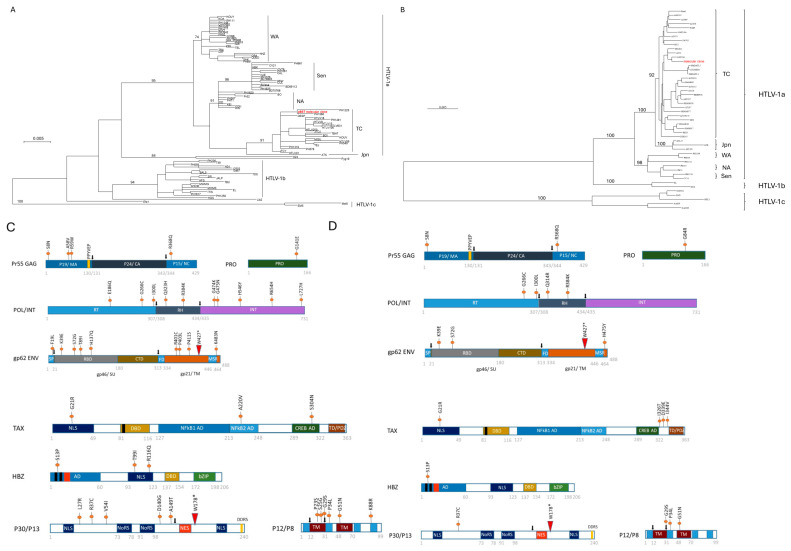
Schematic representation of pBST mutations present in viral proteins. (**A**) Phylogenetic tree analysis, generated with the maximum likelihood (PhyML) method on a 710-nt-long fragment of the LTR. The numbers at the nodes correspond to the bootstrap value, obtained after 1000 repeats. The branch lengths are drawn to scale. HTLV-1c strains were used as outgroup. (**B**) Phylogenetic tree generated by the maximum likelihood method (PhyML) on 6366 nt corresponding to the concatenation of gag-pol-env-tax ORFs. The numbers at the nodes correspond to the bootstrap value, obtained after 1000 repeats. The branch lengths are drawn to scale. HTLV-1c strains were used as outgroup. (**C**) Amino acid sequence of structural and enzymatic proteins GAG, PRO, POL, and ENV TAX, HBZ, p12, p30, and p13 from pBST and HTLV-1A prototype ATK. Mutation positions are indicated. (**D**) Amino acid sequence alignment compared to full-length genome sequences from Japanese HAM/TSP (n = 12) or Brazilian HAM/TSP (n = 10) (Supplemental S4–S12). See material and methods. Red triangle and (*) indicate stop codon.

We next performed a phylogenetic analysis of the pBST molecular clone. Results indicated that it is of the HTLV-1A Cosmopolitan Trans-continental subtype (Figure 2A and 2B), which is expected given the sample derived from a patient from French Guiana. Previous phylogenetic analyses of patients with HTLV-1 infection from Guyana strains indicate that Guiana strains primarily belong to the cosmopolitan subtype (HTLV-1 subtype A) and the prototype strain ATK [62]. ATK has been considered the HTLV-1A prototype sequence because it was the first ATL-derived full-length provirus cloned and is completely sequenced [63]. 

Each open reading frame (ORF) of pBST was translated and aligned to the HTLV-1A prototype ATK [63] (Supplemental S3) (Figure 2C). Because ATK is from a cosmopolitan Japanese isolate from a patient with ATL and our clone is from a cosmopolitan trans-continental subtype A from a HAM/TSP, we also compared the pBST sequence to the full-length genome sequence from Japanese HAM/TSP (*n* = 12) or Brazilian HAM/TSP (n = 10) (Supplemental S4–S12) (Figure 2D). The structural viral precursor protein GAG is synthesized as a polyprotein precursor processed by the viral protease into matrix p19, capsid p24, and nucleocapsid p15 [64]. The genetic alteration S8N present in pBST MA p19 has not been previously reported but is not predicted to affect the myristylation of MA at Gly 2. This mutation is not seen in HAM/TSP samples from Japan and Brazil or ACH or K30p molecular clones (Figure 2D and Supplemental S4). Genetic alterations of pBST p19 at residues A58 and R59 compared to ATK were also found in the MT-2 sequence but not seen in HAM/TSP samples from Japan and Brazil (Figure 2C and Supplemental S4). The L domain motif PPPY 118/125, which is essential for processing GAG by the viral protease, viral particle budding, and infectivity, is conserved in pBST [65]. Like HIV, a PTAP motif is found immediately downstream of the PPY motif in pBST [66]. Genetic alterations found in CA p24 have not been previously described. A variant at R368Q of the pBST NC p15 is located within the first of the two zinc fingers’ CCHC binding motifs. This variation appears specific to the pBST sequence. The biological significance of R368Q remains unclear since HTLV-1 CCHC motifs do not appear to function as efficiently as HIV for the RT process because of the presence of an acidic tail at the end of the HTLV-1 NC p15 [67]. In HTLV-1 proviruses, nonsense mutations preferentially accumulate in tryptophan codons since the tryptophan codon is TGG, and a G-to-A mutation generates either a TGA or a TAG stop codon. Since G-to-A mutations correspond to the preferred target sequence of human APOBEC3G (hA3G), the frequency of this mutation has been linked to APOBEC3G activity [68]. However, a previous study demonstrated that HTLV-1 resists G-to-A hypermutation elicited by hA3G, and additional studies reported that HTLV-1 was weakly susceptible to hA3G inhibition [69,70,71]. Surprisingly, alignment of the pBST to MT-2 or ATK sequences revealed a high frequency of G to A genetic alterations in the TSP subtype. 

The viral protease activity is essential for the maturation of gag-pol polypeptide and for virion maturation and infectivity. The variant observed in pBST G141E is not seen in HAM/TSP samples from Japan and Brazil (Figure 2D and Supplemental S5). However, the pBST sequence presents a G84R change compared to most HAM/TSP samples from Japan and Brazil (Figure 2C and Supplemental S5). Both ACH and K30p carry an Arg in this position. The POL gene had many variations compared to ATK (Figure 2B). However, only four amino acid changes were detected when compared with HAM/TSP samples from Japan and Brazil (Figure 2C and Supplemental S6). While G266C, I300V, and R384K were present in both Japanese and Brazilian samples, Q314R appears to be restricted to Brazilian samples (Supplemental S6). 

The viral envelope comprises the SU protein gp46, which contains the signal peptide (SP) and the receptor binding domain (RBD) and is associated with the TM gp21. The SU-TM complex is anchored to the infected cell membrane through the membrane-spanning region (MSR) [72]. SU-TM complexes are organized as trimers and transported to the surface of infected cells [73]. Overall, the Env gene was quite variable in pBST compared to ATK. pBST harbored a genetic alteration at F19L in the vicinity of the signal peptide cleavage site. However, this variant was also found in MT-2 cells, which are known to produce infectious viruses, and in HAM/TSP samples from Japan and Brazil (Supplemental S7). The amino acid region 197 to 216 has been shown to play a significant role in syncytia formation. Site-directed mutagenesis demonstrates that the glycosylation of each site is required for syncytium formation [74]. The binding of the RBD to viral receptors conveys a PRR-controlled signal to the C-terminal domain of gp46 (CTD), which leads to the activation of the fusion function of the gp21 fusion domain (FD) required for de novo infection. Surprisingly, early termination of the Env gene in pBST (W427*) has been previously reported in patients with ATL samples [68]. Additional studies have shown that a truncated HTLV-1 envelope protein, missing the hydrophobic MSR, remains associated with cellular membranes and virions [75]. HTLV-1 infectious virus producer cell line C10/MJ2 also carried a truncated env protein missing the MSR, and small amounts of such a truncated envelope glycoprotein were also found in the fusion-competent HTLV-1 producer cell line, MT-2 [75]. Importantly, a study demonstrated that resistance of various cell lines to HTLV-1 env-mediated syncytia was overcome by truncation of the C-terminal region in gp21/TM. Moreover, cell-to-cell virus transmission was significantly increased by truncating gp21/TM [76]. Whether pBST virus particles may be more easily transmitted to specific target cells remains to be investigated. We believe that the genetic alterations of K39E and S72G, found in the gp45 of pBST but not in ATK or MT-2 sequences, may represent a geographical polymorphism. Genebank database analyses show that S72G is more frequent in both HAM/TSP and ATL samples. However, K39E was found only in 1 ATL (GenBank: AAB39872.1) but was frequently found in HAM/TSP samples of patients from Martinique and French Guinea (GenBank: AAC21587.2; GenBank: AAU04899.1; GenBank: AAU04893.1; GenBank: AAU04891.1; GenBank: AAU04889.1; GenBank: AAC35405.2; GenBank: AAA85429.1) as well as HAM/TSP samples from Japan and Brazil (Figure 2C and Supplemental S7). Other genetic variations found in pBST (T89I, H137Q, R401C, P403L, and P411S) were also detected in the MT-2 sequence.

### 3.4. Characteristics of pBST Viral Regulatory Genes

Previous studies have shown that the viral immune-regulator p12 protein can be proteolytically cleaved at the amino terminus L9/10S and G29/30L and generate a shorter viral protein, known as the p8 protein [77,78]. Genetic studies have identified conserved mutations in p12. G29S, P34L, and S63P mutations were found to express a non-cleavable p12 [79]. Interestingly, pBST harbors both G29S and P34L mutations, suggesting that the processing of p12 into p8 may not occur in pBST. In addition, variants G29S and P34L that are present in p12 have been reported to have a high predictive value for HAM/TSP disease [80]. In fact, HAM/TSP samples from Japan and Brazil confirmed this observation (Figure 2C and Supplemental S6). An analysis of patients with HAM/TSP from Brazil previously showed that the frequency of the p12R88K mutation was rare; in support of this observation, an Arg was found in the pBST sequence and HAM/TSP samples from Japan and Brazil (Figure 2C and Supplemental S8) [81]. In silico analysis of HTLV-1 and simian t-cell leukemia virus (STLV-1), variants have suggested an absence of accessory proteins in certain viral variants. Analysis of HTLV-1A variants predicts that most patients with HTLV-1A harbor full-length p12, p13, and p30 viral accessory proteins, which is in contrast to HTLV-1B (sequencing predicts the absence of p12 and p30) and HTLV-1C (sequencing predicts the absence of p12 ATG) [82,83]. However, these assessments were not based on functional studies, as the lack of accessory proteins was only predicated by the presence of early stop codons, the absence of start codons, and the conservation of splicing sites from in silico sequence analysis. While our sequencing of pBST, an HTLV-1A variant, demonstrated intact p12, we found a truncation of p30 at W178*, which is like HTLV-1B variants, since this would result in the loss of p13. The sequence of the viral post-transcriptional negative factor p30 presented several genetic variations that were also detected in the MT-2 sequence, except for R36C, which was also observed in some of the HAM/TSP samples from Japan and Brazil (Figure 2C and Supplemental S9). Interestingly, early termination of p30 at positions W65*, W178*, and W190* has been previously found in patients with ATL samples [68,84]. In addition, W178* is also present in MT-2 cells, which chronically produce infectious virus (MT-2 GenBank: AB273635.1). Based on our previously published data, we do not think that early termination of p30 at W178 affects its negative regulatory functions on virus replication [85]. C-terminal truncations of p30 mutants still retained their nucleolar distribution, which would lead to retention of the *tax/rex* mRNA. However, the absence of the carboxy-terminus sequence would remove the mitogen-activated protein kinase (MAPK) Thr232 site, which was shown to be required for p30 delocalization from the nucleoli in response to DNA damage and is important for p30-mediated inhibition of DNA strand breaks (DSB) homologous repair (HR) [86]. This suggests that while pBST p30 still retains its inhibitory effect on the *tax/rex* mRNA, its role in DNA repair may be compromised.

Viral protein p13 primarily localizes to the mitochondria [87]. However, in the presence of Tax, p13 is partially ubiquitinated, stabilized, and rerouted to the nuclear speckles, where it inhibits Tax-mediated transcription and dampens virus expression [88]. The sequence of pBST revealed an early termination codon in amino acid position 21, likely resulting in the loss of all the domains essential for p13’s functional properties. We, therefore, anticipate that pBST does not express a functional p13 protein.

Viral transcriptional regulator Tax presented several genetic alterations. Variant G21R was unique to pBST and not seen in HAM/TSP samples from Japan and Brazil (Figure 2C and Supplemental S10) or ACH and K30p. Variant A220V was found in the non-canonical NF-κB2 activation domain, while another variant was found in the CREB activation domain at S304N. Both genetic alterations were also detected in the MT-2 sequence but not seen in HAM/TSP samples from Japan and Brazil (Figure 2C and Supplemental S10). However, pBST had specific variations at I326T, D339E, and I 334V (Figure 2C and Supplemental S7). Surprisingly, viral post-transcriptional factor Rex is the only gene with no specific alterations in pBST compared to the ATK-1 prototype, the MT-2 sequence, or HAM/TSP samples from Japan and Brazil (Supplemental S11). A previous variant, S13P (described in the antisense viral leucine zipper protein HBZ), was present in pBST as well as HAM/TSP samples from Japan and Brazil but not in any of the 20 ATL samples from Japan (Figure 2C and Supplemental S12). The R116Q alteration seen in pBST was detected in some HAM/TSP from Brazil but not in Japanese isolates (Supplemental S12). The significance of R116Q is currently unknown, but this mutation is located within the NLS of HBZ. The change of an Arginine, a negatively charged amino acid, for a Glutamine, a positively charged amino acid, may affect the sub-cellular localization of HBZ in pBST. Along these lines, a divergent pattern of HBZ distribution in HAM/TSP versus ATL, either to the cytoplasm or the nucleus, has been described [38]. Interestingly, pBST was isolated from a patient with HAM/TSP, and the HBZ protein has been found in the cytoplasm of patients with HTLV-1 infection with HAM/TSP [89]. These observations warrant additional studies.

### 3.5. pBST Viral Genes mRNA Expression and Virus Replication

**Figure 3:**
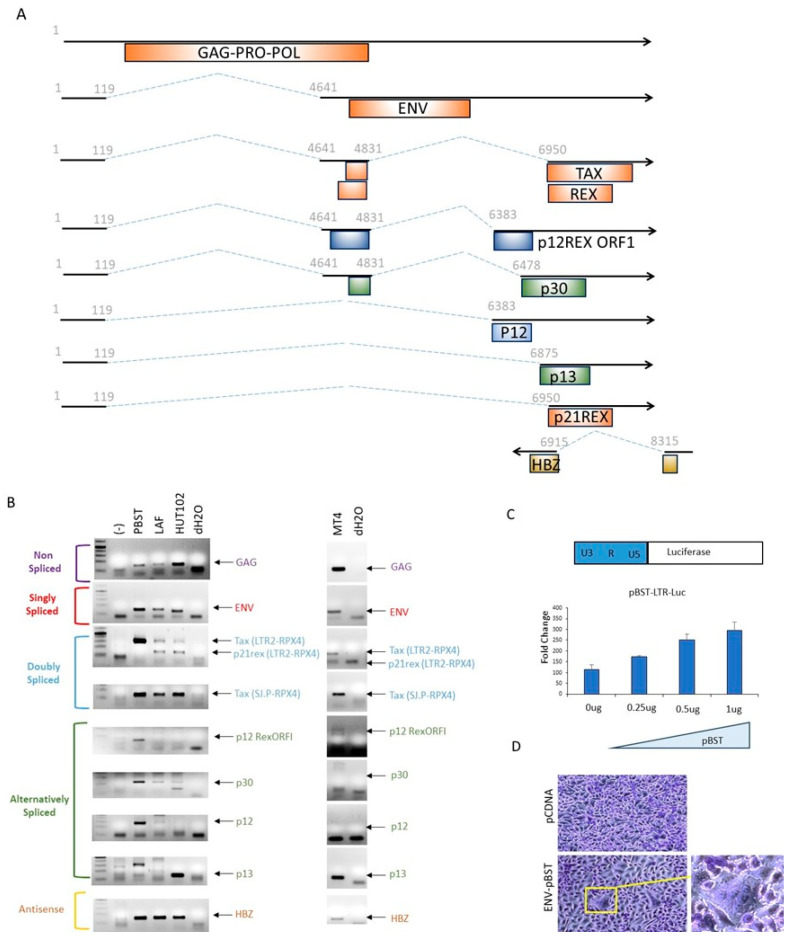
Expression of viral mRNAs from pBST. (**A**) Schematic representation of HTLV-1 mRNAs with positions of splice donors and splice acceptor sites noted. (**B**) pBST was transfected into 293T cells, and total RNAs were extracted after 48 h. “(-)” represents control, pCDNA 3.1 transfected 293T cells, and “PBST” represents pBST transfected 293T cells. Specific primers (Table 1) were used, and RT-PCR products were resolved onto agarose gels. p13 PCR products are approximately 130 bp. HTLV-1 transformed cells LAF, MT4, and HUT102 were used as controls. (**C**) Schematic of the pBST LTR cloned into the luciferase vector. Luciferase assays representing fold change activation of pBST-LTR-Luciferase activated by Tax produced in the context of the molecular clone. (**D**) The pBST envelope gene was cloned into pCDNA 3.1 expression vector and transfected into high-density HeLa cells. Syncytia were visualized after 48 h by staining with Crystal Violet.

The HTLV-1 provirus has a complex splicing pattern to allow the expression of structural and enzymatic genes, as well as viral regulatory proteins involved in virus replication, in vivo infection, and escape from the host defenses (Figure 3A). We next evaluated the expression of all known viral mRNAs following transient transfection of human epithelial cells. Viral mRNAs for *GAG-PRO-POL*, *ENV*, *Tax/Rex*, *p12 RexORF1*, *p30*, and *hbz* were all detected in cells transfected with pBST and the HTLV-1A molecular clone, ACH, which was used as a control (Figure 3B). HTLV-1 immortalized LAF or transformed HUT102 and MT4 cells were used as positive controls. LAF serves as a positive control for pBST since LAF is an HTLV-1 infected cell line derived from a French West Indian patient with TSP/HAM [77,90]. Our studies revealed that HTLV-1-transformed cells and ACH-transfected cells express a high ratio of *p21rex/tax* to *rex* mRNA. This is demonstrated by the competitive amplification of *tax/rex* and *p21rex* mRNA when using the primers LTR2 and RPX4, which can amplify both mRNAs. As previously reported, *p21rex* was the dominant mRNA expressed and outcompeted the *tax/rex* mRNA for amplification. The latter was readily detected when using primers specific for the *tax/rex* mRNA that were unable to amplify *p21rex*. 

Our results also demonstrate the absence of *p21Rex* mRNA from the pBST molecular clone, even though sequences for the splice acceptor (SA) sites for these mRNA were 100% conserved (Figure 3B). In addition, the ENV splice junction was also conserved, and no genetic alterations that could affect the RexRE sequence or folding were found. Therefore, it is unclear why the pBST molecular clone does not express *p21Rex* mRNA. It is unlikely that p21Rex protein can still be produced from the *tax/rex* mRNA by leaky internal initiation since the ATG start codon is in the 7th position and does not resemble the KOZAK sequence. 

Our studies also show that *p13* singly spliced mRNA was not detected in either ACH or pBST-expressing cells but was detected in LAF and MT4. This observation may indicate that p13 is expressed in the late stages after cellular immortalization or that some specific T cell splicing factors are responsible for the differential expression. Surprisingly, mRNA for *p12RexORF1*, *p12,* and *p30* were expressed at higher levels in pBST when compared to HTLV-1 transformed LAF and MT4 cells as well as ACH transfected cells. We next tested the ability of the pBST-derived Tax to activate the LTR and drive the expression of viral genes in its natural provirus context. The complete pBST LTR was PCR amplified and cloned into the pGL3 basic Luciferase reporter vector. Transient transfection assays of pBST-LTR-Luc with increasing amounts of pBST confirmed a dose-dependent increase in luciferase activity (Figure 3C). 

The residues between amino acids 100 and 200 of the SU are the targets of neutralizing antibodies [91]. In addition, mutations introduced into this region reduce the ability of HTLV-1 Env to induce syncytium formation and reduce virus infection. pBST carries a unique variant at H137Q and an early termination codon in gp21 that may affect conformation. We, therefore, tested Env-pBST fusogenic activity, an event required for viral infection. We cloned the Env-pBST gp62 into the pCDNA expression vector and transfected HeLa cells for syncytial formation assays. Results showed that Env-pBST was able to form syncytia, suggesting that mutations present in gp45 and gp21 may not affect this function (Figure 3D).

### 3.6. Production of Infectious Virus Particles by pBST

**Figure 4:**
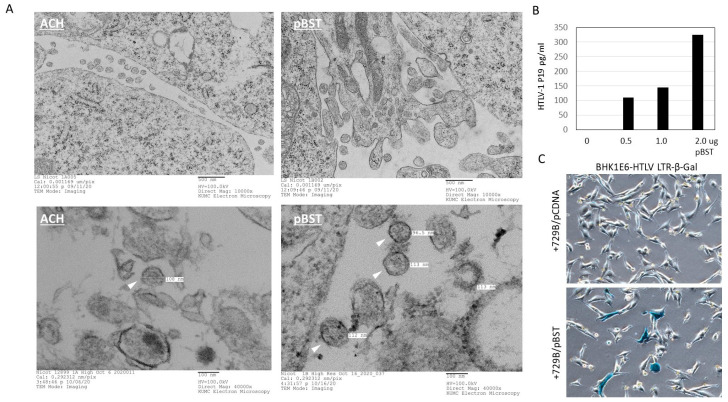
pBST produces infectious virus particles. (**A**) Electron microscopy images of 293T cells transfected with ACH or pBST. Digital images were acquired with an AMT digital camera, with magnification scale bars indicated in the figures. White triangles indicate virus particles, with size in nanometers (nm). (**B**) 293T cells were transfected with increasing amounts of pBST molecular clones. After 48 h, supernatant was cleared by centrifugation for 5 min at 10,000 rpm, and supernatant was tested for GAG p19 by ELISA. (**C**) 729B cells were transfected with pBST by Amaxa, and after 48 h, cells were co-cultivated with BHK1E6 HTLV-1LTR-LacZ reporter cells for 48 h. Cells were washed, fixed, and stained with X-Gal to reveal HTLV-1-infected beta-galactosidase-positive cells.

We next investigated if the pBST molecular clone could produce infectious virus particles in transient transfection assays in human fibroblast or 729B cells. We used the HTLV-1A molecular clone, ACH, as a positive control. Forty-eight hours after transfection of 293T cells with ACH or pBST molecular clones, cells were collected and prepared for electron microscopy. Our studies showed that both molecular clones can produce retrovirus particles at 90–120 nm in length, with a dense core characteristic of mature virus particles (Figure 4A). The release of virus particles in the supernatant of transfected cells was further confirmed by p19GAG ELISA assays (Figure 4B). To demonstrate the infectious nature of these particles, we used the previously characterized BHK1E6 reporter cells [42]. These cells harbor a full-length integrated HTLV-1-LTR fused to the beta-galactosidase gene. BHK1E6 cells present incredibly low basal activity, and only HTLV-1 infected cells are revealed by a blue color after X-gal staining. We used the Amaxa nucleofactor system for high transfection efficiency of 729B cells with the pBST and subsequently co-cultivated pBST-729B cells with BHK1E6. Our studies revealed the presence of Lac-Z, positive, single-cells, demonstrating the infectious nature of virus particles produced by pBST (Figure 4C).

### 3.7. mDC to T-Cell Virus Transmission Is Required for Long-Term Expansion and Immortalization of Human Primary T-Cells

We next investigated the capacity of the pBST molecular clone to immortalize human primary T cells in vitro and support secondary infection. As a control, we used the MT-2-derived HTLV-1A molecular clone ACH. Human PBMCs were activated with PHA for 48 h, washed, and cultured with IL-2-containing media for 72 h. We also immunoselected CD4+ T cells with Dynabeads. Both populations of cells were then transfected with pBST or ACH plasmids using the Amaxa Nucleofactor system and cultured in IL-2-containing media. These experiments resulted only in short-term proliferation of pBST or ACH cultures (3–4 months) before entering cell cycle arrest and crises resulting in cell death. 

**Figure 5:**
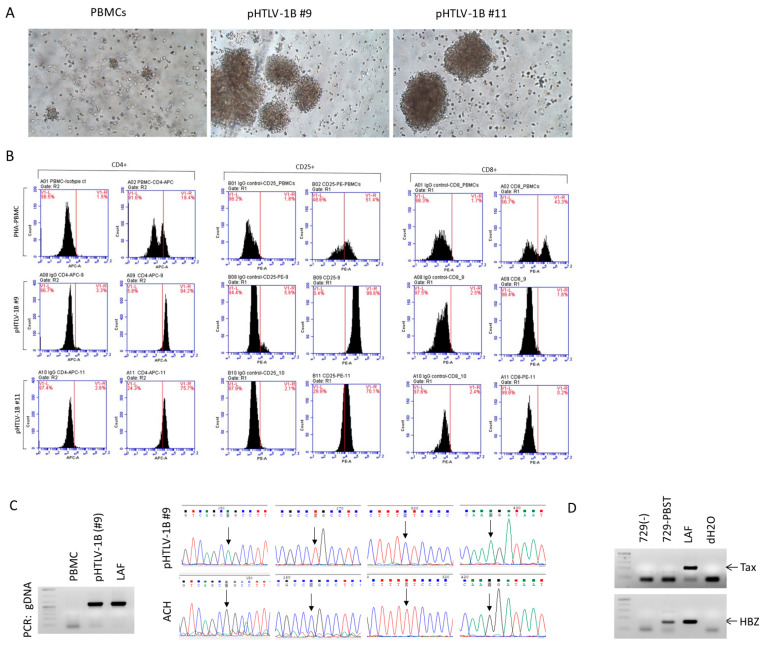
Immortalization of primary human T cell lines and secondary transmission to 729B cells. (**A**) Images of cultures from PBMCs at 3 weeks of culture and pBST immortalized T cell lines. (**B**) FACS analyses of cell surface markers for CD4, CD8, and CD25 expression on activated PBMCs or pBST immortalized cells. Cells were blocked for 30 min in BSA buffer and incubated for 2 h with the appropriate conjugated antibody. Cells were washed and fixed in 1% PFA overnight before analyses. CD4-APC (cat#551980) BD Pharmingen; IgG1-k-Isotype control-APC (cat#550854) BD Pharmingen. CD8-PE (cat#555367) BD Pharmingen; IgG1-k-Isotype control-PE (cat#555749) BD Pharmingen. Results were acquired on instrument BC-Accuri C6 Plus Flow Cytometer (BD Biosciences). (**C**) PCR analyses of genomic DNA from PBMC controls or PBMCs immortalized with pBST. HTLV-1 LAF cell line was used as a control. Chromatograms show specific genetic variations (black arrow) present in the pBST immortalized cell lines. The ACH sequence was used as a control. (**D**) RT-PCR analysis of *hbz* expression from 729B cell controls or 729B cells chronically infected with pBST. The HTLV-1 LAF cell line was used as a control.

Dendritic cells (DCs) are reported to replicate HTLV-1 and to support immortalization of primary T cells in vitro [92,93]. We separated myeloid dendritic cells (mDCs) from resting PBMCs using the adherence protocol, incubated with LPS for 24 h, and cultured in IL-4 and GM-CSF conditioned media for 3 days before mixing with ACH or pBST transfected CD4+ T cells. After 3 days, fresh PBMCs were added and cultured in the presence of IL-2. In this experimental setting, we were able to immortalize cells with both ACH and pBST. These cells have been maintained in culture with IL-2 for over 12 months and form the large clumps characteristic of HTLV-1-infected lines (Figure 5A). We next analyzed cell surface markers from PHA-activated PBMCs and two clones immortalized with pBST (Figure 5B). Our results confirmed a CD4+/CD25+ phenotype, which is characteristic of most HTLV-1 immortalized cell lines. The presence of integrated proviral DNA was confirmed by PCR amplification from high molecular weight genomic DNA (Figure 5C). This was further verified by sequencing the PCR product amplified in both pBST and ACH lines (Figure 5C). Surprisingly, none of the established T cell lines were positive by p19GAG ELISA or *Tax* expression. While not actively replicating the virus, it remains possible that heterologous contact allows virus reactivation and transfer, as this is typically the case for in vivo infections. Established T cell lines for ACH and pBST were treated with mitomycin C (100 µg/mL) for 4 h, washed, and co-cultivated with 729B cells, which have been shown to replicate HTLV-1. While pBST was transmitted from immortalized T-cells to 729B cells, virus expression could only be detected very early and was shut off within 3 weeks of culture. Like previous stable lines, only *hbz* expression was readily detectable in established 729B cells infected with pBST (Figure 5D).

## 4. Discussion

HTLV-1 infection is associated with lymphoproliferative or neurological diseases. Despite extensive research, it has been difficult to identify viral, host, and/or environmental factors associated with the development of one particular disease. While the progression of ATL disease is linked to methylation of FHIT, factors associated with the development of HAM/TSP are less clear. Our molecular clone has intriguing features previously associated with HAM/TSP.

HTLV-1 p12 G29S and P34L mutations are associated with disease phenotype and may influence the outcome of infection and the development of HAM/TSP [80]. In Japanese cohort analysis, the authors found a premature termination codon in p12 was observed in 5.6% of patients with HAM/TSP and in 4.9% of patients with ATL, but none was found in ACs, and in one patient with HAM/TSP, the p12 initiation codon was mutated [94]. Since these HTLV-1 variants appeared to have been transmitted in the subjects’ families, this implies that truncated p12 protein may retain essential viral functions or, in the case of the initiation codon, be produced from a differently spliced mRNA such as *p12RexORF1*. Although these alterations were not detected in ACs, it is very unlikely to contribute to disease progression given the low percentage detected in HAM/TSP and ATL. The importance of p12 in viral infectivity and maintenance in vivo has been demonstrated in rabbit and non-human primate animal models [61,95]. In support of these studies, HTLV-1C isolates do not have an ATG initiation codon for p12, further suggesting that this protein may be produced by alternative splicing, such as p12 RexORF1, and protease processing at L9/10S and/or G29S. Our studies suggest that pBST expresses p12 but not p8 protein. 

Another intriguing observation is the lack of *p21rex* mRNA expression. It is possible that p21rex is involved in viral latency by interfering with Rex multimerization functions and preventing the production of structural proteins. In contrast, the absence or low levels of p21rex may be associated with higher virus replication and increased inflammation. Chronically infected cell lines, which contain numerous defective proviruses, expressed 2-exon forms of pX mRNAs at significantly higher levels compared to cell lines that contain a single full-length provirus. Cells transfected with provirus expression plasmids expressed similar relative amounts of 3-exon pX mRNAs but lower levels of 2-exon mRNA forms compared to cells containing a single, full-length provirus [96]. Ectopic expression of hnRNP A1 did not affect pX splice site utilization but increased exon skipping, as the level of pX-p21rex mRNA was increased by almost 10-fold [96]. In addition, hnRNP A1 was shown to interfere with Rex’s functions by competitively binding to the RXRE [97]. We and others have found that p21Rex cannot export RNA from the nucleus to the cytoplasm and does not affect p27Rex export function [98,99,100]. However, another group reported that p21Rex stabilizes the unspliced transcript through inhibition of NMD [101]. These differences may be attributed to different experimental systems and warrant further analyses. 

In conclusion, we show that the pBST molecular clone is infectious and capable of immortalization and secondary transmission. Our studies further suggest that virus transmission from DC to T cells is required for efficient immortalization. Because immortalization is linked to proviral load and disease progression, we believe that the mechanism of virus entry may dictate the fate of infected cells and the risks of disease progression.

## Figures and Tables

**Table 1:** Primers.

	Forward	Reverse
GAG	pHTLV-1B GAG: CACCGGTCTGGATCTGTCCC	pHTLV-1B GAG: CGGGATCTGGGCTTGGGTTTGGATG
ENV	ENV: GTCTGTATCGATCGTGCCAGCC	ENV: GGTAACGTCAGGTGGGGGGC
Tax	SJP: CCTCAAGCGAGCTGCATGCCCAAG	RPX4: CACGTAGACTGGGTATCCG
p12	Uni-LTR: GCCGCCTCCCGCCTGTGGTG	Uni-p12: GAGTCCTTGGAGGCTGAACGGAGG
p13	LTR2: CCTGAGGCCGCCATCCACGCCGGTTG	Rp13: GGGCTGTTTCGATGCTTGCCTG
HBZ	Uni-HBZ(F): AATTGGTGGACGGGCTATTATCC	Uni-HBZ(R): CACGATGCGTTTCCCCGCGAGG
PBST-HBZ	pHTLV-1B HBZ: GCGGCCTCAGGGCTGTTTCGATGC	pHTLV-1B HBZ: GATAGCAAACCGTCAAGCACAGCTTC
p30	SJP: CCTCAAGCGAGCTGCATGCCCAAG	IK4: GCGCCGTGAGCGCAAGTGGAGAC
p21Rex	LTR2: CCTGAGGCCGCCATCCACGCCGGTTG	RPX4: CACGTAGACTGGGTATCCG
p12RexORFI	SJP: CCTCAAGCGAGCTGCATGCCCAAG	Rp12: GCAGGAGTTGGGGATTGATGGC
gDNA	Sense-RPX4: CCGTTCAGCCTCCAAGG	RPX4: CACGTAGACTGGGTATCCG

## Data Availability

All data generated or analyzed during this study are included in this published article (and its Supplementary Information files).

## Supplementary Materials

The following supporting information can be downloaded at: https://www.mdpi.com/article/10.3390/v16111755/s1, Supplemental S1 document represents the full nucleic acid sequence for the pBST molecular clone. Supplemental S2 document represents the pAB chimera molecular clone between ACH and pBST (Sal1-Cla1 substitution). Supplemental S3 document represents alignments for all genes of pBST genes with HTLV-1 prototype ATK. Supplemental S4 document represents an alignment of pBST GAG protein with HAM/TSP samples from Japan and Brazil as well as ACH and K30p molecular clones. Supplemental S5 document represents an alignment of pBST protease protein with HAM/TSP samples from Japan and Brazil as well as ACH and K30p molecular clones. Supplemental S6 document represents an alignment of pBST polymerase/integrase protein with HAM/TSP samples from Japan and Brazil as well as ACH and K30p molecular clones. Supplemental S7 document represents an alignment of pBST Env protein with HAM/TSP samples from Japan and Brazil as well as ACH and K30p molecular clones. Supplemental S8 document represents an alignment of pBST p12/p8 protein with HAM/TSP samples from Japan and Brazil as well as ACH and K30p molecular clones. Supplemental S9 document represents an alignment of p30/p13 GAG protein with HAM/TSP samples from Japan and Brazil, as well as ACH and K30p molecular clones. Supplemental S10 document represents an alignment of pBST Tax protein with HAM/TSP samples from Japan and Brazil as well as ACH and K30p molecular clones. Supplemental S11 document represents an alignment of pBST Rex protein with HAM/TSP samples from Japan and Brazil as well as ACH and K30p molecular clones. Supplemental S12 document represents an alignment of pBST HBZ protein with HAM/TSP samples from Japan and Brazil, ATL samples from Japan, as well as ACH and K30p molecular clones.
