# Supplementary material for: Characterization of HTLV-1 Infectious Molecular Clone Isolated from Patient with HAM/TSP and Immortalization of Human Primary T-Cell Lines
Source: Viruses. 2024 Nov 9;16(11):1755. doi: 10.3390/v16111755 (PMC11599126; doi:10.3390/v16111755)
Supplement: Supplementary file 1 [file viruses-16-01755-s001.zip › Supplemental S1 pBST SEQ.pdf]

pBST MOLECULAR CLONE SEQUENCE

(1)GGGGGCTTAGAGCCTCCCAGTGAAAAACATTTCCGCGAAACAGAAGTCTGAAAAGGTCAGGGCCCAGACTAAGGCTC
TGACGTCTCCCCCGGAGGGACAGCTCAGCACCGGCTCAGGCTAGGCCCTGACGTGTCCCCCTGAAGACAAATCATAAGC
TCAGACCTCCGGGAAGCCACCGGAACCACCCATTTCCTCCCCATGTTTGTCAAGCCGCCCTCAGGCGTTGACGACAACCCC
TCACCTCAAAAAACTTTTCATGGCACGCATATGGCTGAATAAACTAACAGGAGTCTATAAAAGCGTGGAGACAGTTCAGG
AGGGGGCTCGCATCTTTCCTTCACGCGCCCGCCGCCCTACCTGAGGCCGCCATCCACGCCGGTTGAGTCGCGTTCTGCCGC
CTCCCGCCTGTGGTGCCTCCTGAACTGCGTCCGCCGTCTAGGTAAGTTTAGAGCTCAGGTCGAGACCGGGCCTTTGTCCG
GCGCTCCCTTGGAGCCTACCTAGACTCAGCCGGCTCTCCACGCTTTGCCTGACCCTGCTTGCCCAACTCTGCGTCTTTGTTT
CGTTTTCTGTTCTGCGCCGCTACAGATCGAAAGTTCCACCCCTTTCCCTTTCATTCACGACTGACTGCCGGCTTGGCCCACG
GCCAAGTACCGGCGACTCCGTTGGCTCGGAGCCAGCGACAGCCCATTCTATAGCACTCTCCAGGAGAGAAATTTAGTACA
CAAGAGGGGGCTCACTCGGGGATAGAGGGGCTGGGCATGGCGGTAGGCAATGGGCCAAATCTTTTCCCGTAACGCTAG
CCCTATTCCGCGGCCGCCCCGGGGGCTGGCCGCTCATCACTGGCTTAACTTCCTCCAAGCGGCATATCGCCTAGAACCCGG
TCCCTCCAGTTACGATTTCCACCAGTTAAAAAAATTTCTTAAAATAGCTTTAGAAACACCGGTCTGGATCTGTCCCATTAAC
TACTCCCTCCTAGCCAGTCTACTCCCAAAAGGATACCCCGGCCGGGTGAATGAAATTTTACACATACTCATCCAAACCCAA
GCCCAGATCCCGTCCCGTCCCGCGCCACCGCCGCCGTCATCCCCCACCCACGACCCCCCGGATTCTGATCCACAAATCCCCC
CTCCCTATGTTGAGCCTACGGCCCCCCAAGTCCTTCCAGTCATGCACCCACATGGTGCCCCTCCCAACCATCGCCCATGGCA
AATGAAAGACCTACAGGCCATTAAGCAAGAAGTCTCCCAAGCAGCCCCTGGGAGCCCCCAGTTTATGCAGACCATCCGGC
TTGCGGTGCAGCAGTTTGACCCCACTGCCAAAGACCTCCAAGACCTCCTGCAGTACCTTTGCTCCTCCCTCGTGGCTTCCCT
CCATCACCAGCAGCTAGATAGCCTTATATCAGAGGCCGAAACCCGAGGTATTACAGGTTATAACCCCTTAGCCGGTCCCCT
CCGTGTCCAAGCCAACAATCCACAACAACAAGGATTAAGGCGAGAATACCAGCAACTCTGGCTCGCCGCCTTCGCCGCCC
TGCCAGGGAGTGCCAAAGACCCTTCCTGGGCCTCTATCCTCCAAGGCCTGGAGGAGCCTTACCACGCCTTCGTAGAACGC
CTCAACATAGCTCTTGACAATGGGCTGCCAGAAGGCACGCCCAAAGACCCCATCTTACGTTCCTTAGCCTACTCCAATGCA
AACAAAGAATGCCAAAAATTACTACAGGCCCGAGGACACACTAATAGCCCTCTAGGAGATATGTTGCGGGCTTGTCAGAC
CTGGACCCCCAAAGACAAAACCAAAGTGTTAGTTGTCCAGCCTAAAAAACCCCCCCCAAATCAGCCGTGCTTCCGGTGCG
GGAAAGCAGGCCACTGGAGTCAGGACTGCACTCAGCCTCGTCCCCCCCCCGGGCCATGCCCCCTATGTCAAGACCCAACT
CACTGGAAGCGAGACTGCCCCCGCCTAAAGCCCACTATCCCAGAACCAGAGCCAGAGGAAGATGCCCTCCTATTAGACCT
CCCCGCCGACATCCCACACCCAAAAAACTCCATAGGGGGGGAGGTTTAACCTCCCCCCCCACATTACAACAAGTCCTTCCT
AACCAAGACCCAGCATCTATTCTGCCAGTTATACCGTTAGATCCCGCCCGTCGGCCCGTAATTAAAGCCCAGGTTGACACC
CAGACCAGCCACCCAAAGACTATCGAAGCTTTACTAGATACAGGAGCAGACATGACAGTCCTTCCGATAGCCTTGTTCTCA
AGTAATACTCCCCTCAAAAATACATCCGTATTAGGGGCAGGAGGCCAAACCCAAGATCACTTTAAGCTCACCTCCCTTCCT
GTGCTAATACGCCTCCCTTTCCGGACAACGCCTATTGTTTTAACATCTTGCCTAGTTGATACCAAAAACAACTGGGCCATCA
TAGGTCGTGATGCCTTACAACAATGCCAAGGCGTCCTGTACCTCCCTGAGGCAAAAAGGCCGCCTGTAATCTTGCCAATAC
AGGCGCCAGCCGTCCTTGGGCTAGAACACCTCCCAAGGCCCCCCGAAATCAGCCAGTTCCCTTTAAACCAGAACGCCTCCA
GGCCTTGCAACACTTGGTCCGGAAGGCCCTGGAGGCAGGCCATATCGAACCCTACACCGGGCCAGGAAATAACCCAGTA
TTCCCAGTTAAAAAGGCCAATGGAACCTGGCGATTCATCCACGACCTGCGGGCCACTAACTCTCTAACCATAGATCTCTCA
TCATCTTCCCCCGGGCCCCCTGACTTGTCCAGCCTGCCAACTACACTAGCCCACTTGCAAACTATAGACCTTAAAGACGCCT
TTTTCCAAATCCCCTTACCTAAACAGTTCCAGCCCTACTTTGCTTTCACTGTCCCACAGCAGTGTAACTACGGCCCCGGCACT
AGATACGCCTGGAAAGTACTACCCCAAGGGTTTAAAAATAGTCCCACCCTGTTCGAAATGCAGCTGGCTCATATCCTGCAG
CCCATTCGGCAAGCTTTCCCCCAATGCACTATTCTTCAGTACATGGATGACATTCTCCTGGCAAGCCCCTCCCATGAGGACC
TACTACTACTCTCAGAGGCCACAATGGCTTCCCTAATCTCCCATGGGTTGCCTGTGTCCGAAAACAAAACCCAGCAAACCC
CTGGAACAATTAAGTTCCTAGGGCAAATAATTTCACCTAATCACCTCACTTATGATGCAGTCCCCACGGTACCTATACGGTC
CCGCTGGGCGCTACCTGAACTTCAAGCCCTACTTGGCGAGATTCAGTGGGTCTCCAAGGGAACTCCTACCTTACGCCAGCC
CCTTCACAGTCTCTACTGTGCCTTACAAAGGCATACTGATCCCCGAGACCAAATATATTTAAATCCTTCTCAAGTTCAATCAT
TAGTGCAGCTGCGGCAGGCCCTGTCACAGAACTGCCGCAGTAGACTAGTCCAAACCCTGCCCCTCCTAGGGGCTATTATG
CTGACCCTCACTGGCACCACTACTGTAGTGTTCCAGTCCAAGCAGCAGTGGCCACTTGTCTGGCTACATGCCCCCCTACCCC
ACACTAGCCAGTGCCCCTGGGGGCAGCTACTTGCCTCAGCTGTGTTATTACTCGACAAATACACCTTGCAATCCTATGGGC
TACTCTGCCAAACCATACATCATAACATCTCCACCCAAACCTTCAACCAATTCATTCAAACATCTGACCACCCCAGTGTTCCT
ATCTTACTCCATCACAGTCACCGATTCAAAAATTTATGTGCCCAAACTGGAGAACTTTGGAACACTTTTCTTAAAACAGCTG

CCCCATTGGCTCCTGTGAAAGCCCTCATGCCAGTGTTTACTCTTTCCCCGGTGATCCTAAACACCGCCCCCTGCCTGTTTTCA
GACGGATCTACCTCCCGGGCAGCCTATATTCTCTGGGACAAGCATATATTGTCACAAAGATCATTCCCCCTTCCGCCACCG
CACAAGTCGGCCCAACGGGCCGAACTTCTCGGACTTTTGCATGGCCTCTCCAGCGCCCGTTCGTGGCGCTGTCTCAACATA
TTTCTAGACTCCAAGTATCTTTATCATTACCTTCGGACCCTTGCCCTAGGCACCTTCCAAGGCAAGTCATCTCAGGCCCCCTT
TCAGGCCCTCCTGCCCCGCTTACTATCGCGTAAGGTCGTCTATTTGCACCACGTTCGCAGCCATACCAATCTACCTGATCCC
ATCTCCAGGCTCAACGCTCTCACAGATGCCCTACTAATCACCCCTGTCCTGCAGCTCTCTCCTGCAGAACTACACAGTTTCA
CCCATTGCGGACAGACGGCCCTCACATTGCAAGGGGCAACCACAACTGAGGCTTCCAATATCCTGCGCTCTTGCCACGCCT
GCCGCAAAAATAACCCACAACATCAGATGCCTCGGGGACACATCCGCCGTGGCCTACTTCCTAACCACATCTGGCAAGGC
GACATTACCCATTTCAAATATAAAAATACGCTGTATCGCCTTCATGTATGGGTAGACACCTTTTCAGGAGCCATCTCAGCTA
CCCAAAAGAGAAAAGAAACAAGCTCAGAAGCTATTTCCTCTTTGCTTCAGGCCATTGCCTATCTAGGCAAGCCTAGCTACA
TAAACACAGACAACGGCCCTGCCTATATTTCCCAAGACTTCCTCAATATGTGTACCTCCCTTGCTATTCGCCATACTACCCAT
GTCCCCTACAATCCAACCAGCTCAGGACTCGTAGAACGCTCTAATGGCATTCTTAAAACCCTACTATATAAGTACTTTACTG
ACAAACCCGACCTACCCATGGATAATGCTCTATCCATAGCCCTATGGACAATCAACCACCTGAATGTGTTAACCAACTGCC
ACAAAACCCGATGGCAGCTTCACCACTCCCCCCGACTCCAGCCGATCCCAGAGACACATTCCCTCAGCAATAAACAAACCC
ATTGGTATTATTTCAAGCTTCCTGGTCTTAATAGCCGCCAGTGGAAAGGACCACAGGAGGCTCTCCAAGAAGCTGCCGGC
GCTGCTCTCATCCCGGTAAGCGCTAGTTCTGCCCAGTGGATCCCGTGGAGACTCCTCAAGCGAGCTGC*ATGCCCAAGACCC*
*GTCGGAGGCCCCGCCGATCCCAAAGAAAAAGACCACCAACACC*ATGGGTAAGTTTCTCGCCACTTTAATTTTATTCTTCCA
GTTCTGCCCCCTCATCCTCGGTGATTACAGCCCCAGCTGCTGTACTCTCACAATTGGAGTCTCCTCATACCACTCTGAACCCT
GCAATCCTGCCCAGCCAGTTTGTTCGTGGACCCTCGACCTGCTGGCCCTTTCAGCAGATCAGGCCCTACAGCCCCCCTGCC
CTAATCTAGTAGGTTACTCTAGCTACCATGCCACCTATTCCCTATATCTATTCCCTCATTGGATTAAAAAGCCAAACCGAAA
TGGCGGAGGCTATTATTCAGCCTCTTATTCAGACCCTTGTTCCTTAAAGTGCCCATACCTGGGGTGCCAATCATGGACCTG
CCCCTATACAGGAGCCGTCTCCAGCCCCTACTGGAAGTTTCAGCAAGATGTCAATTTTACTCAAGAAGTTTCACGCCTCAAT
ATTAATCTCCATTTTTCGAAATGCGGTTTTCCCTTCTCCCTTCTAGTCGACGCTCCAGGATATGACCCCATCTGGTTCCTTAA
TACCGAACCCAGCCAACTGCCTCCCACCGCCCCTCCTCTACTCCCCCACTCTAACCTAGACCACATCCTCGAGCCCTCTATAC
CATGGAAATCAAAACTCCTGACCCTTGTCCAGTTAACCCTACAAAGCACTAATTATACTTGCATTGTCTGTATCGATCGTGC
CAGCCTATCCACTTGGCACGTCCTATACTCTCCCAACGTCTCTGTTCCATCCTCTTCTTCTACCCCCCTCCTTTACCCATCGTT
AGCGCTTCCAGCCCCCCACCTGACGTTACCATTTAACTGGACCCACTGCTTTGACCCCCAGATTCAAGCTATAGTCTCCTCC
CCCTGTCATAACTCCCTCATCCTGCCCCCCTTTTCCTTGTCACCTGTTCCCACCCTGGGATCCCGCTCCCGCCGAGCGGTACC
GGTGGCGGTCTGGCTTGTCTCCGCCCTGGCCATGGGAGCCGGAGTGGCTGGCGGGATTACCGGCTCCATGTCCCTCGCCT
CAGGAAAGAGCCTCCTACATGAGGTGGACAAAGATATTTCCCAATTAACTCAAGCAATAGTCAAAAACCACAAAAATCTA
CTCAAAATTGCGCAGTATGCTGCCCAGAACAGACGAGGCCTTGATCTCCTGTTCTGGGAGCAAGGAGGATTATGCAAAGC
ATTACAAGAACAGTGCTGTTTTCTAAATATTACTAATTCCCATGTCTCAATACTACAAGAAAGACCCCCCCTTGAGAATCGA
GTCCTGACTGGCTAGGGCCTTAACTGGGACCTTGGCCTCTCACAGTGGGCTCGAGAGGCCTTACAAACTGGAATCACCCT
TGTCGCGCTACTCCTTCTTGTTATCCTTGCAGGACCATGCATCCTCCGTCAGCTACGACACCTCCCCTCGCGCGTCAGATAC
CCCCATTACTCTCTTATAAACCCTGAGTCATCCCTGTAAACCAAGCACACAATTATTGCAACCACATCGCCTCCAGCCTCCCC
TGCCAATAATTAACCTCTCCCATTAAATCCTCCTTCTCCTGCAGCAACTTCCTCCGTTCAGCCTCCAAGGACTCCACCTCGCC
TTCCAACTGTCTAGTATAGCCATCAATCCCCAACTCCTGCATTTTTTCTTTCCTAGCACTATGCTGTTTCGCCTTCTCAGCCCC
TTGTCTCCACTTGCGCTCACGGCGCTCCTGCTCTTCCTGCTTTCTCCGGGCGACGTCAGCAGCCTTCTTCTCCGCCTGCCTCC
TGCGCCGTGCCTTCTCCTCTTCCTTCCTTTTCAAATACTCAGCAATCTGCTTTTCCTCCTCTTTCTCCCGCTCTTTTTTTCGCTT
CCTCTTCTCCTCAGCCCGTCGCTGCCGATCACGATGCGTTTCCCCGCGAGGTGGCGCTTTCTCCCCTGGAGGGCCCCGTCG
CAGCCGGCCGCGGCTTTCCTCTTCTAAGGATAGCAAACCGTCAAGCACAGCTTCCTCCTCCTCCTTGTCCTTTAACTCTTCCT
CCAAAGATAATAGCCCGTCCACCAATTCCTCCACCAGCAGGTCCTCCGGGCATGGCACAGGCAAGCATCGAAACAGCCCT
ACAGATACAAAGTTAACCATGCTTATTATCAGCCCACTTCCCAGGGTTTGGACAGAGTCTTCTTTTCGGATACCCAGTCTAC
GTGTTTAGAGACTGTGTACAAGGCGACTGGTGCCCCATCTCTGGGGGACTATGTTCGGCCCGCCTACATCGTCACGCCCTA
CTGGCCACCTGTCCAGAGCATCAGATCACCTGGGACCCCATCGATGGACGCGTTATCGGCTCAGCTCTACAGTTCCTTATC
CCTCGACTCCCCTCCTTCCCCACCCAGAGAACCTCTAAGACCCTCAAGGTCCTTACCCCGCCAATCACTCATACAACCCCCA
ACATTCCACCCTCCTTCCTCCAGGCCATGCGCAAATACTCCCCCTTCCGAAATGGATACATGGAACCCACCCTTGGGCAGC
ACCTCCCAACCCTGTCTTTTCCAGACCCCGGACTCCGGCCCCAAAACCTGTACACCCTCTGGGGAGGCTCCGTTGTCTGCAT
GTACCTCTACCAGCTTTCCCCCCCCATCACCTGGCCCCTCCTGCCCCACGTGATTTTTTGCCACCCCGGCCAGCTCGGGGCC
TTCCTCACCAATGTTCCCTACAAGCGAATAGAAGAACTCCTCTATAAAATTTCCCTTACCACAGGGGCCCTAATAATTCTAC

CCGAAGACTGTTTGCCCACCACCCTTTTCCAGCCTGTTAGGGCACCCGTCACGCTAACAGCCTGGCAAAACGGCCTCCTTC
CGTTCCACTCAACCCTCACCACTCCAGGCCTTATTTGGACATTTACCGATGGCACGCCTATGATTTCCGGGCCCTGCCCTAA
AGATGGCCAGCCATCTTTAGTACTACAGTCCTCCTCCTTTATATTTCACAAATTTCAAACCAAGGCCTACCACCCCTCATTTC
TACTCTCACACGGCCTCATACAGTACTCTTCCTTTCATAATTTACATCTCCTGTTTGAAGAATACACCAACATCCCCATTTCTC
TACTTTTTAACGAAAAAGAGGCAGATGACAATGACCATGAGCCCCAAATATCCCCCGGGGGCTTAGAGCCTCCCAGTGAA
AAACATTTCCGCGAAACAGAAGTCTGAAAAGGTCAGGGCCCAGACTAAGGCTCTGACGTCTCCCCCCCGGAGGGACAGC
TCAGCACCGGCTCAGGCTAGGCCCTGACGTGTCCCCCTGAAGACAAATCATAAGCTCAGACCTCCGGGAAGCCACCGGAA
CCACCCATTTCCTCCCCATGTTTGTCAAGCCGCCCTCAGGCGTTGACGACAACCCCTCACCTCAAAAAACTTTTCATGGCAC
GCATATGGCTGAATAAACTAACAGGAGTCTATAAAAGCGTGGAGACAGTTCAGGAGGGGGCTCGCATCTTTCCTTCACGC
GCCCGCCGCCCTACCTGAGGCCGCCATCCACGCCGGTTGAGTCGCGTTCTGCCGCCTCCCGCCTGTGGTGCCTCCTGAACT
GCGTCCGCCGTCTAGGTAAGTTTAGAGCTCAGGTCGAGACCGGGCCTTTGTCCGGCGCTCCCTTGGAGCCTACCTAGACT
CAGCCGGCTCTCCACGCTTTGCCTGACCCTGCTTGCCCAACTCTGCGTCTTTGTTTCGTTTTCTGTTCTGCGCCGCTACAGAT
CGAAAGTTCCACCCCTTTCCCTTTCATTCACGACTGACTGCCGGCTTGGCCCACGGCCAAGTACCGGCGACTCCGTTGGCT
CGGAGCCAGCGACAGCCCATTCTATAGCACTCTCCAGGAGAGAAATTTAGTACACA **(9024 - end)**
