# Supplementary material for: Characterization of HTLV-1 Infectious Molecular Clone Isolated from Patient with HAM/TSP and Immortalization of Human Primary T-Cell Lines
Source: Viruses. 2024 Nov 9;16(11):1755. doi: 10.3390/v16111755 (PMC11599126; doi:10.3390/v16111755)
Supplement: Supplementary file 1 [file viruses-16-01755-s001.zip › Supplemental S10 Tax align.pdf]

HTLV-1 TAX ALIGNMENTS BETWEEN JAPANESE AND BRAZILIAN HAM/TSP ISOLATES (FULL GENOME SEQUENCE) AND OTHER HTLV-1 MOLECULAR CLONES

**PBST**	MAHFPGFGQSLLFGYPVYVFRDCVQGDWCPISGGLCSARLHRHALLATCPEHQITWDPIDGRVIGSALQFLIPRLPSFPTQRTSKTLKVLTPPITHTTPNI

**HAM1**	MAHFPGFGQSLLFGYPVYVFGDCVQGDWCPISGGLCSARLHRHALLATCPEHQITWDPIDGRVIGSALQFLIPRLPSFPTQRTSKTLKVLTPPITHTTPNI
**HAM2**	MAHFPGFGQSLLFGYPVYVFGDCVQGDWCPISGGLCSARLHRHALLATCPEHQITWDPIDGRVIGSALQFLIPRLPSFPTQRTSKTLKVLTPPITHTTPNI
**HAM3**	MAHFPGFGQSLLFGYPVYVFGDCVQGDWCPISGGLCSARLHRHALLATCPEHQITWDPIDGRVIGSALQFLIPRLPSFPTQRTSKTLKVLTPPITHTTPNI
**HAM4**	MAHFPGFGQSLLFGYPVYVFGDCVQGDWCPISGGLCSARLHRHALLATCPEHQITWDPIDGRVIGSALQFLIPRLPSFPTQRTSKTLKVLTPPITHTTPNI
**HAM5**	MAHFPGFGQSLLFGYPVYVFGDCVQGDWCPISGGLCSARLHRHALLATCPEHQITWDPIDGRVIGSALQFLIPRLPSFPTQRTSKTLKVLTPPITHTTPNI
**HAM6**	MAHFPGFGQSLLFGYPVYVFGDCVQGDWCPISGGLCSARLHRHALLATCPEHQITWDPIDGRVIGSALQFLIPRLPSFPTQRTSKTLKVLTPPITHTTPNI
**HAM7**	MAHFPGFGQSLLFGYPVYVFGDCVQGDWCPISGGLCSARLHRHALLATCPEHQITWDPIDGRVIGSALQFLIPRLPSFPTQRTSKTLKVLTPPITHTTPNI
**HAM8**	MAHFPGFGQSLLFGYPVYVFGDCVQGDWCPISGGLCSARLHRHALLATCPEHQITWDPIDGRVIGSALQFLIPRLPSFPTQRTSKTLKVLTPPITHTTPNI
**HAM9**	MAHFPGFGQSLLFGYPVYVFGDCVQGDWCPISGGLCSARLHRHALLATCPEHQITWDPIDGRVIGSALQFLIPRLPSFPTQRTSKTLKVLTPPITHTTPNI
**HAM10**	MAHFPGFGQSLLFGYPVYVFGDCVQGDWCPISGGLCSARLHRHALLATCPEHQITWDPIDGRVIGSALQFLIPRLPSFPTQRTSKTLKVLTPPITHTTPNI
**HAM11**	MAHFPGFGQSLLFGYPVYVFGDCVQGDWCPISGGLCSARLHRHALLATCPEHQITWDPIDGRVIGSALQFLIPRLPSFPTQRTSKTLKVLTPPITHTTPNI
**HAM12**	MAHFPGFGQSLLFGYPVYVFGDCVQGDWCPISGGLCSARLHRHALLATCPEHQITWDPIDGRVIGSALQFLIPRLPSFPTQRTSKTLKVLTPPITHTTPNI

**HAM1**	MAHFPGFGQSLLFGYPVYVFGDCVQGDWCPISGGLCSARLHRHALLATCPEHQITWDPIDGRVIGSALQFLIPRLPSFPTQRTSKTLKVLTPPITHTTPNI
**HAM2**	MAHFPGFGQSLLFGYPVYVFGDCVQGDWCPISGGLCSARLHRHALLATCPEHQITWDPIDGRVIGSALQFLIPRLPSFPTQRTSKTLKVLTPPITHTTPNI
**HAM3**	MAHFPGFGQSLLFGYPVYVFGDCVQGDWCPISGGLCSARLHRHALLATCPEHQITWDPIDGRVIGSALQFLIPRLPSFPTQRTSKTLKVLTPPITHTTPNI
**HAM4**	MAHFPGFGQSLLFGYPVYVFGDCVQGDWCPISGGLCSARLHRHALLATCPEHQITWDPIDGRVIGSALQFLIPRLPSFPTQRTSKTLKVLTPPITHTTPNI
**HAM5**	MAHFPGFGQSLLFGYPVYVFGDCVQGDWCPISGGLCSARLHRHALLATCPEHQITWDPIDGRVIGSALQFLIPRLPSFPTQRTSKTLKVLTPPITHTTPNI
**HAM6**	MAHFPGFGQSLLFGYPVYVFGDCVQGDWCPISGGLCSARLHRHALLATCPEHQITWDPIDGRVIGSALQFLIPRLPSFPTQRTSKTLKVLTPPITHTTPNI
**HAM7**	MAHFPGFGQSLLFGYPVYVFGDCVQGDWCPISGGLCSARLHRHALLATCPEHQITWDPIDGRVIGSALQFLIPRLPSFPTQRTSKTLKVLTPPITHTTPNI
**HAM8**	MAHFPGFGQSLLFGYPVYVFGDCVQGDWCPISGGLCSARLHRHALLATCPEHQITWDPIDGRVIGSALQFLIPRLPSFPTQRTSKTLKVLTPPITHTTPNI
**HAM9**	MAHFPGFGQSLLFGYPVYVFGDCVQGDWCPISGGLCSARLHRHALLATCPEHQITWDPIDGRVIGSALQFLIPRLPSFPTQRTSKTLKVLTPPITHTTPNI
**HAM10**	MAHFPGFGQSLLFGYPVYVFGDCVQGDWCPISGGLCSARLHRHALLATCPEHQITWDPIDGRVIGSALQFLIPRLPSFPTQRTSKTLKVLTPPITHTTPNI

**ACH**	MAHFPGFGQSLLFGYPVYVFGDCVQGDWCPISGGLCSARLHRHALLATCPEHQITWDPIDGRVIGSALQFLIPRLPSFPTQRTSKTLKVLTPPITHTTPNI
**K30p**	MAHFPGFGQSLLFGYPVYVFGDCVQGDWCPISGGLCSARLHRHALLATCPEHQITWDPIDGRVIGSALQFLIPRLPSFPTQRTSKTLKVLTPPITHTTPNI

**pBST**	PPSFLQAMRKYSPFRNGYMEPTLGQHLPTLSFPDPGLRPQNLYTLWGGSVVCMYLYQLSPPITWPLLPHVIFCHPGQLGAFLTNVPYKRIEELLYKISLTT

**HAM1**	PPSFLQAMRKYSPFRNGYMEPTLGQHLPTLSFPDPGLRPQNLYTLWGGSVVCMYLYQLSPPITWPLLPHVIFCHPGQLGAFLTNVPYKRIEELLYKISLTT
**HAM2**	PPSFLQAMRKYSPFRNGYMEPTLGQHLPTLSFPDPGLRPQNLYTLWGGSVVCMYLYQLSPPITWPLLPHVIFCHPGQLGAFLTNVPYKRIEELLYKISLTT
**HAM3**	PPSFLQAMRKYSPFRNGYMEPTLGQHLPTLSFPDPGLRPQNLYTLWGGSVVCMYLYQLSPPITWPLLPHVIFCHPGQLGAFLTNVPYKRIEELLYKISLTT
**HAM4**	PPSFLQAMRKYSPFRNGYMEPTLGQHLPTLSFPDPGLRPQNLYTLWGGSVVCMYLYQLSPPITWPLLPHVIFCHPGQLGAFLTNVPYKRIEELLYKISLTT
**HAM5**	PPSFLQAMRKYSPFRNGYMEPTLGQHLPTLSFPDPGLRPQNLYTLWGGSVVCMYLYQLSPPITWPLLPHVIFCHPGQLGAFLTNVPYKRIEELLYKISLTT
**HAM6**	PPSFLQAMRKYSPFRNGYMEPTLGQHLPTLSFPDPGLRPQNLYTLWGGSVVCMYLYQLSPPITWPLLPHVIFCHPGQLGAFLTNVPYKRIEELLYKISLTT
**HAM7**	PPSFLQAMRKYSPFRNGYMEPTLGQHLPTLSFPDPGLRPQNLYTLWGGSVVCMYLYQLSPPITWPLLPHVIFCHPGQLGAFLTNVPYKRIEELLYKISLTT
**HAM8**	PPSFLQAMRKYSPFRNGYMEPTLGQHLPTLSFPDPGLRPQNLYTLWGGSVVCMYLYQLSPPITWPLLPHVIFCHPGQLGAFLTNVPYKRIEELLYKISLTT
**HAM9**	PPSFLQAMRKYSPFRNGYMEPTLGQHLPTLSFPDPGLRPQNLYTLWGGSVVCMYLYQLSPPITWPLLPHVIFCHPGQLGAFLTNVPYKRIEELLYKISLTT
**HAM10**	PPSFLQAMRKYSPFRNGYMEPTLGQHLPTLSFPDPGLRPQNLYTLWGGSVVCMYLYQLSPPITWPLLPHVIFCHPGQLGAFLTNVPYKRIEELLYKISLTT
**HAM11**	PPSFLQAMRKYSPFRNGYMEPTLGQHLPTLSFPDPGLRPQNLYTLWGGSVVCMYLYQLSPPITWPLLPHVIFCHPGQLGAFLTNVPYKRIEELLYKISLTT
**HAM12**	PPSFLQAMRKYSPFRNGYMEPTLGQHLPTLSFPDPGLRPQNLYTLWGGSVVCMYLYQLSPPITWPLLPHVIFCHPGQLGAFLTNVPYKRIEELLYKISLTT

**HAM1**	PPSFLQAMRKYSPFRNGYMEPTLGQHLPTLSFPDPGLRPQNLYTLWGGSVVCMYLYQLSPPITWPLLPHVIFCHPGQLGAFLTNVPYKRIEELLYKISLTT
**HAM2**	PPSFLQAMRKYSPFRNGYMEPTLGQHLPTLSFPDPGLRPQNLYTLWGGSVVCMYLYQLSPPITWPLLPHVIFCHPGQLGAFLTNVPYKRIEELLYKISLTT
**HAM3**	PPSFLQAMRKYSPFRNGYMEPTLGQHLPTLSFPDPGLRPQNLYTLWGGSVVCMYLYQLSPPITWPLLPHVIFCHPGQLGAFLTNVPYKRIEELLYKISLTT
**HAM4**	PPSFLQAMRKYSPFRNGYMEPTLGQHLPTLSFPDPGLRPQNLYTLWGGSVVCMYLYQLSPPITWPLLPHVIFCHPGQLGAFLTNVPYKRIEELLYKISLTT
**HAM5**	PPSFLQAMRKYSPFRNGYMEPTLGQHLPTLSFPDPGLRPQNLYTLWGGSVVCMYLYQLSPPITWPLLPHVIFCHPGQLGAFLTNVPYKRIEELLYKISLTT
**HAM6**	PPSFLQAMRKYSPFRNGYMEPTLGQHLPTLSFPDPGLRPQNLYTLWGGSVVCMYLYQLSPPITWPLLPHVIFCHPGQLGAFLTNVPYKRIEELLYKISLTT
**HAM7**	PPSFLQAMRKYSPFRNGYMEPTLGQHLPTLSFPDPGLRPQNLYTLWGGSVVCMYLYQLSPPITWPLLPHVIFCHPGQLGAFLTNVPYKRIEELLYKISLTT
**HAM8**	PPSFLQAMRKYSPFRNGYMEPTLGQHLPTLSFPDPGLRPQNLYTLWGGSVVCMYLYQLSPPITWPLLPHVIFCHPGQLGAFLTNVPYKRIEELLYKISLTT
**HAM9**	PPSFLQAMRKYSPFRNGYMEPTLGQHLPTLSFPDPGLRPQNLYTLWGGSVVCMYLYQLSPPITWPLLPHVIFCHPGQLGAFLTNVPYKRIEELLYKISLTT
**HAM10**	PPSFLQAMRKYSPFRNGYMEPTLGQHLPTLSFPDPGLRPQNLYTLWGGSVVCMYLYQLSPPITWPLLPHVIFCHPGQLGAFLTNVPYKRIEELLYKISLTT

**ACH**	PPSFLQAMRKYSPFRNGYMEPTLGQHLPTLSFPDPGLRPQNLYTLWGGSVVCMYLYQLSPPITWPLLPHVIFDHPGQLGAFLTNVPYKRIEELLYKISLTT
**K30p**	PPSFLQAMRKYSPFRNGYMEPTLGQHLPTLSFPDPGLRPQNLYTLWGGSVVCMYLYQLSPPITWPLLPHVIFCHPGQLGAFLTNVPYKRIEELLYKISLTT

**pBST**	GALIIILPEDCLPTTLFQPVRAPVTLTAWQNGLLPFHSTLTTPGLIWTFTDGTPMISGPCPKDGQPSLVLQSSSFIFHKFQTKAYHPSFLLSHGLIQYSSFH
**HAM1**	GALIIILPEDCLPTTLFQPVRAPVTLTAWQNGLLPFHSTLTTPGLIWTFTDGTPMISGPCPKDGQPSLVLQSSSFIFHKFQTKAYHPSFLLSHGLIQYSSFH
**HAM2**	GALIIILPEDCLPTTLFQPVRAPVTLTAWQNGLLPFHSTLTTPGLIWTFTDGTPMISGPCPKDGQPSLVLQSSSFIFHKFQTKAYHPSFLLSHGLIQYSSFH
**HAM3**	GALIIILPEDCLPTTLFQPVRAPVTLTAWQNGLLPFHSTLTTPGLIWTFTDGTPMISGPCPKDGQPSLVLQSSSFIFHKFQTKAYHPSFLLSHGLIQYSSFH
**HAM4**	GALIIILPEDCLPTTLFQPVRAPVTLTAWQNGLLPFHSTLTTPGLIWTFTDGTPMISGPCPKDGQPSLVLQSSSFIFHKFQTKAYHPSFLLSHGLIQYSSFH
**HAM5**	GALIIILPEDCLPTTLFQPVRAPVTLTAWQNGLLPFHSTLTTPGLIWTFTDGTPMISGPCPKDGQPSLVLQSSSFIFHKFQTKAYHPSFLLSHGLIQYSSFH
**HAM6**	GALIIILPEDCLPTTLFQPVRAPVTLTAWQNGLLPFHSTLTTPGLIWTFTDGTPMISGPCPKDGQPSLVLQSSSFIFHKFQTKAYHPSFLLSHGLIQYSSFH
**HAM7**	GALIIILPEDCLPTTLFQPVRAPVTLTAWQNGLLPFHSTLTTPGLIWTFTDGTPMISGPCPKDGQPSLVLQSSSFIFHKFQTKAYHPSFLLSHGLIQYSSFH
**HAM8**	GALIIILPEDCLPTTLFQPVRAPVTLTAWQNGLLPFHSTLTTPGLIWTFTDGTPMISGPCPKDGQPSLVLQSSSFIFHKFQTKAYHPSFLLSHGLIQYSSFH
**HAM9**	GALIIILPEDCLPTTLFQPVRAPVTLTAWQNGLLPFHSTLTTPGLIWTFTDGTPMISGPCPKDGQPSLVLQSSSFIFHKFQTKAYHPSFLLSHGLIQYSSFH
**HAM10**	GALIIILPEDCLPTTLFQPVRAPVTLTAWQNGLLPFHSTLTTPGLIWTFTDGTPMISGPCPKDGQPSLVLQSSSFIFHKFQTKAYHPSFLLSHGLIQYSSFH
**HAM11**	GALIIILPEDCLPTTLFQPVRAPVTLTAWQNGLLPFHSTLTTPGLIWTFTDGTPMISGPCPKDGQPSLVLQSSSFIFHKFQTKAYHPSFLLSHGLIQYSSFH
**HAM12**	GALIIILPEDCLPTTLFQPVRAPVTLTAWQNGLLPFHSTLTTPGLIWTFTDGTPMISGPCPKDGQPSLVLQSSSFIFHKFQTKAYHPSFLLSHGLIQYSSFH

**HAM1**	GALIIILPEDCLPTTLFQPVRAPVTLTAWQNGLLPFHSTLTTPGLIWTFTDGTPMISGPCPKGGQPSLVLQSSSFIFHKFQTKAYHPSFLLSHGLIQYSSFH
**HAM2**	GALIIILPEDCLPTTLFQPVRAPVTLTAWQNGLLPFHSTLTTPGLIWTFTDGTPMISGPCPKDGQPSLVLQSSSFIFHKFQTKAYHPSFLLSHGLIQYSSFH
**HAM3**	GALIIILPEDCLPTTLFQPVRAPVTLTAWQNGLLPFHSTLTTPGLIWTFTDGTPMISGPCPKDGQPSLVLQSSSFIFHKFQTKAYHPSFLLSHGLIQYSSFH
**HAM4**	GALIIILPEDCLPTTLFQPVRAPVTLTAWQNGLLPFHSTLTTPGLIWTFTDGTPMISGPCPKDGQPSLVLQSSSFIFHKFQTKAYHPSFLLSHGLIQYSSFH
**HAM5**	GALIIILPEDCLPTTLFQPVRAPVTLTAWQNGLLPFHSTLTTPGLIWTFTDGTPMISGPCPKDGQPSLVLQSSSFIFHKFQTKAYHPSFLLSHGLIQYSSFH
**HAM6**	GALIIILPEDCLPTTLFQPVRAPVTLTAWQNGLLPFHSTLTTPGLIWTFTDGTPMISGPCPKDGQPSLVLQSSSFIFHKFQTKAYHPSFLLSHGLIQYSSFH
**HAM7**	GALIIILPEDCLPTTLFQPVRAPVTLTAWQNGLLPFHSTLTTPGLIWTFTDGTPMISGPCPKDGQPSLVLQSSSFIFHKFQTKAYHPSFLLSHGLIQYSSFH
**HAM8**	GALIIILPEDCLPTTLFQPVRAPVTLTAWQNGLLPFHSTLATPGLIWTFTDGTPMISGPCPKDGQPSLVLQSSSFIFHKFQTKAYHPSFLLSHGLIQYSSFH
**HAM9**	GALIIILPEDCLPTTLFQPVRAPVTLTAWQNGLLPFHSTLTTPGLIWTFTDGTPMISGPCPKDGQPSLVLQSSSFIFHKFQTKAYHPSFLLSHGLIQYSSFH
**HAM10**	GALIIILPEDCLPTTLFQPVRAPVTLTAWQNGLLPFHSTLTTPGLIWTFTDGTPMISGPCPKDGQPSLVLQSSSFIFHKFQTKAYHPSFLLSHGLIQYSSFH

**ACH**	GALIIILPEDCLPTTLFQPVRAPVTLTAWQNGLLPFHSTLTTPGLIWTFTDGTPMISGPCPKDGQPSLVLQSSSFIFHKFQTKAYHPSFLLSHGLIQYSSFH
**K30p**	GALIIILPEDCLPTTLFQPVRAPVTLTAWQNGLLPFHSTLTTPGLIWTFTDGTPMISGPCPKDGQPSLVLQSSSFIFHKFQTKAYHPSFLLSHGLIQYSSFH

**pBST**	NLHLLFEEYTNIPTSLLFNEKEADDNEHEPQVSPGGLEPPSEKHFRETEV
**HAM1**	NLHLLFEEYTNIPISLLFNKKEADDNDHEPEISPGGLEPPSEKHFRETEV
**HAM2**	NLHLLFEEYTNIPISLLFNEKEADDNEHEPQISPGGLEPPSEKHFRETEV
**HAM3**	NLHLLFEEYTNIPISLLFNEKEADDNDHEPQISPGGLEPPSEKHFRETEV
**HAM4**	NLHLLFEEYTNIPISLLFNEKEADDNDHEPQISPGGLEPPSEKHFRETEV
**HAM5**	NLHLLFEEYTNIPISLLFNEKEADDNDHEPQISPGGLEPPSEKHFRETEV
**HAM6**	NLHLLFEEYTNIPISLLFNEKEADDNDHEPQISPGGLEPPSEKHFRETEV
**HAM7**	NLHLLFEEYTNIPISLLFNEKEADDNDHEPEISPGGLEPPSEKHFRETEV
**HAM8**	NLHLLFEEYTNIPISLLFNEKEADDNDHEPEISPGGLEPPSEKHFRETEV
**HAM9**	NLHLLFEEYTNIPISLLFNEKEADDNDHEPEISPGGLEPPSEKHFRETEV
**HAM10**	NLHLLFEEYTNIPISLLFNEKEADDNDHEPQISPGGLEPPSEKHFRETEV
**HAM11**	NLHLLFEEYTNIPISLLFNEKEADDNDHEPQISPGGLEPPSEKHFRETEV
**HAM12**	NLHLLFEEYTNIPISLLFNEKEADDNDHEPQISPGGLEPPSEKHFRETEV

**HAM1**	NLHLLFEEYTNIPISLLFNEKEADDNDHEPQISPGGLKPPSEKHFRETEV
**HAM2**	NLHLLFEEYTNIPISLLFNEKEADDNDHEPQISPGGLEPSEKHFRETEV
**HAM3**	NLHLLFEEYTNIPISLLFNEKEADDNDHEPQISPGGLEPSEKHFRETEV
**HAM4**	NLHLLFEEYTNIPISLLFNEKEADDNDHEPQISPGGLEPSEKHFRETEV
**HAM5**	NLHLLFEEYTNIPISLLFNEKEADDNDHEPQISPGGLEPSEKHFRETEV
**HAM6**	NLHLLFEEYTNIPISLLFNEKEADDNDHEPQISPGGLEPSEKHFRETEV
**HAM7**	NLHLLFEEYTNIPISLLFNEKEADDNDHEPQISPGGLEPSEKHFRETEV
**HAM8**	NLHLLFEEYTNIPISLLFNEKEADDNDHEPQISPGGLEPSEKHFRETEV
**HAM9**	NLHLLFEEYTNIPISLLFNEKEADDNDHEPQISPGGLEPSEKHFRETEV
**HAM10**	NLHLLFEEYTNIPISLLFNEKEADDNDHEPQISPGGLEPSEKHFHETEV

**ACH**	NLHLLFEEYTNIPISLLFNEKEADDNEHEPQISPGGLEPSEKHFRETEV
**K30p**	NLHLLFEEYTNIPISLLFNEKEADDNEHEPQISPGGLEPSEKHFRETEV
