# Supplementary material for: Characterization of HTLV-1 Infectious Molecular Clone Isolated from Patient with HAM/TSP and Immortalization of Human Primary T-Cell Lines
Source: Viruses. 2024 Nov 9;16(11):1755. doi: 10.3390/v16111755 (PMC11599126; doi:10.3390/v16111755)
Supplement: Supplementary file 1 [file viruses-16-01755-s001.zip › Supplemental S12 HBZ align.pdf]

HTLV-1 HBZ ALIGNMENTS BETWEEN JAPANESE AND BRAZILIAN HAM/TSP ISOLATES (FULL GENOME SEQUENCE) AND OTHER HTLV-1 MOLECULAR CLONES

**PBST**	MAASGLFRCLPVPCPEDLLVEELVDGLLSLEEELKDKEEEEAVLDGLLSLEEESRGRLRRGPPGEKAPPRGETHRDRQRRAEEKRKRKKEREKEEEKQIAEYL
**HAM1**	MAASGLFRCLPGSCPEDLLVEELVDGLLSLEEELKDKEEEEAVLDGLLSLEEESRGRLRQGPPGEKAPPRGETHRDRQRRAEEKRKRKKEREKEEEKQIAEYL
**HAM2**	MAASGPFRCLPVPCPEDLLVEELVDGLLSLEEELKDKEEEEAVLDGLLSLEEESRGRLRRGPPGEKAPPRGETHRDRQRRAEEKRKRKKEREKEEEKQIAEYL
**HAM3**	MAASGLFRCLPVPCPEDLLVEELVDGLLSLEEELKDKEEEEAVLDGLLSLEEESRGRLRRGPPGEKAPPRGETHRDRQRRAEERRKQKKEREKEEEKQIAEYL
**HAM4**	MAASGLFRCLPVPCPEDLLVEELVDGLLSLEEELKDKEEEEAVLDGLLSLEEESRGRLRRGPPGEKAPPRGETHRDRQRRAEEKRKQKKEREKEEEKQIAEYL
**HAM5**	MAASGLFRCLPVPCPEDLLVEELVDGLLSLEEELKDKEEEEAVLDGLLSLEEESRGRLRRGPPGEKAPPRGETHRDRQRRAEEKRKRKKEREKEEEKQIAEYL
**HAM6**	MAASGLFRCLPASCPEDLLVEELVDGLLSLEEELKDKEEEEAVLDGLLSLEEESRGRLRRGPPGEKAPPRGETHRDRQRRAEEKRKRKKEREKEEEKQIAEYL
**HAM7**	MAASGLFRCLPVPCPEDLLVEELVDGLLSLEEELKDKEEEEAVLDGLLSLEEESRGRLRRGPPGEKAPPRGETHRDRQRRAEEKRKRKKEREKEEEKQIAEYL
**HAM8**	MAASGLFRCLPVPCPEDLLVEELVDGLLSLEEELKDKEEEEAVLDGLLSLEEESRGRLRRGPPGEKAPPRGETHRDRQRRAEEKRKRKKEREKEEEKQIAEYL
**HAM9**	MAASGLFRCLPVMCPEDLLVEELVDGLLSLEEELKDKEEEEAVLDGLLSLEEESRGRLRRGPPGEKAPPRGETHRDRQRRAEEKRKRKKEREKEEEKQIAEYL
**HAM10**	MAASGLFRCLPVPCPEDLLVEELVDGLLSLEEELKDKEEEEAVLDGLLSLEEESRGRLRRGPPGEKAPPRGETHRERQRRAEEKRKRKKEREKEEEKQIAEYL
**HAM11**	MAASGLFRCLPVPCPEDLLVEELVDGLLSLEEELKDKEEEEAVLDGLLSLEEESRGRLRRGPPGEKAPPRGETHRDRQRRAEEKRKRKKEREKEEEKQIAEYL
**HAM12**	MAASGLFRCLPVMCPEDLLVEELVDGLLSLEEELKDKEEEEAVLDGLLSLEEESRGRLRRGPPGEKAPPRGETHRDRQRRAEEKRKRKKEREKEEEKQIAEYL
**HAM13**	MAASGLFRCLPVPCPEDLLVEELVDGLLSLEEELKDKEEEEAVLDGLLSLEEESRGRLRRGPPGEKAPPRGETHRDRQRRAEEKRKRKKEREKEEEKQIAEYL
**HAM14**	MAASGLFRCLPVPCPEDLLVEELVDGLLSLEEELKDKEEEEAVLDGLLSLEEESRGRLRRGPPGEKAPPRGETHRDRQRRAEEKRKRKKEREKEEEKQIAEYL
**HAM15**	MAASGLFRCLPVPCPEDLLVEELVDGLLSLEEELKGKEEEEAVLDGLLSLEEESRGRLRRGPPGEKAPPRGETHRDRQRRAEEKRKRKKEREKEEEKQIAEYL
**HAM16**	MAASGLFRCLPVPCPEDLLVEELVDGLLSLEEELKDKEEEEAVLDGLLSLEEESRGRLRRGPPGEKAPPRGETHRDRQRRAEEKRKRKKEREKEEEKQIAEYL
**HAM17**	MAASGLFRCLPVPCPEDLLVEELVDGLLSLEEELKDKEEEEAVLDGLLSLEEESRGRLRRGPPGEKAPPRGETHRDRQRRAEEKRKRKKEREKEEEKQIAEYL
**HAM18**	MAASGLFRCLPVPCPEDLLVEELVDGLLSLEEELKDKEEEEAVLDGLLSLEEESRGRLRRGPPGEKAPPRGETHRDRQRRAEEKRKRKKEREKEEEKQIAEYL
**HAM19**	MAASGLFRCLPVPCPEDLLVEELVDGLLSLEEELKDKEEEEAVLDGLLSLEEESRGRLRRGPPGEKAPPRGETHRDRQRRAEEKRKRKKEREKEEEKQIAEYL
**HAM20**	MAASGLFRCLPVPCPEDLLVEELVDGLLSLEEELKDKEEEEAVLDGLLSLEEESRGRLRRGPPGEKAPPRGETHRDRQRRAEEKRKRKKEREKEEEKQIAEYL
**HAM21**	MAASGLFRCLPVPCPEDLLVEELVDGLLSLEEELKDKEEEEAVLDGLLSLEEESRGRLRRGPPGEKAPPRGETHRDRQRRAEEKRKRKKEREKEEEKQIAEYL
**HAM22**	MAASGLFRCLPVPCPEDLLVEELVDGLLSLEEELKDKEEEEAVLDGLLSLEEESRGRLRRGPPGEKAPPRGETHRDRQRRAEEKRKRKKEREKEEEKQIAEYL
**HAM23**	MAASGLFRCLPVPCPEDLLVEELVDGLLSLEEELKDKEEEEAVLDGLLSLEEESRGRLRRGPPGEKAPPRGETHRDRQRRAEEKRKRKKEREKEEEKQIAEYL
**HAM24**	MAASGLFRCLPVPCPEDLLVEELVDGLLSLEEELKDKEEEEAVLDGLLSLEEESRGRLRRGPPGEKAPPRGETHRDRQRRAEEKRKRKKEREKEEEKQTAEYL
**ACH**	MAASGLFRCLPVPCPEDLLVEELVDGLLSLEEELKDKEEEEAVLDGLLSLEEESRGRLRRGPPGEKAPPRGETHRDRQRRAEEKRKRKKEREKEEEKQIAEYL
**K30p**	MAASGLFRCLPVPCPEDLLVEELVDGLLSLEEELKDKEEEEAVLDGLLSLEEESRGRLRRGPPGEKAPPRGETHRDRQRRAEEKRKRKKEREKEEEKQIAEYL
**ATL1**	MAASGLPRCLPVSCPEDLLVEELVDGLLSLEEELKDKEEEEAVLDGLLSLWEESRGRLRRGPPGEKAPPRGETHRDRQRRAEEKRKRKKEREKEEEKQIAEYL
**ATL2**	MAASGLPRCLPVSCPEDLLVEELVDGLLSLEEELKDKEEEEAVLDGLLSLEEESRGRLRRGPPGEKAPPRGETHRDRQRRAEEKRKRKKEREKEEEKQIAEYL
**ATL3**	MAASGLPRCLPVSCPEDLLVEELVDGLLSLEEELKDKEEEEAVLDGLLSLEEESRGRLRRGPPGEKAPPRGETHRDRQRRAEEKRKRKKEREKEEEKQIAEYL
**ATL4**	MAASGLFRCLPVSCPEDLLVEELVDGLLSLEEELKDKEEEEAVLDGLLSLEEESRGRLRRGPPGEKAPPRGETHRDRQRRAEEKRKRKKEREKEEEKQIAEYL
**ATL5**	MAASGLFRCLPVSCPEDLLVEELVDGLLSLEEELKDKEEEEAVLDGLLSLEEESRGRLRRGPPGEKAPPRGETHRDRQRRAEEKRKRKKEREKEEEKQIAEYL
**ATL6**	MAASGLPRCLPVSCPEDLLVEELVDGLLSLEEELKDKEEEEAVLDGLLSLEEESRGRLRRGPPGEKAPPRGETHRDRQRRAEEKRKRKKEREKEEEKQIAEYL
**ATL7**	MAASGLFRCLPVSCPEDLLVEELVDGLLSLEEELKDKEEEEAVLDGLLSLEEESRGRLRRGPPGEKAPPRGETHRDRQRRAEEKRKRKKEREKEEEKQIAEYL
**ATL8**	MAASGLFRCLPVSCPEDLLVEELVDGLLSLEEELKDKEEEEAVLDGLLSLEEESRGRLRRGPPGEKAPPRGETHRDRQRRAEEKRKRKKEREKEEEKQIAEYL
**ATL9**	MAASGLPRCLPVSCPEDLLVEELVDGLLSLEEELKDKEEEEAVLDGLLSLEEESRGRLRRGPPGEKAPPRGETHRDRQRRAEEKRKRKKEREKEEEKQIAEYL
**ATL10**	MAASGLFRCLPVSCPEDLLVEELVDGLLSLEEELKDKEEEEAVLDGLLSLEEESRGRLRRGPPGEKAPPRGETHRDRQRRAEEKRKRKKEREKEEEKQIAEYL
**ATL11**	MAASGLPRCLPVSCPEDLLVEELVDGLLSLEQELKDKEEEEAVLDGLLSLEEESRGRLRRGPPGEKAPPRGETHRDRQRRAEEKRKRKKEREKEEEKQIAEYL
**ATL12**	MAASGLMPCLPVSCPEDLLVEELVDGLLSLEEQLKDKEEEEAVLDGLLSLEEESRGRLRRGPPGEKAPPRGETHRDRQRRAEEKRKRKKEREKEEEKQIAEYL
**ATL13**	MAASGLFRCLPVSCPEDLLVEELVDGLLSLEEELKDKEEEEAVLDGLLSLEEESRGRLRRGPPGEKAPPRGETHRDRQRRAEEKRKRKKEREKEEEKQIAEYL
**ATL14**	MAASGLPRCLPVSCPEDLLVEELVDGLLSLEEELKDKEEEEAVLDGLLSLEEESRGRLRRGPPGEKAPPRGETHRDRQRRAEEKRKRKKEREKEEEKQIAEYL
**ATL15**	MAASGLFRCLPVSCPEDLLVEELVDGLLSLEEELKDKEEEEAVLDGLLSLEEESRGRLRRGPPGEKAPPRGETHRDRQRRAEEKRKRKKEREKEEEKQIAEYL
**ATL16**	MAASGLFRCLPVSCPEDLLVEELVDGLLSLEEELKDKEEEEAVLDGLLSLEEESRGRLRRGPPGEKAPPRGETHRDRQRRAEEKRKRKKEREKEEEKQIAEYL
**ATL17**	MAASGLFRCLPVSCPEDLLVEELVDGLLSLEEELKDKEEEEAVLDGLLSLEEESRGRLRRGPPGEKAPPRGETHRDRQRRAEEKRKRKKEREKEEEKQIAEYL
**ATL18**	MAASGLFRCLPVSCPEDLLVEELVDGLLSLEEELKDKEEEEAVLDGLLSLEEESRGRLRRGPPGEEAPPRGETHRDRQRRAEEKRKRKKEREKEEEKQIAEYL
**ATL19**	MAASGLPRCLPVSCPEDLLVEELVDGLLSLEEELKDKEEEEAVLDGLLSLEEESRGRLRRGPPGEKAPPRGETHRDRQRRAEEKRKRKKEREKEEEKQIAEYL
**ATL20**	MAASGLPRCLPVSCPEDLLVEELVDGLLSLEEELKDKEEEEAVLDGLLSLEEESRGRLRRGPPGEKAPPRGETHRDRQRRAEEKRKRKKEREKEEEKQIAEYL

**PBST**	KRKEEEKARRRRQAEKKAADVARRKQEEQERRERKWRQGAEKAKQHSARKEKMQELGIDGYTRQLEGEVESLEAERRKLLQEKEDLMGEVNYWQGRLEAMWLQ
**HAM1**	KRKEEEKARRRRRAEKKAADVARRKQEEQERRERKWRQGAEKAKQHSARKEKMQELGIDGYTRQLEGEVESLEAERRKLLQEKEDLMGEVNYWQGRLEAMWLQ
**HAM2**	KRKEEEKARRRRRAEKKAADVARRKQEEQERRERKWRQGAEKAKQHSARKEKMQELGIDGYTRQLEGEVESLEAERRKLLQEKEDLMGEVNYWQGRLEAMWLQ
**HAM3**	KRKEEEKARRRRRAEKKAADVARRKQEEQERRERKWRQGAEKAKQHSARKEKMQELGIDGYTRQLEGEVESLEAERRKLLQEKEDLMGEVNYWQGRLEAMWLQ
**HAM4**	KRKEEEKARRRRRAEKKAADVARRKQEEQERRERKWRQGAEKAKQHSARKEKMQELGIDGYTRQLEGEVESLEAERRKLLQEKEDLMGEVNYWQGRLEAMWLQ
**HAM5**	KRKEEEKARRRRRAEKKAADVARRKQEEQERRERKWRQGAEKAKQHSARKEKMQELGIDGYTRQLEGEVESLEAERRKLLQEKEDLMGEVNYWQGRLEAMWLQ
**HAM6**	KRKEEEKARRRRRAEKKAADVARRKQEEQERRERKWRQGAEKAKQHSARKEKMQELGIDGYTRQLEGEVESLEAERRKLLQEKEDLMGEVNYWQGRLEAMWLQ
**HAM7**	KRKEEEKARRRRRAEKKAADVARRKQEEQERRERKWRQGAEKAKQHSARKEKMQELGIDGYTRQLEGEVESLEAERRKLLQEKEDLMGEVNYWQGRLEAMWLQ
**HAM8**	KRKEEEKARRRRRAEKKAADVARRKQEEQERRERKWRQGAEKAKQHSARKEKMQELGIDGYTRQLEGEVESLEAERRKLLQEKEDLMGEVNYWQGRLEAMWLQ
**HAM9**	KRKEEEKARRRRRAEKKAADVARRKQEEQERRERKWRQGAEKAKQHSARKEKMQELGIDGYTRQLEGEVESLEAERRKLLQEKEDLMGEVNYWQGRLEAMWLQ
**HAM10**	KRKEEEKARRRRRAEKKAADVARRKQEEQERRERKWRQGAEKAKQHSARKEKMQELGIDGYTRQLEGEVESLEAERRKLLQEKEDLMGEVNYWQGRLEAMWLQ
**HAM11**	KRKEEEKARRRRRAEKRAADVARRKQEEQERRERKWRQGAEKAKQHSARKEKMQELGIDGYTRQLEGEVESLEAERRKLLQEKEDLMGEVNYWQGRLEAMWLQ
**HAM12**	KRKEEEKARRRRRAEKKAADVARRKQEEQERRERKWRQGAEKAKQHSARKEKMQELGIDGYTRQLEGEVESLEAERRKLLQEKEDLMGEVNYWQGRLEAMWLQ
**HAM13**	KRKEEEKARRRRQAEKKAADVARRKQEEQERRERKWRQGAEKAKQHSARKEKMQELGIDGYTRQLEGEVESLEAERRKLLQEKEDLMGEVNYWQGRLEAMWLQ
**HAM14**	KRKEEEKARRRRRAEKKAADVARRKQEEQERRERKWRQGAEKAKQQSARKEKMQELGIDGYTRQLEGEVESLEAERRKLLQEKEDLMGEVNYWQGRLEAMWLQ
**HAM15**	KRKEEEKARRRRRAEKKAADVARRKQEEQERRERKWRQGAEKAKQHSARKEKMQELGIDGYTRQLEGEVESLEAERRKLLQEKEDLMGEVNYWQGRLEAMWLQ
**HAM16**	KRKEEEKARRRRRAEKKAADVARRKQEEQERRERKWRQGAEKAKQHSARKEKMQELGIDGYTRQLEGEVESLEAERRKLLQEKEDLMGEVNYWQGRLEAMWLQ
**HAM17**	KRKEEEKARRRRQAEKKAADVARRKQEEQERRERKWRQGAEKAKQHSARKEKMQELGIDGYTRQLEGEVESLEAERRKLLQEKEDLMGEVNYWQGRLEAMWLQ
**HAM18**	KRKEEEKARRRRQAEKKAADVAGRKQEEQERRERKWRQGAEKAKQHSARKEKMQELGIDGYTRQLEGEVESLEAERRKLLQEKEDLMGEVNYWQGRLEAMWLQ
**HAM19**	KRKEEEKARRRRQAEKKAADVARRKQEEQERRERKWRQGAEKAKQHSARKEKMQELGIDGYTRQLEGEVESLEDERRKLLQEKEDLMGEVNYWQGRLEAMWLQ
**HAM20**	KRKEEEKARRRRRAEKKAADVARRKQEEQERRERKWRQGAEKAKQHSARKEKMQELGIDGYTRQLEGEVESLEAERRKLLQEKEDLMGEVNYWQGRLEAMWLQ
**HAM21**	KRKEEEKARRRRQAEKKAADVARRKQEEQERRERKWRQGAEKAKQHSARKEKMQELGIDGYTRQLEGEVESLEAERRKLLQEKEDLMGEVNYWQGRLEAMWLQ
**HAM22**	KRKEEEKARRRRQAEKKAADVARRKQEEQERRERKWRQGAEKAKQHSARKEKMQELGIDGYTRQLEGEVESLEAERRKLLQEKEDLMGEVNYWQGRLEAMWLQ
**HAM23**	KRKEEEKARRRRRAEKKAADVARRKQEEQERRERKWRQGAEKAKQHSARKEKMQELGIDGYTRQLEGEVESLEAERRKLLQEKEDLMGEVNYWQGRLEAMWLQ
**HAM24**	KRKEEEKARRRRRAEKKAADVARRKQEEQERRERKWRQGAEKAKQHSARKEKMQELGIDGYTRQLEGEVESLEAERRKLLQEKEDLMGEVNYWQGRLEAMWLQ
**ACH**	KRKEEEKARRRRQAEKKAADVARRKQEEQERRERKWRQGAEKAKQHSARKEKMQELGIDGYTRQLEGEVESLEAERRKLLQEKEDLMGEVNYWQGRLEAMWLQ
**K30p**	KRKEEEKARRRRQAEKKAADVARRKQEEQERRERKWRQGAEKAKQHSARKEKMQELGIDGYTRQLEGEVESLEAERRKLLQEKEDLMGEVNYWQGRLEAMWLQ
**ATL1**	KRKEEEKARRRRRAEKKAADVARRKQEEQERRERKWRQGAEKAKQHSARKEKMQELGIDGYTRQLEGEVESLEAERRKLLQEKEDLMGEVNYWQGRLEAMWLQ
**ATL2**	KRKEEEKARRRRRAEKKAADVARRKQEEQERRERKWRQGAEKAKQHSARKEKMQELGIDGYTRQLEGEVESLEAERRKLLQEKEDLMGEVNYWQGRLEAMWLQ
**ATL3**	KRKEEEKARRRRRAEKKAADVARRKQEEQERRERKWRQGAEKAKQHSARKEKMQELGIDGYTRQLEGEVESLEAERRKLLQEKEDLMGEVNYWQGRLEAMWLQ
**ATL4**	KRKEEEKARRRRRAEKKAADVARRKQEEQERRERKWRQGADKAKQHSAQKEKMQELGIDGYTRQLEGEVESLEAERRKLLQEKEDLMGEVNYWQGRLEAMWLQ
**ATL5**	KRKEEEKARRRRRAEKKAADVARRKQEEQERRERKWRQGADKAKQHSAQKEKMQELGIDGYTRQLEGEVESLEAERRKLLQEKEDLMGEVNYWQGRLEAMWLQ
**ATL6**	KRKEEEKARRRRRAEKKAADVARRKQEEQERRERKWRQGAEKAKQHSAVKEKMQELGIDGYTRQLEGEVESLEAERRKLLQEKEDLMGEVNYWQGRLEAMWLQ
**ATL7**	KRKEEEKARRRRRAEKKAADVARRKQEEQERRERKWRQGAEKAKQHSARKEKMQELGIDGYTRQLEGEVESLEAERRKLLQEKEDLMGEVNYWQGRLEAMWLQ
**ATL8**	KRKEEEKARRRRRAEKKAADVARRKQEEQERRERKWRQGAEKAKQHSARKEKMQELGIDGYTRQLEGEVESLEAERRKLLQEKEDLMGEVNYWQGRLEAMWLQ
**ATL9**	KRKTEEKARRRRRAEKKAADVARRKQEEQERRERKWRQGAEKAKQHSARKEKMQELGIDGYTRQLEGEVESLEAERRKLLQEKEDLMGEVNYWQGRLEAMWLQ
**ATL10**	KRKEEEKARRRRRAEKKAADVARRKQEEQERRERKWRQGADKAKQHSAQKEKMQELGIDGYTRQLEGEVESLEAERRKLLQEKEDLMGEVNYWQGRLEAMWLQ
**ATL11**	KRKEEEKARRRRRAEKKAADVARRKQEEQERRERKWRQGAEKAKQHSARKEKMQELGIDGYTRQLEGEVESLEAERRKLLQEKEDLMGEVNYWQGRLEAMWLQ
**ATL12**	KRKEEEKARRRRRAEKKAADVARRKQEEQERRERKWRQGAEKAKQHSARKEKMQELGIDGYTRQLEGEVESLEAERRKLLQEKEDLMGEVNYWQGRLEAMWLQ
**ATL13**	KRKEEEKARRRRRAEKKAADVARRKQEEQERRERKWRQGADKAKQHSAQKEKMQELGIDGYTRQLEGEVESLEAERRKLLQEKEDLMGEVNYWQGRLEAMWLQ
**ATL14**	KRKEEEKARRRRRAEKKAADVARRKQEEQERRERKWRQGAEKAKQHSARKEKMQELGIDGYTRQLEGEVESLEAERRKLLQEKEDLMGEVNYWQGRLEAMWLQ
**ATL15**	RRKEEEKARRRRRAEKKAADVARRKQEEQERRERKWRQGAEKAKQHSARKEKMQELGIDGYTRQLEGEVESLEAERRKLLQEKEDLMGEVNYWQGRLEAMWLQ
**ATL16**	KRKEEEKARRRRRAEKKAADVARRKVEEQERRERKWRQGADKAKQHSAQKEKMQELGIDGYTRQLEGEVESLEAERRKLLQEKEDLMGEVNYWQGRLEAMWLQ
**ATL17**	KRKEEEKARRRRRAEKKAADVARRKQEEQERRERKWRQGADKAKQHSAQKEKMQELGIDGYTRQLEGEVESLEAERRKLLQEKEDLMGEVNYWQGRLEAMWLQ
**ATL18**	KRKEEEKARRRRRAEKKAADVARRKQEEQERRERKWRQGAEKAKQHSARKEKMQELGIDGYTRQLEGEVESLEAERRKLLQDKEDLMGEVNYWQGRLEAMWLQ
**ATL19**	KRKEEEKARRRRRAEKKAADVARRKQEEQERRERKWRQGAEKAKQHSARKEKMQELGIDGYTRQLEGEVESLEAERRKLLQEKEDLMGEVNYWQGRLEAMWLQ
**ATL20**	KRKEEEKARRRRRAEKKAADVARRKQEEQERRERKWRQGAEKAKQHSARKEKMQELGIDGYTRQLEGEVESLEAERRKLLQEKEDLMGEVNYWQGRLEAMWLQ
