# Supplementary material for: Characterization of HTLV-1 Infectious Molecular Clone Isolated from Patient with HAM/TSP and Immortalization of Human Primary T-Cell Lines
Source: Viruses. 2024 Nov 9;16(11):1755. doi: 10.3390/v16111755 (PMC11599126; doi:10.3390/v16111755)
Supplement: Supplementary file 1 [file viruses-16-01755-s001.zip › Supplemental S2 pAB SEQ.pdf]

PAB MOLECULAR CLONE

(1)GGGGGCTTAGAGCCTCCCAGTGAAAAACATTTCCGCGAAACAGAAGTCTGAAAAGGTCAGGGCCCAGACTAAGGCTC
TGACGTCTCCCCCCGGAGGGACAGCTCAGCACCGGCTCAGGCTAGGCCCTGACGTGTCCCCCTGAAGACAAATCATAAGC
TCAGACCTCCGGGAAGCCACCGGAACCACCCATTTCCTCCCCATGTTTGTCAAGCCGCCCTCAGGCGTTGACGACAACCCC
TCACCTCAAAAAACTTTTCATGGCACGCATATGGCTGAATAAACTAACAGGAGTCTATAAAAGCGTGGAGACAGTTCAGG
AGGGGGCTCGCATCTTTCCTTCACGCGCCCGCCGCCCTACCTGAGGCCGCCATCCACGCCGGTTGAGTCGCGTTCTGCCGC
CTCCCGCCTGTGGTGCCTCCTGAACTGCGTCCGCCGTCTAGGTAAGTTTAGAGCTCAGGTCGAGACCGGGCCTTTGTCCG
GCGCTCCCTTGGAGCCTACCTAGACTCAGCCGGCTCTCCACGCTTTGCCTGACCCTGCTTGCCCAACTCTGCGTCTTTGTTT
CGTTTTCTGTTCTGCGCCGCTACAGATCGAAAGTTCCACCCCTTTCCCTTTCATTCACGACTGACTGCCGGCTTGGCCCACG
GCCAAGTACCGGCGACTCCGTTGGCTCGGAGCCAGCGACAGCCCATTCTATAGCACTCTCCAGGAGAGAAATTTAGTACA
CAAGAGGGGGCTCACTCGGGGATAGAGGGGCTGGGCATGGCGGTAGGCAATGGGCCAAATCTTTTCCCGTAACGCTAG
CCCTATTCCGCGGCCGCCCCGGGGGCTGGCCGCTCATCACTGGCTTAACTTCCTCCAAGCGGCATATCGCCTAGAACCCGG
TCCCTCCAGTTACGATTTCCACCAGTTAAAAAAATTTCTTAAAATAGCTTTAGAAACACCGGTCTGGATCTGTCCCATTAAC
TACTCCCTCCTAGCCAGTCTACTCCCAAAAGGATACCCCGGCCGGGTGAATGAAATTTTACACATACTCATCCAAACCCAA
GCCCAGATCCCGTCCCGTCCCGCGCCACCGCCGCCGTCATCCCCCACCCACGACCCCCCGGATTCTGATCCACAAATCCCCC
CTCCCTATGTTGAGCCTACGGCCCCCCAAGTCCTTCCAGTCATGCACCCACATGGTGCCCCTCCCAACCATCGCCCATGGCA
AATGAAAGACCTACAGGCCATTAAGCAAGAAGTCTCCCAAGCAGCCCCTGGGAGCCCCCAGTTTATGCAGACCATCCGGC
TTGCGGTGCAGCAGTTTGACCCCACTGCCAAAGACCTCCAAGACCTCCTGCAGTACCTTTGCTCCTCCCTCGTGGCTTCCCT
CCATCACCAGCAGCTAGATAGCCTTATATCAGAGGCCGAAACCCGAGGTATTACAGGTTATAACCCCTTAGCCGGTCCCCT
CCGTGTCCAAGCCAACAATCCACAACAACAAGGATTAAGGCGAGAATACCAGCAACTCTGGCTCGCCGCCTTCGCCGCCC
TGCCAGGGAGTGCCAAAGACCCTTCCTGGGCCTCTATCCTCCAAGGCCTGGAGGAGCCTTACCACGCCTTCGTAGAACGC
CTCAACATAGCTCTTGACAATGGGCTGCCAGAAGGCACGCCCAAAGACCCCATCTTACGTTCCTTAGCCTACTCCAATGCA
AACAAAGAATGCCAAAAATTACTACAGGCCCGAGGACACACTAATAGCCCTCTAGGAGATATGTTGCGGGCTTGTCAGAC
CTGGACCCCCAAAGACAAAACCAAAGTGTTAGTTGTCCAGCCTAAAAAACCCCCCCAAATCAGCCGTGCTTCCGGTGCG
GGAAAGCAGGCCACTGGAGTCAGGACTGCACTCAGCCTCGTCCCCCCCCCGGGCCATGCCCCCTATGTCAAGACCCAACT
CACTGGAAGCGAGACTGCCCCCGCCTAAAGCCCACTATCCCAGAACCAGAGCCAGAGGAAGATGCCCTCCTATTAGACCT
CCCCGCCGACATCCCACACCCAAAAAACTCCATAGGGGGGGAGGTTTAACCTCCCCCCCACATTACAACAAGTCCTTCCT
AACCAAGACCCAGCATCTATTCTGCCAGTTATACCGTTAGATCCCGCCCGTCGGCCCGTAATTAAAGCCCAGGTTGACACC
CAGACCAGCCACCCAAAGACTATCGAAGCTTTACTAGATACAGGAGCAGACATGACAGTCCTTCCGATAGCCTTGTTCTCA
AGTAATACTCCCCTCAAAAATACATCCGTATTAGGGGCAGGAGGCCAAACCCAAGATCACTTTAAGCTCACCTCCCTTCCT
GTGCTAATACGCCTCCCTTTCCGGACAACGCCTATTGTTTTAACATCTTGCCTAGTTGATACCAAAAACAACTGGGCCATCA
TAGGTCGTGATGCCTTACAACAATGCCAAGGCGTCCTGTACCTCCCTGAGGCAAAAAGGCCGCCTGTAATCTTGCCAATAC
AGGCGCCAGCCGTCCTTGGGCTAGAACACCTCCCAAGGCCCCCCGAAATCAGCCAGTTCCCTTTAAACCAGAACGCCTCCA
GGCCTTGCAACACTTGGTCCGGAAGGCCCTGGAGGCAGGCCATATCGAACCCTACACCGGGCCAGGAAATAACCCAGTA
TTCCCAGTTAAAAAGGCCAATGGAACCTGGCGATTCATCCACGACCTGCGGGCCACTAACTCTCTAACCATAGATCTCTCA
TCATCTTCCCCCGGGCCCCCTGACTTGTCCAGCCTGCCAACTACACTAGCCCACTTGCAAACTATAGACCTTAAAGACGCCT
TTTTCCAAATCCCCTTACCTAAACAGTTCCAGCCCTACTTTGCTTTCACTGTCCCACAGCAGTGTAACTACGGCCCCGGCACT
AGATACGCCTGGAAAGTACTACCCCAAGGGTTTAAAAATAGTCCCACCCTGTTCGAAATGCAGCTGGCTCATATCCTGCAG
CCCATTCGGCAAGCTTTCCCCCAATGCACTATTCTTCAGTACATGGATGACATTCTCCTGGCAAGCCCCTCCCATGAGGACC
TACTACTACTCTCAGAGGCCACAATGGCTTCCCTAATCTCCCATGGGTTGCCTGTGTCCGAAAACAAAACCCAGCAAACCC
CTGGAACAATTAAGTTCCTAGGGCAAATAATTTCACCTAATCACCTCACTTATGATGCAGTCCCCACGGTACCTATACGGTC
CCGCTGGGCGCTACCTGAACTTCAAGCCCTACTTGGCGAGATTCAGTGGGTCTCCAAGGGAACTCCTACCTTACGCCAGCC
CCTTCACAGTCTCTACTGTGCCTTACAAAGGCATACTGATCCCCGAGACCAAATATATTTAAATCCTTCTCAAGTTCAATCAT
TAGTGCAGCTGCGGCAGGCCCTGTCACAGAACTGCCGCAGTAGACTAGTCCAAACCCTGCCCCTCCTAGGGGCTATTATG
CTGACCCTCACTGGCACCACTACTGTAGTGTTCCAGTCCAAGCAGCAGTGGCCACTTGTCTGGCTACATGCCCCCCTACCCC

ACACTAGCCAGTGCCCCTGGGGGCAGCTACTTGCCTCAGCTGTGTTATTACTCGACAAATACACCTTGCAATCCTATGGGC
TACTCTGCCAAACCATACATCATAACATCTCCACCCAAACCTTCAACCAATTCATTCAAACATCTGACCACCCCAGTGTTCCT
ATCTTACTCCATCACAGTCACCGATTCAAAAATTTATGTGCCCAAACTGGAGAACTTTGGAACACTTTTCTTAAAACAGCTG
CCCCATTGGCTCCTGTGAAAGCCCTCATGCCAGTGTTTACTCTTTCCCCGGTGATCCTAAACACCGCCCCCTGCCTGTTTTCA
GACGGATCTACCTCCCGGGCAGCCTATATTCTCTGGGACAAGCATATATTGTCACAAAGATCATTCCCCCTTCCGCCACCG
CACAAGTCGGCCCAACGGGCCGAACTTCTCGGACTTTTGCATGGCCTCTCCAGCGCCCGTTCGTGGCGCTGTCTCAACATA
TTTCTAGACTCCAAGTATCTTTATCATTACCTTCGGACCCTTGCCCTAGGCACCTTCCAAGGCAAGTCATCTCAGGCCCCCTT
TCAGGCCCTCCTGCCCCGCTTACTATCGCGTAAGGTCGTCTATTTGCACCACGTTCGCAGCCATACCAATCTACCTGATCCC
ATCTCCAGGCTCAACGCTCTCACAGATGCCCTACTAATCACCCCTGTCCTGCAGCTCTCTCCTGCAGAACTACACAGTTTCA
CCCATTGCGGACAGACGGCCCTCACATTGCAAGGGGCAACCACAACTGAGGCTTCCAATATCCTGCGCTCTTGCCACGCCT
GCCGCAAAAATAACCCACAACATCAGATGCCTCGGGGACACATCCGCCGTGGCCTACTTCCTAACCACATCTGGCAAGGC
GACATTACCCATTTCAAATATAAAAATACGCTGTATCGCCTTCATGTATGGGTAGACACCTTTTCAGGAGCCATCTCAGCTA
CCCAAAAGAGAAAAGAAACAAGCTCAGAAGCTATTTCCTCTTTGCTTCAGGCCATTGCCTATCTAGGCAAGCCTAGCTACA
TAAACACAGACAACGGCCCTGCCTATATTTCCCAAGACTTCCTCAATATGTGTACCTCCCTTGCTATTCGCCATACTACCCAT
GTCCCCTACAATCCAACCAGCTCAGGACTCGTAGAACGCTCTAATGGCATTCTTAAAACCCTACTATATAAGTACTTTACTG
ACAAACCCGACCTACCCATGGATAATGCTCTATCCATAGCCCTATGGACAATCAACCACCTGAATGTGTTAACCAACTGCC
ACAAAACCCGATGGCAGCTTCACCACTCCCCCCGACTCCAGCCGATCCCAGAGACACATTCCCTCAGCAATAAACAAACCC
ATTGGTATTATTTCAAGCTTCCTGGTCTTAATAGCCGCCAGTGGAAAGGACCACAGGAGGCTCTCCAAGAAGCTGCCGGC
GCTGCTCTCATCCCGGTAAGCGCTAGTTCTGCCCAGTGGATCCCGTGGAGACTCCTCAAGCGAGCTGC*ATGCCCAAGACCC*
*GTCGGAGGCCCCGCCGATCCCAAAGAAAAAGACCACCAACACC*ATGGGTAAGTTTCTCGCCACTTTAATTTTATTCTTCCA
GTTCTGCCCCCTCATCCTCGGTGATTACAGCCCCAGCTGCTGTACTCTCACAATTGGAGTCTCCTCATACCACTCTGAACCCT
GCAATCCTGCCCAGCCAGTTTGTTCGTGGACCCTCGACCTGCTGGCCCTTTCAGCAGATCAGGCCCTACAGCCCCCCTGCC
CTAATCTAGTAGGTTACTCTAGCTACCATGCCACCTATTCCCTATATCTATTCCCTCATTGGATTAAAAAGCCAAACCGAAA
TGGCGGAGGCTATTATTCAGCCTCTTATTCAGACCCTTGTTCCTTAAAGTGCCCATACCTGGGGTGCCAATCATGGACCTG
CCCCTATACAGGAGCCGTCTCCAGCCCCTACTGGAAGTTTCAGCAAGATGTCAATTTTACTCAAGAAGTTTCACGCCTCAAT
ATTAATCTCCATTTTTCGAAATGCGGTTTTCCCTTCTCCCTTCTAGTCGACctccaggatatgaccccatctggttccttaataccgaaccca
gccaactgcctcccaccgcccctcctctactcccccactctaacctagaccacatcctcgagccctctataccatggaaatcaaaactcctgacccttgtccagtta
accctacaaagcactaattatacttgcattgtctgtatcgatcgtgccagcctatccacttggcacgtcctatactctcccaacgtctctgttccatcctcttcttcta
cccccctcctttacccatcgttagcgcttccagccccccacctgacgttaccatttaactggacccactgctttgacccccagattcaagctatagtctcctccccct
gtcataactccctcatcctgcccccctttttccttgtcacctgttcccaccctaggatcccgctcccgccgagcggtaccggtggcggtctggcttgtctccgccctgg
ccatgggagccggagtggctggcgggattaccggctccatgtccctcgcctcaggaaagagcctcctacatgaggtggacaaagatatttcccaattaactcaa
gcaatagtcaaaaaccacaaaaatctactcaaaattgcgcagtatgctgcccagaacagacgaggccttgatctcctgttctgggagcaaggaggattatgca
aagcattacaagaacagtgctgttttctgaatattactaattcccatgtctcaatactacaagaaagaccccccccttgagaatcgagtcctgactggctggggcct
taactgggaccttggcctctcacagtgggctcgagaggccttacaaactggaatcacccttgtcgcgctactccttcttgttatccttgcaggaccatgcatcctcc
gtcagctacgacacctcccctcgcgcgtcagatacccccattactctcttataaaccctgagtcatccctgtaaaccaagcacacaattattgcaaccacatcgcc
tccagcctcccctgccaataattaacctctcccattaaatcctccttctcctgcagcaacttcctccgttcagcctccaaggactccacctcgccttccaactgtcta
gtatagccatcaatccccaactcctgcatttttttctttcctagcactatgctgtttcgccttctcagccccttgtctccacttgcgctcacggcgctcctgctcttcctgc
tttctccgggcgacgtcagcagccttcttctccgcctgcctcctgcgccgtgccttctcctcttccttccttttcaaatactcagcaatctgcttttcctcctctttctccc
gctcttttttttcgcttcctcttctcctcagcccgtcgctgccgatcacgatgcgtttccccgcgaggtggcgctttctcccctggagggccccgtcgcagccggccgc
ggctttcctcttctaaggatagcaaaccgtcaagcacagcttcctcctcctccttgtcctttaactcttcctccaaagataatagcccgtccaccaattcctccacca
gcaggtcctccgggcatggcacaggcaagcatcgaaacagccctacagatacaaagttaaccatgcttattatcagcccacttcccagggtttggacagagtct
tcttttcggatacccagtctacgtgtttggagactgtgtacaaggcgactggtgccccatctctgggggactatgttcggcccgcctacatcgtcacgccctactgg
ccacctgtccagagcatcagatcacctgggaccccATCGATGGACGCGTTATCGGCTCAGCTCTACAGTTCCTTATCCCTCGACTCCCCT
CCTTCCCCACCCAGAGAACCTCTAAGACCCTCAAGGTCCTTACCCCGCCAATCACTCATACAACCCCCAACATTCCACCCTC
CTTCCTCCAGGCCATGCGCAAATACTCCCCCTTCCGAAATGGATACATGGAACCCACCCTTGGGCAGCACCTCCCAACCCT
GTCTTTTCCAGACCCCGGACTCCGGCCCCAAAACCTGTACACCCTCTGGGGAGGCTCCGTTGTCTGCATGTACCTCTACCA

GCTTTCCCCCCCCATCACCTGGCCCCTCCTGCCCCACGTGATTTTTTGCCACCCCGGCCAGCTCGGGGCCTTCCTCACCAAT
GTTCCCTACAAGCGAATAGAAGAACTCCTCTATAAAATTTCCCTTACCACAGGGGCCCTAATAATTCTACCCGAAGACTGTT
TGCCCACCACCCTTTTCCAGCCTGTTAGGGCACCCGTCACGCTAACAGCCTGGCAAAACGGCCTCCTTCCGTTCCACTCAAC
CCTCACCACTCCAGGCCTTATTTGGACATTTACCGATGGCACGCCTATGATTTCCGGGCCCTGCCCTAAAGATGGCCAGCC
ATCTTTAGTACTACAGTCCTCCTCCTTTATATTTCACAAATTTCAAACCAAGGCCTACCACCCCTCATTTCTACTCTCACACGG
CCTCATACAGTACTCTTCCTTTCATAATTTACATCTCCTGTTTGAAGAATACACCAACATCCCCATTTCTCTACTTTTTAACGA
AAAAGAGGCAGATGACAATGACCATGAGCCCCAAATATCCCCCGGGGGCTTAGAGCCTCCCAGTGAAAAACATTTCCGCG
AAACAGAAGTCTGAAAAGGTCAGGGCCCAGACTAAGGCTCTGACGTCTCCCCCCCGGAGGGACAGCTCAGCACCGGCTC
AGGCTAGGCCCTGACGTGTCCCCCTGAAGACAAATCATAAGCTCAGACCTCCGGGAAGCCACCGGAACCACCCATTTCCT
CCCCATGTTTGTCAAGCCGCCCTCAGGCGTTGACGACAACCCCTCACCTCAAAAAACTTTTCATGGCACGCATATGGCTGA
ATAAACTAACAGGAGTCTATAAAAGCGTGGAGACAGTTCAGGAGGGGGCTCGCATCTTTCCTTCACGCGCCCGCCGCCCT
ACCTGAGGCCGCCATCCACGCCGGTTGAGTCGCGTTCTGCCGCCTCCCGCCTGTGGTGCCTCCTGAACTGCGTCCGCCGTC
TAGGTAAGTTTAGAGCTCAGGTCGAGACCGGGCCTTTGTCCGGCGCTCCCTTGGAGCCTACCTAGACTCAGCCGGCTCTCC
ACGCTTTGCCTGACCCTGCTTGCCCAACTCTGCGTCTTTGTTTCGTTTTCTGTTCTGCGCCGCTACAGATCGAAAGTTCCACC
CCTTTCCCTTTCATTCACGACTGACTGCCGGCTTGGCCCACGGCCAAGTACCGGCGACTCCGTTGGCTCGGAGCCAGCGAC
AGCCCATTCTATAGCACTCTCCAGGAGAGAAATTTAGTACACA **(9024 - end)**
