# Supplementary material for: Characterization of HTLV-1 Infectious Molecular Clone Isolated from Patient with HAM/TSP and Immortalization of Human Primary T-Cell Lines
Source: Viruses. 2024 Nov 9;16(11):1755. doi: 10.3390/v16111755 (PMC11599126; doi:10.3390/v16111755)
Supplement: Supplementary file 1 [file viruses-16-01755-s001.zip › Supplemental S3 Align pBST ATK.pdf]

Alignment - Top Sequence Seiki ATK HTLV-1A prototype bottom pHTLV-1B. Human adult T-cell leukemia virus: complete nucleotide sequence of the provirus genome integrated in leukemia cell DNA. Seiki et al., Proc Natl Acad Sci U S A 80 (12), 3618-3622 (1983). PUBMED 6304725

TAX (0.85%)

ATK HTLV-1A MAHFPGFGQSLLFGYPVYVFGDCVQGDWCPISGGLCSARLHRHALLATCPEHQITWDPID 60
pHTLV-1B MAHFPGFGQSLLFGYPVYVFRDCVQGDWCPISGGLCSARLHRHALLATCPEHQITWDPID 60

ATK HTLV-1A GRVIGSALQFLIPRLPSFPTQRTSKTLKVLTPPITHTTPNIPPSFLQAMRKYSPFRNGYM 120
pHTLV-1B GRVIGSALQFLIPRLPSFPTQRTSKTLKVLTPPITHTTPNIPPSFLQAMRKYSPFRNGYM 120

ATK HTLV-1A EPTLGQHLPTLSFPDPGLRPQNLYTLWGGSVVCMYLYQLSPPITWPLLPHVIFCHPGQLG 180
pHTLV-1B EPTLGQHLPTLSFPDPGLRPQNLYTLWGGSVVCMYLYQLSPPITWPLLPHVIFCHPGQLG 180

ATK HTLV-1A AFLTNVPYKRIEELLYKISLTTGALIILPEDCLPTTLFQPARAPVTLTAWQNGLLPFHST 240
pHTLV-1B AFLTNVPYKRIEELLYKISLTTGALIILPEDCLPTTLFQPVRAPVTLTAWQNGLLPFHST 240

ATK HTLV-1A LTTPGLIWTFTDGTPMISGPCPKDGQPSLVLQSSSFIFHKFQTKAYHPSFLLSHGLIQYS 300
pHTLV-1B LTTPGLIWTFTDGTPMISGPCPKDGQPSLVLQSSSFIFHKFQTKAYHPSFLLSHGLIQYS 300

ATK HTLV-1A SFHSLHLLFEEYTNIPISLLFNEKEADDNDHEPQISPGGLEPPSEKHFRETEV 353
pHTLV-1B SFHNLHLLFEEYTNIPISLLFNEKEADDNDHEPQISPGGLEPPSEKHFRETEV 353

Rex (0%)

ATK HTLV-1A MPKTRRRPRRSQRKRPPTPWPTSQGLDRVFFSDTQSTCLETVYKATGAPSLGDYVRPAYI 60
pHTLV-1B MPKTRRRPRRSQRKRPPTPWPTSQGLDRVFFSDTQSTCLETVYKATGAPSLGDYVRPAYI 60

ATK HTLV-1A VTPYWPPVQSIRSPGTPSMDALSAQLYSSLSLDSPPSPPREPLRPSRSLPRQSLIQPPTF 120
pHTLV-1B VTPYWPPVQSIRSPGTPSMDALSAQLYSSLSLDSPPSPPREPLRPSRSLPRQSLIQPPTF 120

ATK HTLV-1A HPPSSRPCANTPPSEMDTWNPPLGSTSQPCLFQTPDSGPKTCTPSGEAPLSACTSTSFPP 180
pHTLV-1B HPPSSRPCANTPPSEMDTWNPPLGSTSQPCLFQTPDSGPKTCTPSGEAPLSACTSTSFPP 180

ATK HTLV-1A PSPGPSCPT 189
pHTLV-1B PSPGPSCPT 189

HBZ (1.4%)

ATK HTLV-1A MAASGLFRCLPVSCPEDLLVEELVDGLLSLEEELKDKEEEEAVLDGLLSLEEESRGRLRR 60
pHTLV-1B MAASGLFRCLPVPCPEDLLVEELVDGLLSLEEELKDKEEEEAVLDGLLSLEEESRGRLRR 60

ATK HTLV-1A GPPGEKAPPRGETHRDRQRRAEEKRKRKKEREKEEEKQTAEYLKRKEEEKARRRRRAEKK 120
pHTLV-1B GPPGEKAPPRGETHRDRQRRAEEKRKRKKEREKEEEKQIAEYLKRKEEEKARRRRQAEKK 120

ATK HTLV-1A AADVARRKQEEQERRERKWRQGAEKAKQHSARKEKMQELGIDGYTRQLEGEVESLEAERR 180
pHTLV-1B AADVARRKQEEQERRERKWRQGAEKAKQHSARKEKMQELGIDGYTRQLEGEVESLEAERR 180

ATK HTLV-1A KLLQEKEDLMGEVNYWQGRLEAMWLQ 206
pHTLV-1B KLLQEKEDLMGEVNYWQGRLEAMWLQ 206

P12 (6%)

ATK HTLV-1A MLFRLLSPLSPLALTALLLFLLPPSDVSGLLLRPPPAPCLLLFLPFQILSGLLFLLFLPLF 60
pHTLV-1B MLFRLLSPLSPLALTALLLFLLSPGDVSSLLLRLPPAPCLLLFLPFQILSNLLFLLFLPLF 60

ATK HTLV-1A FSLPLLLSPSLPITMRFPARWRFLPWKAPSQPAAAFLF 99
pHTLV-1B FSLPLLLSPSLPITMRFPARWRFLPWRAPSQPAAAFLF 99

P30 (2.5%)

ATK HTLV-1A	MALCCFAFSAPCLHLRSRRSCSSCFLLATSAAFFSARLLRRAFSSSFLFKYSAVCFSSSF	60
pHTLV-1B	MALCCFAFSAPCLHLRSRRSCSSCFLRATSAAFFSACLLRRAFSSSFLFKYSAICFSSSF	60
ATK HTLV-1A	SRSFFRFLFSSARRCRSRCVSPRGGAFSPGGPRRSRPRLSSSKDSKPSSTASSSSLSFNS	120
pHTLV-1B	SRSFFRFLFSSARRCRSRCVSPRGGAFSPGGPRRSRPRLSSSKDSKPSSTASSSSLSFNS	120
ATK HTLV-1A	SSKDNSPSTNSSTSRSSGHDTGKHRNSPADTKLTMLIISPLPRVWTESSFRIPSLRVWRL	180
pHTLV-1B	SSKDNSPSTNSSTSRSSGHGTGKHRNSPTDTKLTMLIISPLPRVWTESSFRIPSLRV * RL	180
ATK HTLV-1A	CTRRLVPHLWGTMFGPPTSSRPTGHLSRASDHLGPHRWTRYRLSSTVPYPSTPLLPHPENL	241
pHTLV-1B	CTRRLVPHLWGTMFGPPTSSRPTGHLSRASDHLGPHRWTRYRLSSTVPYPSTPLLPHPENL	241

GAG (0.95%)

ATK HTLV-1A	MGQIFSRSASPIPRPPRGLAAHHWLNFLQAAYRLEPGPSSYDFHQLKKFLKIALETP AR I	60
pHTLV-1B	MGQIFSRNASPIPRPPRGLAAHHWLNFLQAAYRLEPGPSSYDFHQLKKFLKIALETPVWI	60
ATK HTLV-1A	CPINYSLLASLLPKGYPGRVNEILHILIQTQAQIPSRPAPPPPSSPTHDPPDSDPQIPPP	120
pHTLV-1B	CPINYSLLASLLPKGYPGRVNEILHILIQTQAQIPSRPAPPPPSSPTHDPPDSDPQIPPP	120
ATK HTLV-1A	YVEPTAPQVLPVMHPHGAPPNHRPWQMKDLQAIKQEVSQAAPGSPQFMQTIRLAVQQFDP	180
pHTLV-1B	YVEPTAPQVLPVMHPHGAPPNHRPWQMKDLQAIKQEVSQAAPGSPQFMQTIRLAVQQFDP	180
ATK HTLV-1A	TAKDLQDLLQYLCSSLVASLHHQQLDSLISEAETRGITGYNPLAGPLRVQANNPQQQGLR	240
pHTLV-1B	TAKDLQDLLQYLCSSLVASLHHQQLDSLISEAETRGITGYNPLAGPLRVQANNPQQQGLR	240
ATK HTLV-1A	REYQQLWLAAFAALPGSAKDPSWASILQGLEEPYHAFVERLNIALDNGLPEGTPKDPILR	300
pHTLV-1B	REYQQLWLAAFAALPGSAKDPSWASILQGLEEPYHAFVERLNIALDNGLPEGTPKDPILR	300
ATK HTLV-1A	SLAYSNANKECQKLLQARGHTNSPLGDMLRACQTWTPKDKTKVLVVQPKKPPPNQPCFRC	360
pHTLV-1B	SLAYSNANKECQKLLQARGHTNSPLGDMLRACQTWTPKDKTKVLVVQPKKPPPNQPCFRC	360
ATK HTLV-1A	GKAGHWSRDCTQPRPPPGPCPLCQDPTHWKRDCPRLKPTIPEPEPEEDALLLDLPADIPH	420
pHTLV-1B	GKAGHWSQDCTQPRPPPGPCPLCQDPTHWKRDCPRLKPTIPEPEPEEDALLLDLPADIPH	420
ATK HTLV-1A	PKNSIGGEV	423
pHTLV-1B	PKNSIGGEV	423

PRO (0.60%)

ATK HTLV-1A	MTVLPIALFSSNTPLKNTSVLGAGGQTQDHFKLTSLPVLIRLPFRTTPIVLTSCLVDTKN	60
pHTLV-1B	MTVLPIALFSSNTPLKNTSVLGAGGQTQDHFKLTSLPVLIRLPFRTTPIVLTSCLVDTKN	60
ATK HTLV-1A	NWAIIGRDALQQCQGVLYLPEAKRPPVILPIQAPAVLGLEHLPRPPEISQFPLNQNASRP	120
pHTLV-1B	NWAIIGRDALQQCQGVLYLPEAKRPPVILPIQAPAVLGLEHLPRPPEISQFPLNQNASRP	120
ATK HTLV-1A	CNTWSGRPWRQAISNPTPGQGITQYSQLKRPMEPGDSSTTCGPLTL	166
pHTLV-1B	CNTWSGRPWRQAISNPTPGQEITQYSQLKRPMEPGDSSTTCGPLTL	166

POL/INT (1.4%)

ATK HTLV-1A	MQLAHILQPIRQAFPQCTILQYMDDILLASPSHEDLLLLSEATMASLISHGLPVSENKTQ	60
pHTLV-1B	MQLAHILQPIRQAFPQCTILQYMDDILLASPSHEDLLLLSEATMASLISHGLPVSENKTQ	60
ATK HTLV-1A	QTPGTIKFLGQIISPNHLTYDAVPTVPIRSRWALPELQALLGEIQWVSKGTPTLRQPLHS	120
pHTLV-1B	QTPGTIKFLGQIISPNHLTYDAVPTVPIRSRWALPELQALLGEIQWVSKGTPTLRQPLHS	120

```
ATK HTLV-1A   LYCALQRHTDPRDQIYLNPSQVQSLVQLRQALSQNCRSRLVQTLPLLGAIMLTLTGTTTV   180
pHTLV-1B      LYCALQRHTDPRDQIYLNPSQVQSLVQLRQALSQNCRSRLVQTLPLLGAIMLTLTGTTTV   180

ATK HTLV-1A   VFQSK EQWPLVWLHAPLPHTSQCPWGQLLASAVLLLDKYTLQSYGLLCQTIHHNISTQTF  240
pHTLV-1B      VFQSKQQWPLVWLHAPLPHTSQCPWGQLLASAVLLLDKYTLQSYGLLCQTIHHNISTQTF   240

ATK HTLV-1A   NQFIQTSDHPSVPILLHHSHRFKNLGAQTGELWNTFLKTAAPLAPVKALMPVFTLSPVII   300
pHTLV-1B      NQFIQTSDHPSVPILLHHSHRFKNLCAQTGELWNTFLKTAAPLAPVKALMPVFTLSPVIL   300


ATK HTLV-1A   NTAPCLFSDGSTSRAAYILWDKQILSQRSFPLPPPHKSAQRAELLGLLHGLSSARSWRCL   360
pHTLV-1B      NTAPCLFSDGSTSRAAYILWDKHILSQRSFPLPPPHKSAQRAELLGLLHGLSSARSWRCL   360

ATK HTLV-1A   NIFLDSKYLYHYLRTLALGTFQGRSSQAPFQALLPRLLSRKVVYLHHVRSHTNLPDPISR   420
pHTLV-1B      NIFLDSKYLYHYLRTLALGTFQGKSSQAPFQALLPRLLSRKVVYLHHVRSHTNLPDPISR   420

ATK HTLV-1A   LNALTDALLITPVLQLSPAELHSFTHCGQTALTLQGATTTEASNILRSCHACRGGNPQHQ   480
pHTLV-1B      LNALTDALLITPVLQLSPAELHSFTHCGQTALTLQGATTTEASNILRSCHACRKNNPQHQ   480

ATK HTLV-1A   MPRGHIRRGLLPNHIWQGDITHFKYKNTLYRLHVWVDTFSGAISATQKRKETSSEAISSL   540
pHTLV-1B      MPRGHIRRGLLPNHIWQGDITHFKYKNTLYRLHVWVDTFSGAISATQKRKETSSEAISSL   540

ATK HTLV-1A   LQAIAHLGKPSYINTDNGPAYISQDFLNMCTSLAIRHTTHVPYNPTSSGLVERSNGILKT   600
pHTLV-1B      LQAIAYLGKPSYINTDNGPAYISQDFLNMCTSLAIRHTTHVPYNPTSSGLVERSNGILKT   600

ATK HTLV-1A   LLYKYFTDKPDLPMDNALSIALWTINHLNVLTNCHKTRWQLHHSPRLQPIPETRSLSNKQ   660
pHTLV-1B      LLYKYFTDKPDLPMDNALSIALWTINHLNVLTNCHKTRWQLHHSPRLQPIPETHSLSNKQ   660

ATK HTLV-1A   THWYYFKLPGLNSRQWKGPQEALQEAAGAALIPVSASSAQWIPWRLLKRAACPRPVGGPA 720
pHTLV-1B      THWYYFKLPGLNSRQWKGPQEALQEAAGAALIPVSASSAQWIPWRLLKRAACPRPVGGPA  720

ATK HTLV-1A   DPKEKDLQHHG     731
pHTLV-1B      DPKEKDHQHHG     731
```

<u>GP62 ENV (2.05%)</u>

```
ATK HTLV-1A   MGKFLATLILFFQFCPLIFGDYSPSCCTLTIGVSSYHSKPCNPAQPVCSWTLDLLALSAD   60
pHTLV-1B      MGKFLATLILFFQFCPLILGDYSPSCCTLTIGVSSYHSEPCNPAQPVCSWTLDLLALSAD   60

ATK HTLV-1A   QALQPPCPNLVSYSSYHATYSLYLFPHWTKKPNRNGGGYYSASYSDPCSLKCPYLGCQSW   120
pHTLV-1B      QALQPPCPNLVGYSSYHATYSLYLFPHWI KKPNRNGGGYYSASYSDPCSLKCPYLGCQSW  120

ATK HTLV-1A   TCPYTGAVSSPYWKFQHDVNFTQEVSRLNINLHFSKCGFPFSLLVDAPGYDPIWFLNTEP   180
pHTLV-1B      TCPYTGAVSSPYWKFQQDVNFTQEVSRLNINLHFSKCGFPFSLLVDAPGYDPIWFLNTEP   180

ATK HTLV-1A   SQLPPTAPPLLPHSNLDHILEPSIPWKSKLLTLVQLTLQSTNYTCIVCIDRASLSTWHVL   240
pHTLV-1B      SQLPPTAPPLLPHSNLDHILEPSIPWKSKLLTLVQLTLQSTNYTCIVCIDRASLSTWHVL   240

ATK HTLV-1A   YSPNVSVPSSSSTPLLYPSLALPAPHLTLPFNWTHCFDPQIQAIVSSPCHNSLILPPFSL   300
pHTLV-1B      YSPNVSVPSSSSTPLLYPSLALPAPHLTLPFNWTHCFDPQIQAIVSSPCHNSLILPPFSL   300

ATK HTLV-1A   SPVPTLGSRSRRAVPVAVWLVSALAMGAGVAGGITGSMSLASGKSLLHEVDKDISQLTQA   360
pHTLV-1B      SPVPTLGSRSRRAVPVAVWLVSALAMGAGVAGGITGSMSLASGKSLLHEVDKDISQLTQA   360

ATK HTLV-1A   IVKNHKNLLKIAQYAAQNRRGLDLLFWEQGGLCKALQEQCRFPNITNSHVPILQERPPLE   420
pHTLV-1B      IVKNHKNLLKIAQYAAQNRRGLDLLFWEQGGLCKALQEQCCFLNITNSHVSILQERPPLE   420

ATK HTLV-1A   NRVLTGWGLNWDLGLSQWAREALQTGITLVALLLLVILAGPCILRQLRHLPSRVRYPHYS   480
pHTLV-1B      NRVLTG* GLNWDLGLSQWAREALQTGITLVALLLLVILAGPCILRQLRHLPSRVRYPHYS  480

ATK HTLV-1A   LIKPESSL        488
pHTLV-1B      LINPESSL        488
```
