# Supplementary material for: Characterization of HTLV-1 Infectious Molecular Clone Isolated from Patient with HAM/TSP and Immortalization of Human Primary T-Cell Lines
Source: Viruses. 2024 Nov 9;16(11):1755. doi: 10.3390/v16111755 (PMC11599126; doi:10.3390/v16111755)
Supplement: Supplementary file 1 [file viruses-16-01755-s001.zip › Supplemental S4 GAG align.pdf]

HTLV-1 GAG ALIGNMENTS BETWEEN JAPANESE AND BRAZILIAN HAM/TSP ISOLATES (FULL GENOME SEQUENCE) AND OTHER HTLV-1 MOLECULAR CLONES

**PBST**	MGQIFSRNASPIPRPPRGLAAHHWLNFLQAAYRLEPGPSSYDFHQLKKFLKIALETPVWICPINYSLLASLLPKGYPGRVNEILHILIQTQAQ

**HAM1**	MGQIFSRSASPIPRPPRGLAAHHWLNFLQAAYRLEPGPSSYDFHQLKKFLKIALETPVWICPINYSLLASLLPKGYPGRVNEILHILIQTQAQ
**HAM2**	MGQIFSRSASPIPRPPRGLAAHHWLNFLQAAYRLEPGPSSYDFHQLKKFLKIALETPVWICPINYSLLASLLPKGYPGRVNEILHILIQTQAQ
**HAM3**	MGQIFSRSASPIPRPPRGLAAHHWLNFLQAAYRLEPGPSSYDFHQLKKFLKIALETPVWICPINYSLLASLLPKGYPGRVNEILHILIQTQAQ
**HAM4**	MGQIFSRSASPIPRPPRGLAAHHWLNFLQAAYRLEPGPSSYDFHQLKKFLKIALETPVWICPINYSLLASLLPKGYPGRVNEILHILIQTQAQ
**HAM5**	MGQIFSRSASPIPRPPRGLAAHHWLNFLQAAYRLEPGPSSYDFHQLKKFLKIALETPVWICPINYSLLASLLPKGYPGRVNEILHILIQTQAQ
**HAM6**	MGQIFSRSASPIPRPPRGLAAHHWLNFLQAAYRLEPGPSSYDFHQLKKFLKIALETPVWICPINYSLLASLLPKGYPGRVNEILHILIQTQAQ
**HAM7**	MGQIFSRSASPIPRPPRGLAAHHWLNFLQAAYRLEPGPSSYDFHQLKKFLKIALETPVWICPINYSLLASLLPKGYPGRVNEILHILIQTQAQ
**HAM8**	MGQIFSRSASPIPRPPRGLAAHHWLNFLQAAYRLEPGPSSYDFHQLKKFLKIALETPVWICPINYSLLASLLPKGYPGRVNEILHILIQTQAQ
**HAM9**	MGQIFSRSASPIPRPPRGLAAHHWLNFLQAAYRLEPGPSSYDFHQLKKFLKIALETPVWICPINYSLLASLLPKGYPGRVNEILHILIQTQAQ
**HAM10**	MGQIFSRSASPIPRPPRGLAAHHWLNFLQAAYRLEPGPSSYDFHQLKKFLKIALETPVWICPINYSLLASLLPKGYPGRVNEILHILIQTQAQ
**HAM11**	MGQIFSRSASPIPRPPRGLAAHHWLNFLQAAYRLEPGPSSYDFHQLKKFLKIALETPVWICPINYSLLASLLPKGYPGRVNEILHILIQTQAQ
**HAM12**	MGQIFSRSASPIPRPPRGLAAHHWLNFLQAAYRLEPGPSSYDFHQLKKFLKIALETPVWICPINYSLLASLLPKGYPGRVNEILHILIQTQAQ

**HAM1**	MGQIFSRSASPIPRPPRGLAAHHWLNFLQAAYRLEPGPSSYDFHQLKKFLKIALETPVWICPINYSLLASLLPKGYPGRVNEILHILIQTQAQ
**HAM2**	MGQIFSRSASPIPRPPRGLAAHHWLNFLQAAYRLEPGPSSYDFHQLKKFLKIALETPVWICPINYSLLASLLPKGYPGRVNEILHILIQTQAQ
**HAM3**	MGQIFSRSASPIPRPPRGLAAHHWLNFLQAAYRLEPGPSSYDFHQLKKFLKIALETPVWICPINYSLLASLLPKGYPGRVNEILHILIQTQAQ
**HAM4**	MGQIFSRSASPIPRPPRGLAAHHWLNFLQAAYRLEPGPSSYDFHQLKKFLKIALETPVWICPINYSLLASLLPKGYPGRVNEILHILIQTQAQ
**HAM5**	MGQIFSRSASPIPRPPRGLAAHHWLNFLQAAYRLEPGPSSYDFHQLKKFLKIALETPVWICPINYSLLASLLPKGYPGRVNEILHILIQTQAQ
**HAM6**	MGQIFSRSASPIPRPPRGLAAHHWLNFLQAAYRLEPGPSSYDFHQLKKFLKIALETPVWICPINYSLLASLLPKGYPGRVNEILHILIQTQAQ
**HAM7**	MGQIFSRSASPIPRPPRGLAAHHWLNFLQAAYRLEPGPSSYDFHQLKKFLKIALETPVWICPINYSLLASLLPKGYPGRVNEILHILIQTQAQ
**HAM8**	MGQIFSRSASPIPRPPRGLAAHHWLNFLQAAYRLEPGPSSYDFHQLKKFLKIALETPVWICPINYSLLASLLPKGYPGRVNEILHILIQTQAQ
**HAM9**	MGQIFSRSASPIPRPPRGLAAHHWLNFLQAAYRLEPGPSSYDFHQLKKFLKIALETPVWICPINYSLLASLLPKGYPGRVNEILHILIQTQAQ
**HAM10**	MGQIFSRSASPIPRPPRGLAAHHWLNFLQAAYRLEPGPSSYDFHQLKKFLKIALETPVWICPINYSLLASLLPKGYPGRVNEILHILIQTQAQ

**ACH**	MGQIFSRSASPIPRPPRGLAAHHWLNFLQAAYRLEPGPSSYDFHLLKKFLKIALETPVWICPINYSLLASLLPKGYPGRVNEILHILIQTQAQ
**K30p**	MGQIFSRSASPIPRPPRGLAAHHWLNFLQAAYRLEPGPSSYDFHQLKKFLKIALETPVWICPINYSLLASLLPKGYPGRVNEILHILIQTQAQ

**PBST**	IPSRPAPPPPSSPTHDPPDSDPQIPPPYVEPTAPQVLPVMHPHGAPPNHRPWQMKDLQAIKQEVSQAAPGSPQFMQTIRLAVQQFDPTAKDLQ

**HAM1**	IPSRPAPPPPSSPTHDPPDSDPQIPPPYVEPTAPQVLPVMHPHGAPPNHRPWQMKDLQAIKQEVSQAAPGSPQFMQTIRLAVQQFDPTAKDLQ
**HAM2**	IPSRPAPPPPSSPTHDPPDSDPQIPPPYVEPTAPQVLPVMHPHGAPPNHRPWQMKDLQAIKQEVSQAAPGSPQFMQTIRLAVQQFDPTAKDLQ
**HAM3**	IPSRPAPPPPSSPTHDPSDSDPQIPPPYVEPTAPQVLPVMHPHGAPPNHRPWQMKDLQAIKQEVSQAAPGSPQFMQTIRLAVQQFDPTAKDLQ
**HAM4**	IPSRPAPPPPSSPTHDPPDSDPQIPPPYVEPTAPQVLPVMHPHGAPPNHRPWQMKDLQAIKQEVSQAAPGSPQFMQTIRLAVQQFDPTAKDLQ
**HAM5**	IPSRPAPPPPSSPTHDPPDSDPQIPPPYVEPTAPQVLPVMHPHGAPPNHRPWQMKDLQAIKQEVSQAAPGSPQFMQTIRLAVQQFDPTAKDLQ
**HAM6**	IPSRPAPPPPSSPTHDPPDSDPQIPPPYVEPTAPQVLPVMHPHGAPPNHRPWQMKDLQAIKQEVSQAAPGSPQFMQTIRLAVQQFDPTAKDLQ
**HAM7**	IPSRPAPPPPSSPTHDPPDSDPQIPPPYVEPTAPQVLPVMHPHGAPPNHRPWQMKDLQAIKQEVSQAAPGSPQFMQTIRLAVQQFDPTAKDLQ
**HAM8**	IPSRPAPPPPSSPTHDPPDSDPQIPPPYVEPTAPQVLPVMHPHGAPPNHRPWQMKDLQAIKQEVSQAAPGSPQFMQTIRLAVQQFDPTAKDLQ
**HAM9**	IPSRPAPPPPSSPTHDPPDSDPQIPPPYVEPTAPQVLPVMHPHGAPPNHRPWQMKDLQAIKQEVSQAAPGSPQFMQTIRLAVQQFDPTAKDLQ
**HAM10**	IPSRPAPPPPSSPTHDPPDSDPQIPPPYVEPTAPQVLPVMHPHGAPPNHRPWQMKDLQAIKQEVSQAAPGSPQFMQTIRLAVQQFDPTAKDLQ
**HAM11**	IPSRPAPPPPSSPTHDPPDSDPQIPPPYVEPTAPQVLPVMHPHGAPPNHRPWQMKDLQAIKQEVSQAAPGSPQFMQTIRLAVQQFDPTAKDLQ
**HAM12**	IPSRPAPPPPSSPTHDPSDSDPQIPPPYVEPTAPQVLPVMHPHGAPPNHRPWQMKDLQAIKQEVSQAAPGSPQFMQTIRLAVQQFDPTAKDLQ

**HAM1**	IPSRPAPPPPSSPTHDPPDSDPQIPPPYVEPTAPQVLPVMHPHGAPPNHRPWQMKDLQAIKQEVSQAAPGSPQFMQTIRLAVQQFDPTAKDLQ
**HAM2**	IPSRPAPPPPSSPTHDPPDSDPQIPPPYVEPTAPQVLPVMHPHGAPPNHRPWQMKDLQAIKQEVSQAAPGSPQFMQTIRLAVQQFDPTAKDLQ
**HAM3**	IPSRPAPPPPSSPTHDPPDSDPQIPPPYVEPTAPQVLPVMHPHGAPPNHRPWQMKDLQAIKQEVSQAAPGSPQFMQTIRLAVQQFDPTAKDLQ
**HAM4**	IPSRPAPPPPSSPTHDPPDSDPQIPPPYVEPTAPQVLPVMHPHGAPPNHRPWQMKDLQAIKQEVSQAAPGSPQFMQTIRLAVQQFDPTAKDLQ
**HAM5**	IPSRPAPPPPSSPTHDPPDSDPQIPPPYVEPTAPQVLPVMHPHGAPPNHRPWQMKDLQAIKQEVSQAAPGSPQFMQTIRLAVQQFDPTAKDLQ
**HAM6**	IPSRPAPPPPSSPTHDPPDSDPQIPPPYVEPTAPQVLPVMHPHGAPPNHRPWQMKDLQAIKQEVSQAAPGSPQFMQTIRLAVQQFDPTAKDLQ
**HAM7**	IPSRPAPPPPSSPTHDPPDSDPQIPPPYVEPTAPQVLPVMHPHGAPPNHRPWQMKDLQAIKQEVSQAAPGSPQFMQTIRLAVQQFDPTAKDLQ
**HAM8**	IPSRPAPPPPSSPTHDPPDSDPQIPPPYVEPTAPQVLPVMHPHGAPPNHRPWQMKDLQAIKQEVSQAAPGSPQFMQTIRLAVQQFDPTAKDLQ
**HAM9**	IPSRPAPPPPSSPTHDPPDSDPQIPPPYVEPTAPQVLPVMHPHGAPPNHRPWQMKDLQAIKQEVSQAAPGSPQFMQTIRLAVQQFDPTAKDLQ
**HAM10**	IPSRPAPPPPSSPTHDPPDSDPQIPPPYVEPTAPQVLPVMHPHGAPPNHRPWQMKDLQAIKQEVSQAAPGSPQFMQTIRLAVQQFDPTAKDLQ

**ACH**	IPSRPAPPPPSSPTHDPPDSDPQIPPPYVEPTAPQVLPVMHPHGAPPNHRPWQMKDLQAIKQEVSQAAPGSPQFMQTIRLAVQQFDPTAKDLQ
**K30p**	IPSRPAPPPPSSPTHDPPDSDPQIPPPYVEPTAPQVLPVMHPHGAPPNHRPWQMKDLQAIKQEVSQAARGSPQFMQTIRLAVQQFDPTAKDLQ

PL

**PBST**	DLLQYLCSSLVASLHHQQLDSLISEAETRGITGYNPLAGPLRVQANNPQQQGLRREYQQLWLAAFAALPGSAKDPSWASILQGLEEPYHAFVE

**HAM1**	DLLQYLCSSLVASLHHQQLDSLISEAETRGITGYNPLAGPLRVQANNPQQQGLRREYQQLWLAAFAALPGSAKDPSWASILQGLEEPYHAFVE
**HAM2**	DLLQYLCSSLVASLHHQQLDSLISEAETRGITGYNPLAGPLRVQANNPQQQGLRREYQQLWLAAFAALPGSAKDPSWASILQGLEEPYHAFVE
**HAM3**	DLLQYLCSSLVASLHHQQLDSLISEAETRGITGYNPLAGPLRVQANNPQQQGLRREYQQLWLAAFAALPGSAKDPSWASILQGLEEPYHAFVE
**HAM4**	DLLQYLCSSLVASLHHQQLDSLISEAETRGITGYNPLAGPLRVQANNPQQQGLRREYQQLWLAAFAALPGSAKDPSWASILQGLEEPYHAFVE
**HAM5**	DLLQYLCSSLVASLHHQQLDSLISEAETRGITGYNPLAGPLRVQANNPQQQGLRREYQQLWLAAFAALPGSAKDPSWASILQGLEEPYHAFVE
**HAM6**	DLLQYLCSSLVASLHHQQLDSLISEAETRGITGYNPLAGPLRVQANNPQQQGLRREYQQLWLAAFAALPGSAKDPSWASILQGLEEPYHAFVE
**HAM7**	DLLQYLCSSLVASLHHQQLDSLISEAETRGITGYNPLAGPLRVQANNPQQQGLRREYQQLWLAAFAALPGSAKDPSWASILQGLEEPYHAFVE
**HAM8**	DLLQYLCSSLVASLHHQQLDSLISEAETRGITGYNPLAGPLRVQANNPQQQGLRREYQQLWLAAFAALPGSAKDPSWASILQGLEEPYHAFVE
**HAM9**	DLLQYLCSSLVASLHHQQLDSLISEAETRGITGYNPLAGPLRVQANNPQQQGLRREYQQLWLAAFAALPGSAKDPSWASILQGLEEPYHAFVE
**HAM10**	DLLQYLCSSLVASLHHQQLDSLISEAETRGITGYNPLAGPLRVQANNPQQQGLRREYQQLWLAAFAALPGSAKDPSWASILQGLEEPYHAFVE
**HAM11**	DLLQYLCSSLVASLHHQQLDSLISEAETRGITGYNPLAGPLRVQANNPQQQGLRREYQQLWLAAFAALPGSAKDPSWASILQGLEEPYHAFVE
**HAM12**	DLLQYLCSSLVASLHHQQLDSLISEAETRGITGYNPLAGPLRVQANNPQQQGLRREYQQLWLAAFAALPGSAKDPSWASILQGLEEPYHAFVE

**HAM1**	DLLQYLCSSLVASLHHQQLDSLISEAETRGITGYNPLAGPLRVQANNPQQQGLRREYQQLWLAAFAALPGSAKDPSWASILQGLEEPYHAFVE
**HAM2**	DLLQYLCSSLVASLHHQQLDSLISEAETRGITGYNPLAGPLRVQANNPQQQGLRREYQQLWLAAFAALPGSAKDPSWASILQGLEEPYHAFVE
**HAM3**	DLLQYLCSSLVASLHHQQLDSLISEAETRGITGYNPLAGPLRVQANNPQQQGLRREYQQLWLAAFAALPGSAKDPSWASILQGLEEPYHAFVE
**HAM4**	DLLQYLCSSLVASLHHQQLDSLISEAETRGITGYNPLAGPLRVQANNPQQQGLRREYQQLWLAAFAALPGSAKDPSWASILQGLEEPYHAFVE
**HAM5**	DLLQYLCSSLVASLHHQQLDSLISEAETRGITGYNPLAGPLRVQANNPQQQGLRREYQQLWLAAFAALPGSAKDPSWASILQGLEEPYHAFVE
**HAM6**	DLLQYLCSSLVASLHHQQLDSLISEAETRGITGYNPLAGPLRVQANNPQQQGLRREYQQLWLAAFAALPGSAKDPSWASILQGLEEPYHAFVE
**HAM7**	DLLQYLCSSLVASLHHQQLDSLISEAETRGITGYNPLAGPLRVQANNPQQQGLRREYQQLWLAAFAALPGSAKDPSWASILQGLEEPYHAFVE
**HAM8**	DLLQYLCSSLVASLHHQQLDSLISEAETRGITGYNPLAGPLRVQANNPQQQGLRREYQQLWLAAFAALPGSAKDPSWASILQGLEEPYHAFVE
**HAM9**	DLLQYLCSSLVASLHHQQLDSLISEAETRGITGYNPLAGPLRVQANNPQQQGLRREYQQLWLAAFAALPGSAKDPSWASILQGLEEPYHAFVE
**HAM10**	DLLQYLCSSLVASLHHQQLDSLISEAETRGITGYNPLAGPLRVQANNPQQQGLRREYQQLWLAAFAALPGSAKDPSWASILQGLEEPYHAFVE

**ACH**	DLLQYLCSSLVASLHHQQLDSLISEAETRGITGYNPLAGPLRVQANNPQQQGLRREYQQLWLAAFAALPGSAKDPSWASILQGLEEPYHAFVE
**K30p**	DLLQYLCSSLVASLHHQQLDSLISEAETRGITGYNPLAGPLRVQANNPQQQGLRREYQQLWLAAFAALPGSAKDPSWASILQGLEEPYHAFVE

**PBST**	RLNIALDNGLPEGTPKDPILRSLAYSNANKECQKLLQARGHTNSPLGDMLRACQTWTPKDKTKVLVVQPKKPPPNQPCFRCGKAGHWSQDCTQ

**HAM1**	RLNIALDNGLPEGTPKDPILRSLAYSNANKECQKLLQARGHTNSPLGDMLRACQTWTPKDKTKVLVVQPKKPPPNQPCFRCGKAGHWSRDCTQ
**HAM2**	RLNIALDNGLPEGTPKDPILRSLAYSNANKECQKLLQARGHTNSPLGDMLRACQTWTPKDKTKVLVVQPKKPPPNQPCFRCGKAGHWSRDCTQ
**HAM3**	RLNIALDNGLPEGTPKDPILRSLAYSNANKECQKLLQARGHTNSPLGDMLRACQTWTPKDKTKVLVVQPKKPPPNQPCFRCGKAGHWSRDCTQ
**HAM4**	RLNIALDNGLPEGTPKDPILRSLAYSNANKECQKLLQARGHTNSPLGDMLRACQTWTPKDKTKVLVVQPKKPPPNQPCFRCGKAGHWSRDCTQ
**HAM5**	RLNIALDNGLPEGTPKDPILRSLAYSNANKECQKLLQARGHTNSPLGDMLRACQTWTPKDKTKVLVVQPKKPPPNQPCFRCGKAGHWSRDCTQ
**HAM6**	RLNIALDNGLPEGTPKDPILRSLAYSNANKECQKLLQARGHTNSPLGDMLRACQTWTPKDKTKVLVVQPKKPPPNQPCFRCGKAGHWSRDCTQ
**HAM7**	RLNVALDNGLPEGTPKDPILRSLAYSNANKECQKLLQARGHTNSPLGDMLRACQTWTPKDKTKVLVVQPKKPPPNQPCFRCGKAGHWSRDCTQ
**HAM8**	RLNIALDNGLPEGTPKDPILRSLAYSNANKECQKLLQARGHTNSPLGDMLRACQTWTPKDKTKVLVVQPKKPPPNQPCFRCGKAGHWSRDCTQ
**HAM9**	RLNIALDNGLPEGTPKDPILRSLAYSNANKECQKLLQARGHTNSPLGDMLRACQTWTPKDKTKVLVVQPKKPPPNQPCFRCGKAGHWSRDCTQ
**HAM10**	RLNIALDNGLPEGTPKDPILRSLAYSNANKECQKLLQARGHTNSPLGDMLRACQTWTPKDKTKVLVVQPKKPPPNQPCFRCGKAGHWSRDCTQ
**HAM11**	RLNIALDNGLPEGTPKDPILRSLAYSNANKECQRLLQARGHTNSPLGDMLRACQTWTPKDKTKVLVVQPKKPPPNQPCFRCGKAGHWSRDCTQ
**HAM12**	RLNIALDNGLPEGTPKDPILRSLAYSNANKECQKLLQARGHTNSPLGDMLRACQTWTPKDKTKVLVVQPKKPPPNQPCFRCGKAGHWSRDCTQ

**HAM1**	RLNIALDNGLPEGTPKDPILRSLAYSNANKECQKLLQARGHTNSPLGDMLRACQTWTPKDKTKVLVVQPKKPPPNQPCFRCGKAGYRSRDCTQ
**HAM2**	RLNIALDNGLPEGTPKDPILRSLAYSNANKECQKLLQARGHTNSPLGDMLRACQTWTPKDKTKVLVVPPKKPPPNQPCFRCGKAGHWSRDCTQ
**HAM3**	RLNIALDNGLPEGTPKDPILRSLAYSNANKECQKLLQARGHTNSPLGDMLRACQTWTPKDKTKVLVVPPKKPPPNQPCFRCGKAGHWSRDCTQ
**HAM4**	RLNIALDNGLPEGTPKDPILRSLAYSNANKECQKLLQARGHTNSPLGDMLRACQTWTPKDKTKVLVVQPKKPPPNQPCFRCGKAGHWSRDCTQ
**HAM5**	RLNIALDNGLPEGTPKDPILRSLAYSNANKECQKLLQARGHTNSPLGDMLRACQTWTPKDKTKVLVVPPKKPPPNQPCFRCGKAGHWSRDCTQ
**HAM6**	RLNIALDNGLPEGTPKDPILRSLAYSNANKECQKLLQARGHTNSPLGDMLRACQTWTPKDKTKVLVVQPKKPPPNGQCFRCGKAGHWSRDCTQ
**HAM7**	RLNIALDNGLPEGTPKDPILRSLAYSNANKECQKLLQARGHTNSPLGDMLRACQTWTPKDKTKVLVVQPKKPPPNQPCFRCGKAGHWSRDCTQ
**HAM8**	RLNIALDNGLPEGTPKDPILRSLAYSNANKECQKLLQARGHTNSPLGDMLRACQTWTPKDKTKVLVVQPKKPPPNQPCFRCGKAGHWSRDCTQ
**HAM9**	RLNIALDNGLPEGTPKDPILRSLAYSNANKECQKLLQARGHTNSPLGDMLRACQTWTPKDKTKVLVVQPKKPPPNQPCFRCGKAGHWSRDCTQ
**HAM10**	RLNIALDNGLPEGTPKDPILRSLAYSNANKECQKLLQARGHTNSPLGDMLRACQTWTPKDKTKVLVVQPKKPPPNQPCFRCGKAGHWSRDCTQ

**ACH**	RLNVALDNGLPEGTPKDPILRSLAYSNANKECQKLLQARGHTNSPLGDMLRACQTWTPKDKTKVLVVQPKKPPPNQPCFRCGKAGHWSRDCTQ
**K30p**	RLNIALDNGLPEGTPKDPILRSLAYSNANKECQKLLQARGHTNSPLGDMLRACQTWTPKDKTKVLVVQPKKPPPNQPCFRCGKAGHWSRDCTQ

**PBST**	PRPPPGPCPLCQDPTHWKRDCPRLKPTIPEPEPEEDALLLDLPADIPHPKNSIGGEV

**HAM1**	PRPPPGPCPLCQDPTHWKRDCPRLKPTIPEPEPEEDALLLDLPADIPHPKNSIGGEV
**HAM2**	PRPPPGPCPLCQDPTHWKRDCPRLKPTIPEPEPEEDALLLDLPADIPHPKNSIGGEV
**HAM3**	PRPPPGPCPLCQDPTHWKRDCPRLKPTIPEPEPEEDALLLDLPADIPHPKNSIGGEV
**HAM4**	PRPPPGPCPLCQDPTHWKRDCPRLKPTIPEPEPEEDALLLDLPADIPHPKNSIGGEV
**HAM5**	PRPPPGPCPLCQDPTHWKRDCPRLKPTIPEPEPEEDALLLDLPADIPHPKNSIGGEV
**HAM6**	PRPPPGPCPLCQDPTHWKRDCPRLKPTIPEPEPEEDALLLDLPADIPHPKNSIGGEV
**HAM7**	PRPPPGPCPLCQDPTHWKRDCPRLKPTIPEPEPEEDALLLDLPADIPHPKNSIGGEV
**HAM8**	PRPPPGPCPLCQDPTHWKRDCPRLKPTIPEPEPEEDAVLLDLPADIPHPKNSIGGEV
**HAM9**	PRPPPGPCPLCQDPTHWKRDCPRLKPTIPEPEPEEDALLLDLPADIPHPKNSIGGEV
**HAM10**	PRPPPGPCPLCQDPTHWKRDCPRLKPTIPEPEPEEDALLLDLPADIPHPKNSIGGEV
**HAM11**	PRPPPGPCPLCQDPTHWKRDCPRLKPTIPEPEPEEDALLLDLPADIPHPKNSIGGEV
**HAM12**	PRPPPGPCPLCQDPTHWKRDCPRLKPTIPEPEPEEDALLLDLPADIPHPKNSIGGEV

**HAM1**	PSPPPGPCPLCQDPTHWKRDCPRLKATIPEPEPEEDALLLDLPADIPHPKNSIGGEV
**HAM2**	PRPPPGPCPLCQDPTHWKRDCPRLKPTIPEPEPEEDALLLDLPADIPHPKNSIGGEV
**HAM3**	PRPPPGPCPLCQDPTHWKRDCPRLKPTIPEPEPEEDALLLDLPADIPHPKNSIGGEV
**HAM4**	PRPPPGPCPLCQDPTHWKRDCPRLKPTIPEPEPEEDALLLDLPADIPHPKNSIGGEV
**HAM5**	PRPPPGPCPLCQDPTHWKRDCPRLKPTIPEPEPEEDALLLDLPADIPHPKNSIGGEV
**HAM6**	PRPPPGPCPLCQDPTHWKRDCPRLKPTIPEPEPEEDALLLDLPADIPHPKNSIGGEV
**HAM7**	PRPPPGPCPLCQDPTHWKRDCPRLKPTIPEPEPEEDALLLDLPADIPHPKNSIGGEV
**HAM8**	PRPPPGPCPLCQDPTHWKRDCPRLKPTIPEPEPEEDALLLDLPADIPHPKNSIGGEV
**HAM9**	PRPPPGPCPLCQDPTHWKRDCPRLKPTIPEPEPEEDALLLDLPADIPHPKNSIGGEV
**HAM10**	PRPPPGPCPLCQDPTHWKRDCPRLKPTIPEPEPEEDALLLDLPADIPHPKNSIEGEV

**ACH**	PRPPPGPCPLCQDPTHWKRDCPRLKPTIPEPEPEEDALLLDLPADIPHPKNSIGGEV
**K30p**	PRPPPGPCPLCQDPTHWKRDCPRLKPTIPEPEPEEDALLLDLPADIPHPKNSIGGEV
