# Supplementary material for: Characterization of HTLV-1 Infectious Molecular Clone Isolated from Patient with HAM/TSP and Immortalization of Human Primary T-Cell Lines
Source: Viruses. 2024 Nov 9;16(11):1755. doi: 10.3390/v16111755 (PMC11599126; doi:10.3390/v16111755)
Supplement: Supplementary file 1 [file viruses-16-01755-s001.zip › Supplemental S5 Pro align.pdf]

HTLV-1 PRO ALIGNMENTS BETWEEN JAPANESE AND BRAZILIAN HAM/TSP ISOLATES (FULL GENOME SEQUENCE) AND OTHER HTLV-1 MOLECULAR CLONES

```
PBST    MTVLPIALFSSNTPLKNTSVLGAGGQTQDHFKLTSLPVLIRLPFRTTPIVLTSCLVDTKNNWAIIGRDALQQCQGVLYLPEAKRPPVIL

HAM1    MTVLPIALFSSNTPLKNTSVLGAGGQTQDHFKLTSLPVLIRLPFRTTPIVLTSCLVDTKNNWAIIGRDALQQCQGVLYLPEAKRPPVIL
HAM2    MTVLPIALFSSNTPLKNTSVLGAGGQTQDHFKLTSLPVLIRLPFRTTPIVLTSCLVDTKNNWAIIGRDALQQCQGVLYLPEAKGPPVIL
HAM3    MTVLPIALFSSNTPLKNTSVLGAGGQTQDHFKLTSLPVLIRLPFRTTPIVLTSCLVDTKNNWAIIGRDALQQCQGVLYLPEAKRPPVIL
HAM4    MTVLPIALFSSNTPLKNTSVLGAGGQTQDHFKLTSLPVLIRLPFRTTPIVLTSCLVDTKNNWAIIGRDALQQCQGVLYLPEAKRPPVIL
HAM5    MTVLPIALFSSNTPLKNTSVLGAGGQTQDHFKLTSLPVLIRLPFRTTPIVLTSCLVDTKNNWAIIGRDALQQCQGVLYLPEAKGPPVIL
HAM6    MTVLPIALFSSNTPLKNTSVLGAGGQTQDHFKLTSLPVLIRLPFRTTPIVLTSCLVDTKNNWAIIGRDALQQCQGVLYLPEAKGPPVIL
HAM7    MTVLPIALFSSNTPLKNTSVLGAGGQTQDHFKLTSLPVLIRLPFRTTPIVLTSCLVDTKNNWAIIGRDALQQCQGVLYLPEAKGPPVIL
HAM8    MTVLPIALFSSNTPLKNTSVLGAGGQTQDHFKLTSLPVLIRLPFRTTPIVLTSCLVDTKNNWAIIGRDALQQCQGVLYLPEAKGPPVIL
HAM9    MTVLPIALFSSNTPLKNTSVLGAGGQTQDHFKLTSLPVLIRLPFRTTPIVLTSCLVDTKNNWAIIGRDALQQCQGVLYLPEAKGPPVIL
HAM10   MTVLPIALFSSNTPLKNTSVLGAGGQTQDHFKLTSLPVLIRLPFRTTPIVLTSCLVDTKNNWAIIGRDALQQCQGVLYLPEAKGPPVIL
HAM11   MTVLPIALFSSNTPLKNTSVLGAGGQTQDHFKLTSLPVLIRLPFRTTPIVLTSCLVDTKNNWAIIGRDALQQCQGVLYLPEAKGPPVIL
HAM12   MTVLPIALFSSNTPLKNTSVLGAGGQTQDHFKLTSLPVLIRLPFRTTPIVLTSCLVDTKNNWAIIGRDALQQCQGVLYLPEAKGPPVIL

HAM1    MTVLPIALFSSNTPLKNTSVLGAGGQTQDHFKLTSLPVLIRLPFRTTPIVLTSCLVDTKNNWAIIGRDALQQCQGVLYLPEAKGPPVIL
HAM2    MTVLPIALFSSNTPLKNTSVLGAGGQTQDHFKLTSLPVLIRLPFRTTPIVLTSCLVDTKNNWAIIGRDALQQCQGVLYLPEAKGPPVIL
HAM3    MTVLPIALFSSNTPLKNTSVLGAGGQTQDHFKLTSLPVLIRLPFRTTPIVLTSCLVDTKNNWAIIGRDALQQCQGVLYLPEAKGPPVIL
HAM4    MTVLPIALFSSNTPLKNTSVLGAGGQTQDHFKLTSLPVLIRLPFRTTPIVLTSCLVDTKNNWAIIGRDALQQCQGVLYLPEAKRPPVIL
HAM5    MTVLPIALFSSNTPLKNTSVLGAGGQTQDHFKLTSLPVLIRLPFRTTPIVLTSCLVDTKNNWAIIGRDALQQCQGVLYLPEAKGPPVIL
HAM6    MTVLPIALFSSNTPLKNTSVLGAGGQTQDHFKLTSLPVLIRLPFRTTPIVLTSCLVDTKNNWAIIGRDALQQCQGVLYLPEAKRPPVIL
HAM7    MTVLPIALFSSNTPLKNTSVLGAGGQTQDHFKLTSLPVLIRLPFRTTPIVLTSCLVDTKNNWAIIGRDALQQCQGVLYLPEAKGPPVIL
HAM8    MTVLPIALFSSNTPLKNTSVLGAGGQTQDHFKLTSLPVLIRLPFRTTPIVLTSCLVDTKNNWAIIGRDALQQCQGVLYLPEAKGPPVIL
HAM9    MTVLPIALFSSNTPLKNTSVLGAGGQTQDHFKLTSLPVLIRLPFRTTPIVLTSCLVDTKNNWAIIGRDALQQCQGVLYLPEAKGPPVIL
HAM10   MTVLPIALFSSNTPLKNTSVLGAGGQTQDHFKLTSLPVLIRLPFRTTPIVLTSCLVDTKNNWAIIGRDALQQCQGVLYLPEAKGPPVIL

ACH     MTVLPIALFSSNTPLKNTSVLGAGGQTEDHFKLTSLPVLIRLPFRTTPIVLTSCLVDTKNNWAIIGRDALQQCQGVLYLPEAKRPPVIL
K30p    MTVLPIALFSSNTPLKNTSVLGAGGQTQDHFKLTSLPVLIRLPFRTTPIVLTSCLVDTKNNWAIIGRDALQQCQGVLYLPEAKRPPVIL


PBST    PIQAPAVLGLEHLPRPPEISQFPLNQNASRPCNTWSGRPWRQAISNPTPGQEITQYSQLKRPMEPGDSSTTCGPLTL

HAM1    PIQAPAVLGLEHLPRPPEISQFPLNQNASRPCNTWSGRPWRQAISNPTPGQEITQYSQLKKPMEPGDSSTTCGPLTL
HAM2    PIQAPAVLGLEHLPRPPEISQFPLNQNASRPCNTWSGRPWRQAISNPTPDQEITQYSQLKRPMEPGDSSTTCGPLTL
HAM3    PIQAPAVLGLEHLPRPPEISQFPLNQNASRPCNTWSGRPWRQAISNPTPDQEITQYSQLKRPMEPGDSSTTCGPLTL
HAM4    PIQAPAVLGLEHLPRPPEISQFPLNQNASRPCNTWSGRPWRQAISNPTPGQEITQYSQLKRPMEPGDSSTTCGPLTL
HAM5    PIQAPAVLGLEHLPRPPEISQFPLNQNASRPCNTWSGRPWRQAISNPTPDQEITQYSQLKRPMEPGDSSTTCGPLTL
HAM6    PIQAPAVLGLEHLPRPPEISQFPLNQNASRPCNTWSGRPWRQAISNPTPDQEITQYSQLKRPMEPGDSSTTCGPLTL
HAM7    PIQAPAVLGLEHLPRPPEISQFPLNQNASRPCNTWSGRPWRQAISNPTPGQEITQYSQLKKPMEPGDSSTTCGPLTL
HAM8    PIQAPAVLGLEHLPRPPEISQFPLNQNASRPCNTWSGRPWRQAISNPTPDQEITQYSQLKRPMEPGDSSTTCGPLTL
HAM9    PIQAPAVLGLEHLPRPPEISQFPLNQNASRPCNTWSGRPWRQAISNPTPDQEITQYSQLKRPMEPGDSSTTCGPLTL
HAM10   PIQAPAVLGLEHLPRPPEISQFPLNQNASRPCNTWSGRPWRQAISNPTPDQEITQYSQLKRPMEPGDSSTTCGPLTL
HAM11   PIQAPAVLGLEHLPRPPEISQFPLNQNASRPYNTWSGRPWRQAISNPTPDQEITQYSQLKRPMEPGDSSTTCGPLTL
HAM12   PIQAPAVLGLEHLPRPPEISQFPLNQNASRPCNTWSGRPWRQAISNPTPDQEITQYSQLKRPMEPGDSSTTCGPLTL

HAM1    PIQAPAVLGLEHLPRPPEISQFPLNQNASRPCNTWSGRPWRQAISNPTPDQEITQYSQLKRPMEPGDSSTTCGPLTL
HAM2    PIQAPAVLGLEHLPRPPEISQFPLNQNASRPCNTWSGRPWRQAISNPTPGQEITQYSQLKRPMEPGDSSTTCGPLTL
HAM3    PIQAPAVLGLEHLPRPPEISQFPLNQNASRPCNTWSGRPWRQAISNPTPGQEITQYSQLKRPMEPGDSSTTCGPLTL
HAM4    PIQAPAVLGLEHLPRPPEISQFPLNQNASRPCNTWSGRPWRQAISNPTPGQEITQYSQLKRPMEPGDSSTTCGPLTL
HAM5    PIQAPAVLGLEHLPRPPEISQFPLNQNASRPCNTWSGRPWRQAISNPTPGQEITQYSQLKRPMEPGDSSTTCGPLTL
HAM6    PIQAPAVLGLEHLPRPPEISQFPLNQNASRPCNTWSGRPWRQAISNPTPGQEITQYSQLKRPMEPGDSSTTCGPLTL
HAM7    PIQAPAVLGLEHLPRPPEISQFPLNQNASRPCNTWSGRPWRQAISNPTPGQEITQYSQLKRPMEPGDSSTTCGPLTL
HAM8    PIQAPAVLGLEHLPRPPEISQFPLNQNASRPCNTWSGRPWRQAISNPTPGQEITQYSQLKRPMEPGDSSTTCGPLTL
HAM9    PIQAPAVLGLEHLPRPPEISQFPLNQNASRPCNTWSGRPWRQAISNPTPGQEITQYSQLKRPMEPGDSSTTCGPLTL
HAM10   PIQAPAVLGLEHLPRPPEISQFPLNQNASRPCNTWSGRPWRQAISNPTPGQEITQYSQLKRPMEPGDSSTTCGPLTL

ACH     PIQAPAVLGLEHLPRPPEISQFPLNQNASRPCNTWSGRPWRQAISNPTPGQEITQYSQLKRPMEPGDSSTTCGPLTL
K30p    PIQAPAVLGLEHLPRPPEISQFPLNQNASRPCNTWSGRPWRQAISNPTPGQEITQYSQLKRPMEPGDSSTTCGPLTL
```
