# Supplementary material for: Characterization of HTLV-1 Infectious Molecular Clone Isolated from Patient with HAM/TSP and Immortalization of Human Primary T-Cell Lines
Source: Viruses. 2024 Nov 9;16(11):1755. doi: 10.3390/v16111755 (PMC11599126; doi:10.3390/v16111755)
Supplement: Supplementary file 1 [file viruses-16-01755-s001.zip › Supplemental S6 Pol align.pdf]

HTLV-1 POL ALIGNMENTS BETWEEN JAPANESE AND BRAZILIAN HAM/TSP ISOLATES (FULL GENOME SEQUENCE) AND OTHER HTLV-1 MOLECULAR CLONES

```
PBST     MQLAHILQPIRQAFPQCTILQYMDDILLASPSHEDLLLLSEATMASLISHGLPVSENKTQQTPGTIKFLGQIISPNHLTYDAVPTVPIRSRWAL

HAM1     MQLAHILQPIRQAFPQCTILQYMDDILLASPSHEDLLLLSEATMASLISHGLPVSENKTQQTPGTIKFLGQIISPNHLTYDAVPTVPIRSRWAL
HAM2     MQLAHILQPIRQAFPQCTILQYMDDILLASPSHEDLLLLSEATMASLISHGLPVSENKTQQTPGTIKFLGQIISPNHLTYDAVPTVPIRSRWAL
HAM3     MQLAHILQPIRQAFPQCTILQYMDDILLASPSHEDLLLLSEATMASLISHGLPVSENKTQQTPGTIKFLGQIISPNHLTYDAVPTVPIRSRWAL
HAM4     MQLAHILQPIRQAFPQCTILQYMDDILLASPSHEDLLLLSEATMASLISHGLPVSENKTQQTPGTIKFLGQIISPNHLTYDAVPTVPIRSRWAL
HAM5     MQLAHILQPIRQAFPQCTILQYMDDILLASPSHEDLLLLSEATMASLISHGLPVSENKTQQTPGTIKFLGQIISPNHLTYDAVPTVPIRSRWAL
HAM6     MQLAHILQPIRQAFPQCTILQYMDDILLASPSHEDLLLLSEATMASLISHGLPVSENKTQQTPGTIKFLGQIISPNHLTYDAVPTVPIRSRWAL
HAM7     MQLAHILQPIRQAFPQCTILQYMDDILLASPSHEDLLLLSEATMASLISHGLPVSENKTQQTPGTIKFLGQIISPNHLTYDAVPTVPIRSRWAL
HAM8     MQLAHILQPIRQAFPQCTILQYMDDILLASPSHEDLLLLSEATMASLISHGLPVSENKTQQTPGTIKFLGQIISPNHLTYDAVPTVPIRSRWAL
HAM9     MQLAHILQPIRQAFPQCTILQYMDDILLASPSHEDLLLLSEATMASLISHGLPVSENKTQQTPGTIKFLGQIISPNHLTYDAVPTVPIRSRWAL
HAM10    MQLAHILQPIRQAFPQCTILQYMDDILLASPSHEDLLLLSEATMASLISHGLPVSENKTQQTPGTIKFLGQIISPNHLTYDAVPTVPIRSRWAL
HAM11    MQLAHILQPIRQAFPQCTILQYMDDILLASPSHEDLLLLSEATMASLISHGLPVSENKTQQTPGTIKFLGQIISPNHLTYDAVPTVPIRSRWAL
HAM12    MQLAHILQPIRQAFPQCTILQYMDDILLASPSHEDLLLLSEATMASLISHGLPVSENKTQQTPGTIKFLGQIISPNHLTYDAVPTVPIRSRWAL

HAM1     MQLAHILQPIRQAFPQCTILQYMDDILLASPSHEDLLLLSEATMASLISHGLPVSENKTQQTPGTIKFLGQIISPNHLTYDAVPTVPIRSRWAL
HAM2     MQLAHILQPIRQAFPQCTILQYMDDILLASPSHEDLLLLSEATMASLISHGLPVSENKTQQTPGTIKFLGQIISPNHLTYDAVPTVPIRSRWAL
HAM3     MQLAHILQPIRQAFPQCTILQYMDDILLASPSHEDLLLLSEATMASLISHGLPVSENKTQQTPGTIKFLGQIISPNHLTYDAVPTVPIRSRWAL
HAM4     MQLAHILQPIRQAFPQCTILQYMDDILLASPSHEDLLLLSEATMASLISHGLPVSENKTQQTPGTIKFLGQIISPNHLTYDAVPTVPIRSRWAL
HAM5     MQLAHILQPIRQAFPQCTILQYMDDILLASPSHEDLLLLSEATMASLISHGLPVSENKTQQTPGTIKFLGQIISPNHLTYDAVPTVPIRSRWAL
HAM6     MQLAHILQPIRQAFPQCTILQYMDDILLASPSHEDLLLLSEATMASLISHGLPVSENKTQQTPGTIKFLGQIISPNHLTYDAVPTVPIRSRWAL
HAM7     MQLAHILQPIRQAFPQCTILQYMDDILLASPSHEDLLLLSEATMASLISHGLPVSENKTQQTPGTIKFLGQIISPNHLTYDAVPTVPIRSRWAL
HAM8     MQLAHILQPIRQAFPQCTILQYMDDILLASPSHEDLLLLSEATMASLISHGLPVSENKTQQTPGTIKFLGQIISPNHLTYDAVPTVPIRSRWAL
HAM9     MQLAHILQPIRQAFPQCTILQYMDDILLASPSHEDLLLLSEATMASLISHGLPVSENKTQQTPGTIKFLGQIISPNHLTYDAVPTVPIRSRWAL
HAM10    MQLAHILQPIRQAFPQCTILQYMDDILLASPSHEDLLLLSEATMASLISHGLPVSENKTQQTPGTIKFLGQIISPNHLTYDAVPTVPIRSRWAL

ACH      MQLAYILQPIRQAFPQCTILQYMDDILLASPSHEDLLLLSEATMASLISHGLPVSENKTQQTPGTIKFLGQIISPNHLTYDAVPTVPIRSRWAL
K30p     MQLAHILQPIRQAFPQCTILQYMDDILLASPSHEDLLLLSEATMASLISHGLPVSENKTQQTPGTIKFLGQIISPNHLTYDAVPTVPIRSRWAL


PBST     PELQALLGEIQWVSKGTPTLRQPLHSLYCALQRHTDPRDQIYLNPSQVQSLVQLRQALSQNCRSRLVQTLPLLGAIMLTLTGTTTVVFQSKQQW

HAM1     PELQALLGEIQWVSKGTPTLRQPLHSLYCALQRHTDPRDQIYLNPSQVQSLVQLRQALSQNCRSRLVQTLPLLGAIMLTLTGTTTVVFQSKQQW
HAM2     PELQALLGEIQWVSKGTPTLRQPLHSLYCALQRHTDPRDQIYLNPSQVQSLVQLRQALSQNCRSRLVQTLPLLGAIMLTLTGTTTVVFQSKQQW
HAM3     PELQALLGEIQWVSKGTPTLRQPLHSLYCALQRHTDPRDQIYLNPSQVQSLVQLRQALSQNCRSRLVQTLPLLGAIMLTLTGTTTVVFQSKQQW
HAM4     PELQALLGEIQWVSKGTPTLRQPLHSLYCALQRHTDPRDQIYLNPSQVQSLVQLRQALSQNCRSRLVQTLPLLGAIMLTLTGTTTVVFQSKQQW
HAM5     PELQALLGEIQWVSKGTPTLRQPLHSLYSALQRHTDPRDQIYLNPSQVQSLVQLRQALSQNCRSRLVQTLPLLGAIMLTLTGTTTVVFQSKQQW
HAM6     PELQALLGEIQWVSKGTPTLRQPLHSLYCALQRHTDPRDQIYLNPSQVQSLVQLRQALSQNCRSRLVQTLPLLGAIMLTLTGTTTVVFQSKQQW
HAM7     PELQALLGEIQWVSKGTPTLRQPLHSLYCALQRHTDPRDQIYLNPSQVQSLVQLRQALSQNCRSRLVQTLPLLGAIMLTLTGTTTVVFQSKQQW
HAM8     PELQALLGEIQWVSKGTPTLRQPLHSLYCALQRHTDPRDQIYLNPSQVQSLVQLRQALSQNCRSRLVQTLPLLGAIMLTLTGTTTVVFQSKQQW
HAM9     PELQALLGEIQWVSKGTPTLRQPLHSLYCALQRHTDPRDQIYLNPSQVQSLVQLRQALSQNCRSRLVQTLPLLGAIMLTLTGTTTVVFQSKQQW
HAM10    PELQALLGEIQWVSKGTPTLRQPLHSLYCALQRHTDPRDQIYLNPSQVQSLVQLRQALSQNCRSRLVQTLPLLGAIMLTLTGTTTVVFQSKQQW
HAM11    PELQALLGEIQWVSKGTPTLRQPLHSLYCALQRHTDPRDQIYLNPSQVQSLVQLRQALSQNCRSRLVQTLPLLGAIMLTLTGTTTVVFQSKQQW
HAM12    PELQALLGEIQWVSKGTPTLRQPLHSLYCALQRHTDPRDQIYLNPSQVQSLVQLRQALSQNCRSRLVQTLPLLGAIMLTLTGTTTVVFQSKQQW

HAM1     PELQALLGEIQWVSKGTPTLRQPLHSLYCALQRHTDPRDQIYLNPSQVRSLVQLRQALSQNCRSRLVQTLPLLGAIMLTLTGTTTVVFQSKQQW
HAM2     PELQALLGEIQWVSKGTPTLRQPLHSLYCALQRHTDPRDQIYLNPSQVQSLVQLRQALSQNCRSRLVQTLPLLGAIMLTLTGTTTVVFQSKQQW
HAM3     PELQALLGEIQWVSKGTPTLRQPLHSLYCALQRHTDPRDQIYLNPSQVQSLVQLRQALSQNCRSRLVQTLPLLGAIMLTLTGTTTVVFQSKQQW
HAM4     PELQALLGEIQWVSKGTPTLRQPLHSLYCALQRHTDPRDQIYLNPSQVQSLVQLRQALSQNCRSRLVQTLPLLGAIMLTLTGTTTVVFQSKQQW
HAM5     PELQALLGEIQWVSKGTPTLRQPLHSLYCALQRHTDPRDQIYLNPSQVQSLVQLRQALSQNCRSRLVQTLPLLGAIMLTLTGTTTVVFQSKQQW
HAM6     PELQALLGEIQWVSKGTPTLRQPLHSLYCALQRHTDPRDQIYLNPSQVQSLVQLRQALSQNCRSRLVQTLPLLGAIMLTLTGTTTVVFQSKQQW
HAM7     PELQALLGEIQWVSKGTPTLRQPLHSLYCALQRHTDPRDQIYLNPSQVQSLVQLRQALSQNCRSRLVQTLPLLGAIMLTLTGTTTVVFQSKQQW
HAM8     PELQALLGEIQWVSKGTPTLRQPLHSLYCALQRHTDPRDQIYLNPSQVQSLVQLRQALSQNCRSRLVQTLPLLGAIMLTLTGTTTVVFQSKQQW
HAM9     PELQALLGEIQWVSKGTPTLRQPLHSLYCALQRHTDPRDQIYLNPSQVQSLVQLRQALSQNCRSRLVQTLPLLGAIMLTLTGTTTVVFQSKQQW
HAM10    PELQALLGEIQWVSKGTPTLRQPLHSLYCALQRHTDPRDQIYLNPSQVQSLVQLRQALSQNCRSRLVQTLPLLGAIMLTLTGTTTVVFQSKQQW
HAM1     PELQALLGEIQWVSKGTPTLRQPLHSLYCALQRHTDPRDQIYLNPSQVQSLVQLRQALSQNCRSRLVQTLPLLGAIMLTLTGTTTVVFQSKQQW

ACH      PELQALLGEIQWVSKGTPTLRQPLHSLYCALQRHTDPRDQIYLNPSQVQSLVQLRQALSQNCRSRLVQTLPLLGAIMLTLTGTTTVVFQSKQQW
K30p     PELQALLGEIQWVSKGTPTLRQPLHSLYCALQRHTDPRDQIYLNPSQVQSLVQLRQALSQNCRSRLVQTLPLLGAIMLTLTGTTTVVFQSKQQW
```

**PBST**	PLVWLHAPLPHTSQCPWGQLLASAVLLLDKYTLQSYGLLCQTIHHNISTQTFNQFIQTSDHPSVPILLHHSHRFKNLCAQTGELWNTFLKTAAP
**HAM1**	PLVWLHAPLPHTSQCPWGQLLASAVLLLDKYTLQSYGLLCQTIHHNISTQTFNQFIQTSDHPSVPILLHHSHRFKNLGAQTGELWNTFLKTAAP
**HAM2**	PLVWLHAPLPHTSQCPWGQLLASAVLLLDKYTLQSYGLLCQTIHHNISTQTFNQFIQTSDHPSVPILLHHSHRFKNLGAQTGELWNTFLKTAAP
**HAM3**	PLVWLHAPLPHTSQCPWGQLLASAVLLLDKYTLQSYGLLCQTIHHNISTQTFNQFIQTSDHPSVPILLHHSHRFKNLGAQTGELWNTFLKTAAP
**HAM4**	PLVWLHAPLPHTSQCPWGQLLASAVLLLDKYTLQSYGLLCQTIHHNISTQTFNQFIQTSDHPSVPILLHHSHRFKNLGAQTGELWNTFLKTAAP
**HAM5**	PLVWLHAPLPHTSQCPWGQLLASAVLLLDKYTLQSYGLLCQTIHHNISTQTFNQFIQTSDHPSVPILLHHSHRFKNLGAQTGELWNTFLKTAAP
**HAM6**	PLVWLHAPLPHTSQCPWGQLLASAVLLLDKYTLQSYGLLCQTIHHNISTQTFNQFIQTSDHPSVPILLHHSHRFKNLGAQTGELWNTFLKTAAP
**HAM7**	PLVWLHAPLPHTSQCPWGQLLASAVLLLDKYTLQSYGLLCQTIHHNISTQTFNQFIQTSDHPSVPILLHHSHRFKNLGAQTGELWNTFLKTAAP
**HAM8**	PLVWLHAPLPHTSQCPWGQLLASAVLLLDKYTLQSYGLLCQTIHHNISTQTFNQFIQTSDHPSVPILLHHSHRFKNLGAQTGELWNTFLKTAAP
**HAM9**	PLVWLHAPLPHTSQCPWGQLLASAVLLLDKYTLQSYGLLCQTIHHNISTQTFNQFIQTSDHPSVPILLHHSHRFKNLGAQTGELWNTFLKTAAP
**HAM10**	PLVWLHAPLPHTSQCPWGQLLASAVLLLDKYTLQSYGLLCQTIHHNISTQTFNQFIQTSDHPSVPILLHHSHRFKNLGAQTGELWNTFLKTAAP
**HAM11**	PLVWLHAPLPHTSQCPWGQLLASAVLLLDKYTLQSYGLLCQTIHHNISTQTFNQFIQTSDHPSVPILLHHSHRFKNLGAQTGELWNTFLKTAAP
**HAM12**	PLVWLHAPLPHTSQCPWGQLLASAVLLLDKYTLQSYGLLCQTIHHNISTQTFNQFIQTSDHPSVPILLHHSHRFKNLGAQTGELWNTFLKTAAP
**HAM1**	PLVWLHAPLPHTSQCPWGQLLASAVLLLDKYTLQSYGLLCQTIHHNISTQTFNQFIQTSDHPSVPILLHHSHRFKNLGAQTGELWNTFLKTAAP
**HAM2**	PLVWLHAPLPHTSQCPWGQLLASAVLLLDKYTLQSYGLLCQTIHHNISTQTFNQFIQTSDHPSVPILLHHSHRFKNLGAQTGELWNTFLKTAAP
**HAM3**	PLVWLHAPLPHTSQCPWGQLLASAVLLLDKYTLQSYGLLCQTIHHNISTQTFNQFIQTSDHPSVPILLHHSHRFKNLGAQTGELWNTFLKTAAP
**HAM4**	PLVWLHAPLPHTSQCPWGQLLASAVLLLDKYTLQSYGLLCQTIHHNISTQTFNQFIQTSDHPSVPILLHHSHRFKNLGAQTGELWNTFLKTAAP
**HAM5**	PLVWLHAPLPHTSQCPWGQLLASAVLLLDKYTLQSYGLLCQTIHHNISTQTFNQFIQTSDHPSVPILLHHSHRFKNLGAQTGELWNTFLKTAAP
**HAM6**	PLVWLHAPLPHTSQCPWGQLLASAVLLLDKYTLQSYGLLCQTIHHNISTQTFNQFIQTSDHPSVPILLHHSHRFKNLGAQTGELWNTFLKTAAP
**HAM7**	PLVWLHAPLPHTSQCPWGQLLASAVLLLDKYTLQSYGLLCQTIHHNISTQTFNQFIQTSDHPSVPILLHHSHRFKNLGAQTGELWNTFLKTAAP
**HAM8**	PLVWLHAPLPHTSQCPWGQLLASAVLLLDKYTLQSYGLLCQTIHHNISTQTFNQFIQTSDHPSVPILLHHSHRFKNLGAQTGELWNTFLKTAAP
**HAM9**	PLVWLHAPLPHTSQCPWGQLLASAVLLLDKYTLQSYGLLCQTIHHNISTQTFNQFIQTSDHPSVPILLHHSHRFKNLGAQTGELWNTFLKTAAP
**HAM10**	PLVWLHAPLPHTSQCPWGQLLASAVLLLDKYTLQSYGLLCQTIHHNISTQTFNQFIQTSDHPSVPILLHHSHRFKNLGAQTGELWNTFLKTAAP
**ACH**	PLVWLHAPLPHTSQCPWGQLLASAVLLLDKYTLQSYGLLCQTIHHNISTQTFNQFIQTSDHPSVPILLHHSHRFKNLGAQTGELWNTFLKTAAP
**K30p**	PLVWLHAPLPHTSQCPWGQLLASAVLLLDKYTLQSYGLLCQTIHHNISTQTFNQFIQTSDHPSVPILLHHSHRFKNLGAQTGELWNTFLKTAAP

**PBST**	LAPVKALMPVFTLSPVILNTAPCLFSDGSTSRAAYILWDKHILSQRSFPLPPPHKSAQRAELLGLLHGLSSARSWRCLNIFLDSKYLYHYLRTL
**HAM1**	LAPVKALMPVFTLSPVIINTAPCLFSDGSTSRAAYILWDKHILSQRSFPLPPPHKSAQRAELLGLLHGLSSARSWRCLNIFLDSKYLYHYLRTL
**HAM2**	LAPVKALMPVFTLSPVIINTAPCLFSDGSTSRAAYILWDKHILSQRSFPLPPPHKSAQRAELLGLLHGLSSARSWRCLNIFLDSKYLYHYLRTL
**HAM3**	LAPVKALMPVFTLSPVIINTAPCLFSDGSTSRAAYILWDKHILSQRSFPLPPPHKSAQRAELLGLLHGLSSARSWRCLNIFLDSKYLYHYLRTL
**HAM4**	LAPVKALMPVFTLSPVIINTAPCLFSDGSTSRAAYILWDKHILSQRSFPLPPPHKSAQRAELLGLLHGLSSARSWHCLNIFLDSKYLYHYLRTL
**HAM5**	LAPVKALMPVFTLSPVIINTAPCLFSDGSTSRAAYILWDKHILSRRSFPLPPPHKSAQRAELLGLLHGLSSARSWRCLNIFLDSKYLYHYLRTL
**HAM6**	LAPVKALMPVFTLSPVIINTAPCLFSDGSTSRAAYILWDKHILSQRSFPLPPPHKSAQRAELLGLLHGLASARSWRCLNIFLDSKYLYHYLRTL
**HAM7**	LAPVKALMPVFTLSPVIINTAPCLFSDGSTSRAAYILWDKHILSQRSFPLPPPHKSAQRAELLGLLHGLSSARSWHCLNIFLDSKYLYHYLRTL
**HAM8**	LAPVKALMPVFTLSPVIINTAPCLFSDGSTSRAAYILWDKHILSRRSFPLPPPHKSAQRAELLGLLHGLSSARSWRCLNIFLDSKYLYHYLRTL
**HAM9**	LAPVKALMPVFTLSPVIINTAPCLFSDGSTSRAAYILWDKHILSQRSFPLPPPHKSAQRAELLGLLHGLSSARSWRCLNIFLDSKYLYHYLRTL
**HAM10**	LAPVKALMPVFTLSPVIINTAPCLFSDGSTSRAAYILWDKHILSQRSFPLPPPHKSAQRAELLGLLHGLSSARSWRCLNIFLDSKYLYHYLRTL
**HAM11**	LAPVKALMPVFTLSPVIINTAPCLFSDGSTSRAAYILWDKHILSQRSFPLPPPHKSAQRAELLGLLHGLSSARSWRCLNIFLDSKYLYHYLRTL
**HAM12**	LAPVKALMPVFTLSPVIINTAPCLFSDGSTSRAAYILWDKHILSQRSFPLPPPHKSAQRAELLGLLHGLSSARSWRCLNIFLDSKYLYHYLRTL
**HAM1**	LAPVKALMPVFTLSPVIINTAPCLFSDGSTSQAAYILWDKHILSQRSFPLPPPHKSAQRAELLGLLHGLSSARSWRCLNIFLDSKYLYHYLRTL
**HAM2**	LAPVKALMPVFTLSPVIINTAPCLFSDGSTSQAAYILWDKHILSQRSFPLPPPHKSAQRAELLGLLHGLSSARSWRCLNIFLDSKYLYHYLRTL
**HAM3**	LAPVKALMPVFTLSPVIINTAPCLFSDGSTSQAAYILWDKHILSQRSFPLPPPHKSAQRAELLGLLHGLSSARSWRCLNIFLDSKYLYHYLRTL
**HAM4**	LAPVKALMPVFTLSPVIINTAPCLFSDGSTSRAAYILWDKHILSQRSFPLPPPHKSAQRAELLGLLHGLSSARSWRCLNIFLDSKYLYHYLRTL
**HAM5**	LAPVKALMPVFTLSPVIINTAPCLFSDGSTSQAAYILWDKHILSQRSFPLPPPHKSAQRAELLGLLHGLSSARSWRCLNIFLDSKYLYHYLRTL
**HAM6**	LAPVKALMPVFTLSPVIINTAPCLFSDGSTSQAAYILWDKHILSQRSFPLPPPHKSAQRAELLGLLHGLSSARSWRCLNIFLDSKYLYHYLRTL
**HAM7**	LAPVKALMPVFTLSPVIINTAPCLFSDGSTSQAAYILWDKHILSQRSFPLPPPHKSAQRAELLGLLHGLSSARSWRCLNIFLDSKYLYHYLRTL
**HAM8**	LAPVKALMPVFTLSPVIINTAPCLFSDGSTSQAAYILWDKHILSQRSFPLPPPHKSAQRAELLGLLHGLSSARSWRCLNIFLDSKYLYHYLRTL
**HAM9**	LAPVKALMPVFTLSPVIINTAPCLFSDGSTSQAAYILWDKHILSQRSFPLPPPHKSAQRAELLGLLHGLSSARSWRCLNIFLDSKYLYHYLRTL
**HAM10**	LAPVKALMPVFTLSPVIINTAPCLFSDGSTSQAAYILWDKHILSQRSFPLPPPHKSAQRAELLGLLHGLSSARSWRCINIFLDSKYLYHYLRTL
**ACH**	LAPVKALMPVFTLSPVIINTAPCLFSDGSTSRAAYILWDKHILSQRSFPLPPPHKSAQRAELLGLLHGLSSARSWRCLNIFLDSKYLYHYLRTL
**K30p**	LAPVKALMPVFTLSPVIINTAPCLFSDGSTSRAAYILWDKHILSQRSFPLPPPHKSAQRAELLGLLHGLSSARSWRCLNIFLDSKYLYHYLRTL

**PBST**	ALGTFQGKSSQAPFQALLPRLLSRKVVYLHHVRSHTNLPDPISRLNALTDALLITPVLQLSPAELHSFTHCGQTALTLQGATTTEASNILRSCH
**HAM1**	ALGTFQGRSSQAPFQALLPRLLSHKVVYLHHVRSHTNLPDPISRLNALTDALLITPVLQLSPAELHSFTHCGQTALTLQGATTTEASNILRSCH
**HAM2**	ALGTFQGRSSQAPFQALLPRLLSRKVVYLHHVRSHTNLPDPISRLNALTDALLITPVLQLSPAELHSFTHCGQTALTLQGATTTEASNILRSCH
**HAM3**	ALGTFQGRSSQAPFQALLPRLLSRKVVYLHHVRSHTNLPDPISRLNALTDALLITPVLQLSPAELHSFTHCGQTALTLQGATTTEASNILRSCH
**HAM4**	ALGTFQGRSSQAPFQALLPRLLSRKVVYLHHVRSHTNLPDPISRLNALTDALLITPVLQLSPAELHSFTHCGQTALTLQGATTTEASNILRSCH
**HAM5**	ALGTFQGRSSQAPFQALLPRLLSRKVVYLHHVRSHTNLPDPISRLNALTDALLITPVLQLSPAELHSFTHCGQTALTLQGATTTEASNILRSCH
**HAM6**	ALGTFQGRSSQAPFQALLPRLLSRKVVYLHHVRSHTNLPDPISRLNALTDALLITPVLQLSPAELHSFTHCGQTALTLQGATTTEASNILRSCH
**HAM7**	ALGTFQGRSSQAPFQALLPRLLSRKVVYLHHVRSHTNLPDPISRLNALTDALLITPVLQLSPAELHSFTHCGQTALTLQGATTTEASNILRSCH
**HAM8**	ALGTFQGRSSQAPFQALLPRLLSRKVVYLHHVRSHTNLPDPISRLNALTDALLITPVLQLSPAELHSFTHCGQTALTLQGATTTEASNILRSCH
**HAM9**	ALGTFQGRSSQAPFQALLPRLLSRKVVYLHHVRSHTNLPDPISRLNALTDALLITPVLQLSPAELHSFTHCGQTALTLQGATTTEASNILRSCH
**HAM10**	ALGTFQGRSSQAPFQALLPRLLSRKVVYLHHVRSHTNLPDPISRLNALTDALLITPVLQLSPAELHSFTHCGQTALTLQGATTTEASNILRSCH
**HAM11**	ALGTFQGRSSQAPFQALLPRLLSRKVVYLHHVRSHTNLPDPISRLNALTDALLITPVLQLSPAELHSFTHCGQTALTLQGATTTEASNILRSCH
**HAM12**	ALGTFQGRSSQAPFQALLPRLLSRKVVYLHHVRSHTNLPDPISRLNALTDALLITPVLQLSPAELHSFTHCGQTALTLQGATTTEASNILRSCH

**HAM1**	ALGTFQGRSSQAPFQALLPRLLSHKVVYLHHVRSHTNLPDPISRLNALTDALLITPVLQLSPAELHSFTHCGQTALTLQGATTTEASNILRSCH
**HAM2**	ALGTFQGRSSQAPFQALLPRLLSRKVVYLHHVRSHTNLPDPISRLNALTDALLITPVLQLSPAELHSFTHCGQTALTLQGATTTEASNILRSCH
**HAM3**	ALGTFQGRSSQAPFQALLPRLLSRKVVYLHHVRSHTNLPDPISRLNALTDALLITPVLQLSPAELHSFTHCGQTALTLQGATTTEASNILRSCH
**HAM4**	ALGTFQGKSSQAPFQALLPRLLSRKVVYLHHVRSHTNLPDPISRLNALTDALLITPVLQLSPAELHSFTHCGQTALTLQGATTTEASNILRSCH
**HAM5**	ALGTFQGRSSQAPFQALLPRLLSRKVVYLHHVRSHTNLPDPISRLNALTDALLITPVLQLSPAELHSFTHCGQTALTLQGATTTEASNILRSCH
**HAM6**	ALGTFQGRSSQAPFQALLPRLLSRKVVYLHHVRSHTNLPDPISRLNALTDALLITPVLQLSPAELHSFTHCGQTALTLQGATTTEASNILRSCH
**HAM7**	ALGTFQGRSSQAPFQALLPRLLSRKVVYLHHVRSHTNLPDPISRLNALTDALLITPVLQLSPAELHSFTHCGQTALTLQGATTTEASNILRSCH
**HAM8**	ALGTFQGRSSQAPFQALLPRLLSRKVVYLHHVRSHTNLPDPISRLNALTDALLITPVLQLSPAELHSFTHCGQTALTLQGATTTEASNILRSCH
**HAM9**	ALGTFQGRSSQAPFQALLPRLLSRKVVYLHHVRSHTNLPDPISRLNALTDALLITPVLQLSPAELHSFTHCGQTALTLQGATTTEASNILRSCH
**HAM10**	ALGTFQGRSSQAPFQALLPRLLSRKVVYLHHVRSHTNLPDPISRLNALTDALLITPVLQLSPAELHSFTHCGQTALTLQGATTTEASNILRSCH

**ACH**	ALGTFQGRSSQAPFQALLPRLLSRKVVYLHHVRSHTNLPDPISRLNALTDALLITPVLQLSPAELHSFTHCGQTALTLQGATTTEASNILRSCH
**K30P**	ALGTFQGKSSQAPFQALLPRLLSRKVVYLHHVRSHTNLPDPISRLNALTDALLITPVLQLSPAELHSFTHCGQTALTLQGATTTEASNILRSCH

**PBST**	ACRKNNPQHQMPRGHIRRGLLPNHIWQGDITHFKYKNTLYRLHVWVDTFSGAISATQKRKETSSEAISSLLQAIAYLGKPSYINTDNGPAYISQ

**HAM1**	ACRKNNPQHQMPRGHIRRGLLPNHIWQGDITHFKYKNTLYRLHVWVDTFSGAISATQKRKETSSEAISSLLQAIAYLGKPSYINTDNGPAYISQ
**HAM2**	ACRKNNPQHQMPRGHIRRGLLPNHIWQGDITHFKYKNTLYRLHVWVDTFSGAISATQKRKETSSEAISSLLQAIAYLGKPSYINTDNGPAYISQ
**HAM3**	ACRKNNPQHQMPRGHIRRGLLPNHIWQGDITHFKYKNTLYRLHVWVDTFSGAISATQKRKETSSEAISSLLQAIAYLGKPSYINTDNGPAYISQ
**HAM4**	ACRKNNPQHQMPRGHIRRGLLPNHIWQGDITHFKYKNTLYRLHVWVDTFSGAISATQKRKETSSEAISSLLQAIAYLGKPSYINTDNGPAYISQ
**HAM5**	ACRKNNPQHQMPRGHIRRGLLPNHIWQGDITHFKYKNTLYRLHVWVDTFSGAISATQKRKETSSEAISSLLQAIAYLGKPSYINTDNGPAYISQ
**HAM6**	ACRKNNPQHQMPRGHIRRGLLPNHIWQGDITHFKYKNTLYRLHVWVDTFSGAISATQKRKETSSEAISSLLQAIAYLGKPSYINTDNGPAYISQ
**HAM7**	ACRKNNPQHQMPRGHIRRGLLPNHIWQGDITHFKYKNTLYRLHVWVDTFSGAISATQKRKETSSEAISSLLQAIAYLGKPSYINTDNGPAYISQ
**HAM8**	ACRKNNPQHQMPRGHIRRGLLPNHIWQGDITHFKYKNTLYRLHVWVDTFSGAISATQKRKETSSEAISSLLQAIAYLGKPSYINTDNGPAYISQ
**HAM9**	ACRKNNPQHQMPRGHIRRGLLPNHIWQGDITHFKYKNTLYRLHVWVDTFSGAISATQKRKETSSEAISSLLQAIAYLGKPSYINTDNGPAYISQ
**HAM10**	ACRKNNPQHQMPRGHIRRGLLPNHIWQGDITHFKYKNTLYRLHVWVDTFSGAISATQKRKETSSEAISSLLQAIAYLGKPSYINTDNGPAYISQ
**HAM11**	ACRKNNPQHQMPRGHIRRGLLPNHIWQGDITHFKYKNTLYRLHVWVDTFSGAISATQKRKETSSEAISSLLQAIAYLGKPSYINTDNGPAYISQ
**HAM12**	ACRKNNPQHQMPRGHIRRGLLPNHIWQGDITHFKYKNTLYRLHVWVDTFSGAISATQKRKETSSEAISSLLQAIAYLGKPSYINTDNGPAYISQ

**HAM1**	ACRKNNPQHQMPRGHIRRGLLPNHIWQGDITHFKYKNTLYRLHVWVDTFSGAISATQKRKETSSEAISSLLQAIAYLGKPSYINTDNGPAYISQ
**HAM2**	ACRKNDPQHQMPRGHIRRGLLPNHIWQGDITHFKYKNTLYRLHVWVDTFSGAISATQKRKETSSEAISSLLQAIAYLGKPSYINTDNGPAYISQ
**HAM3**	ACRKNNPQHQMPRGHIRRGLLPNHIWQGDITHFKYKNTLYRLHVWVDTFSGAISATQKRKETSSEAISSLLQAIAYLGKPSYINTDNGPAYISQ
**HAM4**	ACRKNNPQHQMPRGHIRRGLLPNHIWQGDITHFKYKNTLYRLHVWVDTFSGAISATQKRKETSSEAISSLLQAIAYLGKPSYINTDNGPAYISQ
**HAM5**	ACRKNNPQHQMPRGHIRRGLLPNHIWQGDITHFKYKNTLYRLHVWVDTFSGAISATQKRKETSSEAISSLLQAIAYLGKPSYINTDNGPAYISQ
**HAM6**	ACRKNNPQHQMPRGHIRRGLLPNHIWQGDITHFKYKNTLYRLHVWVDTFSGAISATQKRKETSSEAISSLLQAIAYLGKPSYINTDNGPAYISQ
**HAM7**	ACRKNNPQHQMPRGHIRRGLLPNHIWQGDITHFKYKNTLYRLHVWVDTFSGAISATQKRKETSSEAISSLLQAIAYLGKPSYINTDNGPAYISQ
**HAM8**	ACRKNNPQHQMPRGHIRRGLLPNHIWQGDITHFKYKNTLYRLHVWVDTFSGAISATQKRKETSSEAISSLLQAIAYLGKPSYINTDNGPAYISQ
**HAM9**	ACRKNNPQHQMPRGHIRRGLLPNHIWQGDITHFKYKNTLYRLHVWVDTFSGAISATQKRKETSSEAISSLLQAIAYLGKPSYINTDNGPAYISQ
**HAM10**	ACRKNNPQHQMPRGHIRRGLLPNHIWQGDITHFKYKNTLYRLHVWVDTFSGAISATQKRKETSSEAISSLLQAIAYLGKPSYINTDNGPAYISQ

**ACH**	ACRKNNPQHQMPRGHIRRGLLPNHIWQGDITHFKYKNTLYRLHVWVDTFSGAISATQKRKETSSEAISSLLQAIAYLGKPSYINTDNGPAYISQ
**K30p**	ACRKNNPQHQMPRGHIRRGLLPNHIWQGDITHFKYKNTLYRLHVWVDTFSGAISATQKRKETSSEAISSLLQAIAYLGKPSYINTDNGPAYISQ

**PBST**	DFLNMCTSLAIRHTTHVPYNPTSSGLVERSNGILKTLLYKYFTDKPDLPMDNALSIALWTINHLNVLTNCHKTRWQLHHSPRLQPIPETHSLSN

**HAM1**	DFLNMCTSLAIRHTTHVPYNPTSSGLVERSNGILKTLLYKYFTDKPDLPMDNALSIALWTINHLNVLTNCHKTRWQLHHSPRLQPIPETHSLSN
**HAM2**	DFLNMCTSLAIRHTTHVPYNPTSSGLVERSNGILKTLLYKYFTDKPDLPMDNALSIALWTINHLNVLTNCHKTRWQLHHSPRLQPIPETHSLSN
**HAM3**	DFLNMCTSLAIRHTTHVPYNPTSSGLVERSNGILKTLLYKYFTDKPDLPMDNALSIALWTINHLNVLTNCHKTRWQLHHSPRLQPIPETHSLSN
**HAM4**	DFLNMCTSLAIRHTTHVPYNPTSSGLVERSNGILKTLLYKYFTDKPDLPMDNALSIALWTINHLNVLTNCHKTRWQLHHSPRLQPIPETHSLSN
**HAM5**	DFLNMCTSLAIRHTTHVPYNPTSSGLVERSNGILKTLLYKYFTDKPDLPMDNALSIALWTINHLNVLTNCHKTRWQLHHSPRLQPIPETHSLSN
**HAM6**	DFLNMCTSLAIRHTTHVPYNPTSSGLVERSNGILKTLLYKYFTDKPDLPMDNALSIALWTINHLNVLTNCHKTRWQLHHSPRLQPIPETHSLSN
**HAM7**	DFLNMCTSLAIRHTTHVPYNPTSSGLVERSNGILKTLLYKYFTDKPDLPMDNALSIALWTINHLNVLTNCHKTRWQLHHSPRLQPIPETHSLSN
**HAM8**	DFLNMCTSLAIRHTTHVPYNPTSSGLVERSNGILKTLLYKYFTDKPDLPMDNALSIALWTINHLNVLTNCHKTRWQLHHSPRLQPIPETHSLSN
**HAM9**	DFLNMCTSLAIRHTTHVPYNPTSSGLVERSNGILKTLLYKYFTDKPDLPMDNALSIALWTINHLNVLTNCHKTRWQLHHSPRLQPIPETHSLSN
**HAM10**	DFLNMCTSLAIRHTTHVPYNPTSSGLVERSNGILKTLLYKYFTDKPDLPMDNALSIALWTINHLNVLTNCHKTRWQLHHSPRLQPIPETHSLSN
**HAM11**	DFLNMCTSLAIRHTTHVPYNPTSSGLVERSNGILKTLLYKYFTDKPDLPMDNALSIALWTINHLNVLTNCHKTRWQLHHSPRLQPIPETHSLSN
**HAM12**	DFLNMCTSLAIRHTTHVPYNPTSSGLVERSNGILKTLLYKYFTDKPDLPIDNALSIALWTINHLNVLTNCHKTRWQLHHSPRLQPIPETHSLSN

**HAM1**	DFLNMCTSLAIRHTTHVPYNPTSSGLVERSNGILKTLLYKYFTDKPDLPMDNALSIALWTINHLNVLTNCHKTRWQLHHSPRLQPIPETHSLSN
**HAM2**	DFLNMCTSLAIRHTTHVPYNPTSSGLVERSNGILKTLLYKYFTDKPDLPMDNALSIALWTINHLNVLTNCHKTRWQLHHSPRLQPIPETHSLSN
**HAM3**	DFLNMCTSLAIRHTTHVPYNPTSSGLVERSNGILKTLLYKYFTDKPDLPMDNALSIALWTINHLNALTNCHKTRWQLHHSPRLQPIPETHSLSN
**HAM4**	DFLNMCTSLAIRHTTHVPYNPTSSGLVERSNGILKTLLYKYFTDKPDLPMDNALSIALWTINHLNVLTNCHKTRWQLHHSPRLQPIPETHSLSN
**HAM5**	DFLNMCTSLAIRHTTHVPYNPTSSGLVERSNGILKTLLYKYFTDKPDLPMDNALSIALWTINHLNVLTNCHKTRWQLHHSPRLQPIPETHSLSN
**HAM6**	DFLNMCTSLAIRHTTHVPYNPTSSGLVERSNGILKTLLYKYFTDKPDLPMDNALSIALWTINHLNVLTNCHKTRWQLHHSPRLQPIPETHSLSN
**HAM7**	DFLNMCTSLAIRHTTHVPYNPTSSGLVERSNGILKTLLYKYFTDKPDLPMDNALSIALWTINHLNVLTNCHKTRWQLHHSPRLQPIPETHSLSN
**HAM8**	DFLNMCTSLAIRHTTHVPYNPTSSGLVERSNGILKTLLYKYFTDKPDLPMDNALSIALWTINHLNVLTNCHKTRWQLHHSPRLQPIPETHSLSN
**HAM9**	DFLNMCTSLAIRHTTHVPYNPTSSGLVERSNGILKTLLYKYFTDKPDLPMDNALSIALWTINHLNVLTNCHKTRWQLHHSPRLQPIPETHSLSN
**HAM10**	DFLNMCTSLAIRHTTHVPYNPTSSGLVERSNGILKTLLYKYFTDKPDLPMDNALSIALWTINHLNVLTNCHKTRWQLHHSPRLQPIPETHSLSN

**ACH**	DFLNMCTSLAIRHTTHVPYNPTSSGLVERSNGILKTLLYKYFTDKPDLPMDNALSIALWTINHLNVLTNCHKTRWQLHHSPRLQPIPETHSLSN
**K30p**	DFLNMCTSLAIRHTTHVPYNPTSSGLVERSNGILKTLLYKYFTDKPDLPMDNALSIALWTINHLNVLTNCHKTRWQLHHSPRLQPIPETHSLSN

**PBST**	KQTHWYYFKLPGLNSRQWKGPQEALQEAAGAALIPVSASSAQWIPWRLLKRAACPRPVGGPADPKEKDHQHHG
**HAM1**	KQTHWYYFKLPGLNSRQWKGPQEALQEAAGAALIPVSASSAQWIPWRLLKRAACPRPVGGPADPKEKDHQHHG
**HAM2**	KQTHWYYFKLPGLNSRQWKGPQEALQEAAGAALIPVSASSAQWIPWRLLKRAACPRPVGGPADPKEKDHQHHG
**HAM3**	KQTHWYYFKLPGLNSRQWKGPQEALQEAAGAALIPVSASSAQWIPWRLLKRAACPRPVGGPADPKEKDHQHHG
**HAM4**	KQTHWYYFKLPGLNSRQWKGPQEALQEAAGAALIPVSASSAQWIPWRLLKRAACPRPVGGPADPKEKDHQHHG
**HAM5**	KQTHWYYFKLPGLNSRQWKGPQEALQEAAGAALIPVSASSAQWIPWRLLKRAACPRPVGGPADPKEKDHQHHG
**HAM6**	KQTHWYYFKLPGLNSRQWKGPQEALQEAAGAALIPVSASSAQWIPWRLLKRAACPRPVGGPADPKEKDHQHHG
**HAM7**	KQTHWYYFKLPGLNSRQWKGPQEALQEAAGAALIPVSASSAQWIPWRLLKRAACPRPVGGPADPKEKDHQHHG
**HAM8**	KQTHWYYFKLPGLNSRQWKGPQEALQEAAGAALIPVSASSAQWIPWRLLKRAACPRPVGGPADPKEKDHQHHG
**HAM9**	KQTHWYYFKLPGLNSRQWKGPQEALQEAAGAALIPVSASSAQWIPWRLLKRAACPRPVGGPADPKEKDHQHHG
**HAM10**	KQTHWYYFKLPGLNSRQWKGPQEALQEAAGAALIPVSASSAQWIPWRLLKRAACPRPVGGPADPKEKDHQHHG
**HAM11**	KQTHWYYFKLPGLNSRQWKGPQEALQEAAGAALIPVSASSAQWIPWRLLKRAACPRPVGGPADPKEKDHQHHG
**HAM12**	KQTHWYYFKLPGLNSRQWKGPQEALQEAAGAALIPVSASSAQWIPWRLLKRAACPRPVGGPADPKEKDHQHHG
**HAM1**	KQTHWYYFKLPGLNSRQWKGPQEALQEAAGAALIPVSASSAQWIPWRLLKRAACPRPVGGPADPKEKDHQHHG
**HAM2**	KQTHWYYFKLPGLNSRQWKGPQEALQEAAGAALIPVSASSAQWIPWRLLKRAACPRPVGGPADPKEKDHQHHG
**HAM3**	KQTHWYYFKLPGLNSRQWKGPQEALQEAAGAALIPVSASSAQWIPWRLLKRAACPRPVGGPADPKEKDHQHHG
**HAM4**	KQTHWYYFKLPGLNSRQWKGPQEALQEAAGAALIPVSASSAQWIPWRLLKRAACPRPVGGPADPKEKDHQHHG
**HAM5**	KQTHWYYFKLPGLNSRQWKGPQEALQEAAGAALIPVSASSAQWIPWRLLKRAACPRPVGGPADPKEKDHQHHG
**HAM6**	KQTHWYYFKLPGLNSRQWKGPQEALQEAAGAALIPVSASSAQWIPWRLLKRAACPRPVGGPADPKEKDHQHHG
**HAM7**	KQTHWYYFKLPGLNSRQWKGPQEALQEAAGAALIPVSASSAQWIPWRLLKRAACPRPVGGPADPKEKDHQHHG
**HAM8**	KQTHWYYFKLPGLNSRQWKGPQEALQEAAGAALIPVSASSAQWIPWRLLKRAACPRPVGGPADPKEKDHQHHG
**HAM9**	KQTHWYYFKLPGLNSRQWKGPQEALQEAAGAALIPVSASSAQWIPWRLLKRAACPRPVGGPADPKEKDHQHHG
**HAM10**	KQTHWYYFKLPGLNSRQWKGPQEALQEAAGAALIPVSASSAQWIPWRLLKRAACPRPVGGPADPKEKDHQHHG
**ACH**	KQTHWYYFKLPGLNSRQWKGPQEALQEAAGAALIPVSASSAQWIPWRLLKRAACPRPVGGPADPKEKDLQHHG
**K30p**	KQTHWYYFKLPGLNSRQWKGPQEALQEAAGAALIPVSASSAQWIPWRLLKRAACPRPVGGPADPKEKDHQHHG
