# Supplementary material for: Characterization of HTLV-1 Infectious Molecular Clone Isolated from Patient with HAM/TSP and Immortalization of Human Primary T-Cell Lines
Source: Viruses. 2024 Nov 9;16(11):1755. doi: 10.3390/v16111755 (PMC11599126; doi:10.3390/v16111755)
Supplement: Supplementary file 1 [file viruses-16-01755-s001.zip › Supplemental S7 Env align.pdf]

HTLV-1 GP62 ENV ALIGNMENTS BETWEEN JAPANESE AND BRAZILIAN HAM/TSP ISOLATES (FULL GENOME SEQUENCE) AND OTHER HTLV-1 MOLECULAR CLONES

**ENV**	MGKFLATLILFFQFCPLILGDYSPSCCTLTIGVSSYHSEPCNPAQPVCSWTLDLLALSADQALQPPCPNLVGYSSYHATYSLYLFPHWIKKPNRNGGGY

**HAM1**	MGKFLATLILFFQFCPLILGDYSPSCCTLTIGVSSYHSKPCNPAQPVCSWTLDLLALSADQALQPPCPNLVSYSSYHATYSLYLFPHWIKKPNRNGGGY
**HAM2**	MGKFLATLILFFQFCPLILGDYSPSCCTLTIGVSSYHSKPCNPAQPVCSWTLDLPALSTDQALQPPCPNLVSYSSYHATYSLYLFPHWIKKPNRNGGGY
**HAM3**	MGKFLATLILFFQLCPLILGDYSPSCCTLTIGVSSYHSKPCNPAQPVCSWTLDLPALSADQALQPPCPNLVSYSSYHATYSLYLFPHWIKKPNRNGGGY
**HAM4**	MGKFLATLILFFQFCPLILGDYSPSCCTLTIGVSSYHSKPCNPAQPVCSWTLDLLALSADQALQPPCPNLVGYSSYHATYSLYLFPHWIKKPNRNGGGY
**HAM5**	MGKFLATLILFFQSCPLILGDYSPSCCTLTIGVSSYHSKPCNPAQPVCSWTLDLPALSADQALQPPCPNLVSYSSYHATYSLYLFPHWIKKPNRNGGGY
**HAM6**	MGKFLATLILFFQFCPLILGDYSPSCCTLTIGVSSYHSKPCNPAQPVCSWTLDLPALSADQALQPPCPNLVSYSSYHATYSLYLFPHWIKKPNRNGGGY
**HAM7**	MGKFLATLILFFQFCPLILGDYSPSCCTLTIGVSSYHSKPCNPAQPVCSWTLDLLALSADQALQPPCPNLVSYSSYHATYSLYLFPHWIKKPNRNGGGY
**HAM8**	MGKFLATLILFFQFCPLILGDYSPSCCTLTIGVSSYHSKPCNPAQPVCSWTLDLLALSADQALQPPCPNLVSYSSYHATYSLYLFPHWIKKPNRNGGGY
**HAM9**	MGKFLATLILFFQFCPLILGDYSPSCCTLTIGVSSYHSKPCNPAQPVCSWTLDLLALSADQALQPPCPNLVSYSSYHATYSLYLFPHWIKKPNRNGGGY
**HAM10**	MGKFLATLILFFQFCPLILGDYSPSCCTLTIGVSSYHSKPCNPAQPVCSWTLDLLALSADQALQPPCPNLVGYSSYHATYSLYLFPHWIKKPNRNGGGY
**HAM11**	MGKFLATLILFFQFCPLILGDYSPSCCTLTIGVSSYHSKPCNPAQPVCSWTLDLLALSADQALQPPCPNLVSYSSYHATYSLYLFPHWIKKPNRNGGGY
**HAM12**	MGKFLATLILFFQFCPLILGDYSPSCCTLTIGVSSYHSKPCNPAQPVCSWTLDLPALSADQALQPPCPNLVSYSSYHATYSLYLFPHWIKKPNRNGGGY

**HAM1**	MGKFLATLILFFQFCPLILGDYSPSCCTLTIGVSSYHSKPCNPAQPVCSWTLDLLALSADQALQPPCPNLVSYSSYHATYSLYLFPHWIKKPNRNGGGY
**HAM2**	MGKFLATLILFFQFCPLILGDYSPSCCTLTIGVSSYHSKPCNPAQPVCSWTLDLLALSADQALHPPCPNLVGYSSYHATYSLYLFPHWIKKPNRNGGGY
**HAM3**	MGKFLATLILFFQFCPLILGDYSPSCCTLTIGVSSYHSKPCNPAQPVCSWTLDLLALSADQALHPPCPNLVSYSSYHATYSLYLFPPRIKKSNRNGGGY
**HAM4**	MGKFLATLILFFQLCPLILGDYSPSCCTLTIGVSSYHSKPCNPAQPVCSWTLDLLALSADQALQPPCPNLVGYSSYHATYSLYLFPHWIKKPNRNGGGY
**HAM5**	MGKFLATLILFFQFCPLILGDYSPSCCTLTIGVSSYHSKPCNPAQPVCSWTLDLLALSADQALQPPCPNLVGYSSYHATYSLYLFPHWIKKPNRNGGGY
**HAM6**	MGKFLATLILFFQFCPLILGDYSPSCCTLTIGVSSYHSKPCNPAQPVCSWTLDLLALSADQALQPPCPNLVSYSSYHATYSLYLFPHWIKKPNRNGGGY
**HAM7**	MGKFLATLILFFQFCPLILGDYSPSCCTLTIGVSSYHSKPCNPAQPVCSWTLDLLALSADQALQPPCPNLVSYSSYHATYSLYLFPHWIKKPNRNGGGY
**HAM8**	MGKFLATLILFFQFCPLILGDYSPSCCTLTIGVSSYHSKPCNPAQPVCSWTLDLLALSADQALHPPCPNLVGYSSYHATYSLYLFPHWIKKPNRNGGGY
**HAM9**	MGKFLATLILFFQFCPLILGDYSPSCCTLTIGVSSYHSKPCNPAQPVCSWTLDLLALSADQALQPPCPNLVGYSSYHATYSLYLFPHWIKKPNQNGGGY
**HAM10**	MGKFLATLILFFQFCPLILGDYSPSCCTLTIGVSSYHSKPCNPAQPVCSWTLDLLALSADQALQPPCPNLVGYSSYHATYSLYLFPHWIKKPNRNGGGY

**ACH**	MGKFLATLILFFQFCPLILGDYSPSCCTLTIGVSSYHSKPCNPAQPVCSWTLDLLALSADQALQPPCPNLVGYSSYYATYSLYLFPHWIKKPNRNGGGY
**K30p**	MGKFLATLILFFQFCPLILGDYSPSCCTLTIGVSSYHSKPCNPAQPVCSWTLDLLALSADQALQPPCPNLVGYSSYHATYSLYLFPHWIKKPNRNGGGY

	YSASYSDPCSLKCPYLGCQSWTCPYTGAVSSPYWKFQQDVNFTQEVSRLNINLHFSKCGFPFSLLVDAPGYDPIWFLNTEPSQLPPTAPPLLPHSNLDH

**HAM1**	YSASYSDPCSLKCPYLGCQSWTCPYTGAVSSPYWKFQQDVNFTQEVSRLNINLHFSKCGFPFSLLVDAPGYDPIWFLNTEPSQLPPTAPPLLPHSNLDH
**HAM2**	YSASYSDPCSLKCPYLGCQSWTCPYTGAVSSPYWKFQQDVNFTQEVSRLNINLHFSKCGFPFSLLVDAPGYDPIWFLNTEPSQLPPTAPPLLPHSNLDH
**HAM3**	YSASYSDPCSLKCPYLGCQSWTCPYTGAVSSPYWKFQQDVNFTQEVSRLNINLHFSKCGFPFSLLVDAPGYDPIWFLNTEPSQLPPTAPPLLPHSNLDH
**HAM4**	YSASYSDPCSLKCPYLGCQSWTCPYTGAVSSPYWKFQQDVNFTQEVSRLNINLHFSKCGFPFSLLVDAPGYDPIWFLNTEPSQLPPTAPPLLPHSNLDH
**HAM5**	YSASYSDPCSLKCPYLGCQSWTCPYTGAVSSPYWKFQQDVNFTQEVSRLNINLHFSKCGFPFSLLVDAPGYDPIWFLNTEPSQLPPTAPPLLPHSNLDH
**HAM6**	YSASYSDPCSLKCPYLGCQSWTCPYTGAVSSPYWKFQHDVNFTQEVSRLNINLHFSKCGFPFSLLVDAPGYDPIWFLNTEPSQLPPTAPPLLPHSNLDH
**HAM7**	YSASYSDPCSLKCPYLGCQSWTCPYTGAVSSPYWKFQQDVNFTQEVSRLNINLHFSKCGFPFSLLVDAPGYDPIWFLNTEPSQLPPTAPPLLPHSNLDH
**HAM8**	YSASYSDPCSLKCPYLGCQSWTCPYTGAVSSPYWKFQQDVNFTQEVSRLNINLHFSKCGFPFSLLVDAPGYDPIWFLNTEPSQLPPTAPPLLPHSNLDH
**HAM9**	YSASYSDPCSLKCPYLGCQSWTCPYTGAVSSPYWKFQQDVNFTQEVSRLNINLHFSKCGFPFSLLVDAPGYDPIWFLNTEPSQLPPTAPPLLPHSNLDH
**HAM10**	YSASYSDPCSLKCPYLGCQSWTCPYTGAVSSPYWKFQQDVNFTQEVSRLNINLHFSKCGFPFSLLVDAPGYDPIWFLNTEPSQLPPTAPPLLPHSNLDH
**HAM11**	YSASYSDPCSLKCPYLGCQSWTCPYTGAVSSPYWKFQQDVNFTQEVSRLNINLHFSKCGFPFSLLVDAPGYDPIWFLNTEPSQLPPTAPPLLPHSNLDH
**HAM12**	YSASYSDPCSLKCPYLGCQSWTCPYTGAVSSPYWKFQQDVNFTQEVSRLNINLHFSKCGFPFSLLVDAPGYDPIWFLNTEPSQLPPTAPPLLPHSNLDH

**HAM1**	YSASYSDPCSLKCPYLGCQSWTCPYTGAVSSPYWKFQQDVNFTQEVSRLNINLHFSKCGFPFSLLVDAPGYDPIWFLNTEPSQLPPTAPPLLPHSNLDH
**HAM2**	YSASYSDPCSLKCPYLGCQSWTCPYTGAVSSPYWKFQQDVNFTQEVSRLNINLHFSKCGFPFSLLVDAPGYDPIWFLNTEPSQLPPTAPPLLPHSNLDH
**HAM3**	YSASYSDPCSLKCPYLGCQSWTCPYTGAVSSPYWKFQQDVNFTQEVSRLNINLHFSKCGFPFSLLVDAPGYDPIWFLNTEPSQLPPTAPPLLPHSNLDH
**HAM4**	YSASYSDPCSLKCPYLGCQSWTCPYTGAVSSPYWKFQQDVNFTQEVSRLNINLHFSKCGFPFSLLVDAPGYDPIWFLNTEPSQLPPTAPPLLPHSNLDH
**HAM5**	YSASYSDPCSLKCPYLGCQSWTCPYTGAVSSPYWKFQQDVNFTQEVSRLNINLHFSKCGFPFSLLVDAPGYDPIWFLNTEPSQLPPTAPPLLPHSNLDH
**HAM6**	YSASYSDPCSLKCPYLGCQSWTCPYTGAVSSPYWKFQQDVNFTQEVSRLNINLHFSKCGFPFSLLVDAPGYDPIWFLNTEPSQLPPTAPPLLPHSNLDH
**HAM7**	YSASYSDPCSLKCPYLGCQSWTCPYTGAVSSPYWKFQQDVNFTQEVSRLNINLHFSKCGFPFSLLVDAPGYDPIWFLNTEPSQLPPTAPPLLPHSNLDH
**HAM8**	YSASYSDPCSLKCPYLGCQSWTCPYTGAVSSPYWKFQQDVNFTQEVSRLNINLHFSKCGFPFSLLVDAPGYDPIWFLNTEPSQLPPTAPPLLPHSNLDH
**HAM9**	YSASYSDPCSLKCPYLGCQSWTCPYTGAVSSPYWKFQQDVNFTQEVSRLNINLHFSKCGFPFSLLVDAPGYDPIWFLNTEPSQLPPTAPPLLPHSNLDH
**HAM10**	YSASYSDPCSLKCPYLGCQSWTCPYTGAVSSPYWKFQQDVNFTQEVSRLNINLHFSKCGFPFSLLVDAPGYDPIWFLNTEPSQLPPTAPPLLPHSNLDH

**ACH**	YSASYSDPCSLKCPYLGCQSWTCPYTGAVSSPYWKFQQDVNFTQEVSRLNINLHFSKCGFPFSLLVDAPGYDPIWFLNTEPSQLPPTAPPLLPHSNLDH
**K30p**	YSASYSDPCSLKCPYLGCQSWTCPYTGAVSSPYWKFQQDVNFTQEVSRLNINLHFSKCGFPFSLLVDAPGYDPIWFLNTEPSQLPPTAPPLLPHSNLDH

	ILEPSIPWKSKLLTLVQLTLQSTNYTCIVCIDRASLSTWHVLYSPNVSVPSSSSTPLLYPSLALPAPHLTLPFNWTHCFDPQIQAIVSSPCHNSLILPP

**HAM1**	ILEPSIPWKSKLLTLVQLTLQSTNYTCIVCIDRASLSTWHVLYSPNVSVPSSSSTPLLYPSLALPAPHLTLPFNWTHCFDPQIQAIVSSPCHNSLILPP
**HAM2**	ILEPSIPWKSKLLTLVQLTLQSTNYTCIVCIDRASLSTWHVLYSPNVSVPSSSSTPLLYPSLALPAPHLTLPFNWTHCFDPQIQAIVSSPCHNSLILPP
**HAM3**	ILEPSIPWKSKLLTLVQLTLQSTNYTCIVCIDRASLSTWHVLYSPNVSVPSSSSTPLLYPSLALPAPHLTLPFNWTHCFDPQIQAIVSSPCHNSLILPP
**HAM4**	ILEPSIPWKSKLLTLVQLTLQSTNYTCIVCIDRASLSTWHVLYSPNVSVPSSSSTPLLYPSLALPAPHLTLPFNWTHCFDPQIQAIVSSPCHNSLILPP
**HAM5**	ILEPSIPWKSKLLTLVQLTLQSTNYTCIVCIDRASLSTWHVLYSPNVSVPSSSSTPLLYPSLALPAPHLTLPFNWTHCFDPQIQAIVSSPCHNSLILPP
**HAM6**	ILEPSIPWKSKLLTLVQLTLQSTNYTCIVCIDRASLSTWHVLYSPNISVPSSSSTPLLYPSLALPAPHLTLPFNWTHCFDPQIQAIVSSPCHNSLILPP
**HAM7**	ILEPSIPWKSKLLTLVQLTLQSTNYTCIVCIDRASLSTWHVLYSPNVSVPSSSSTPLLYPSLALPAPHLTLPFNWTHCFDPQIQAIVSSPCHNSLILPP
**HAM8**	ILEPSIPWKSKLLTLVQLTLQSTNYTCIVCIDRASLSTWHVLYSPNVSVPSSSSTPLLYPSLALPAPHLTLPFNWTHCFDPQIQAIVSSPCHNSLILPP
**HAM9**	ILEPSIPWKSKLLTLVQLTLQSTNYTCIVCIDRASLSTWHVLYSPNVSVPSSSSTPLLYPSLALPAPHLTLPFNWTHCFDPQIQAIVSSPCHNSLILPP
**HAM10**	ILEPSIPWKSKLLTLVQLTLQSTNYTCIVCIDRASLSTWHVLYSPNVSVPSSSSTPLLYPSLALPAPHLTLPFNWTHCFDPQIQAIVSSPCHNSLILPP
**HAM11**	ILEPSIPWKSKLLTLVQLTLQSTNYTCIVCIDRASLSTWHVLYSPNVSVPSSSSTPLLYPSLALPAPHLTLPFNWTHCFDPQIQAIVSSPCHNSLILPP
**HAM12**	ILEPSIPWKSKLLTLVQLTLQSTNYTCIVCIDRASLSTWHVLYSPNVSVPSSSSTPLLYPSLALPAPHLTLPFNWTHCFDPQIQAIVSSPCHNSLILPP

**HAM1**	ILEPSIPWKSKLLTLVQLTLQSTNYTCIVCIDRASLSTWHVLYSPNVSIPSSSSTPLLYPSLALPAPHLTLPFNWTHCFDPQIQAIVSSPCHNSLILPP
**HAM2**	ILEPSIPWKSKLLTLVQLTLQSTNYTCIVCIDRASLSTWHVLYSPNVSVPSSSSTPLLYPSLALPAPHLTLPFNWTHCFDPQIQAIVSSPCHNSLILPP
**HAM3**	ILEPSIPWKSKLLTLVQLTLQSTNYTCIVCIDRASLSTWHVLYSPNVSIPSSSSTPLLYPSLALPAPHLTLPFNWTHCFDPQIQAIVSSPCHNSLILPP
**HAM4**	ILEPSIPWKSKLLTLVQLTLQSTNYTCIVCIDRASLSTWHVLYSPNVSVPSSSSTPLLYPSLALPAPHLTLPFNWTHCFDPQIQAIVSSPCHNSLILPP
**HAM5**	ILEPSIPWKSKLLTLVQLTLQSTNYTCIVCIDRASLSTWHVLYSPNVSVPSSSSTPLLYPSLALPAPHLTLPFNWTHCFDPQIQAIVSSPCHNSLILPP
**HAM6**	ILEPSIPWKSKLLTLVQLTLQSTNYTCIVCIDRASLSTWHVLYSPNVSVPSSSSTPLLYPSLALPAPHLTLPFNWTHCFDPQIQAIVSSPCHNSLILPP
**HAM7**	ILEPSIPWKSKLLTLVQLTLQSTNYTCIVCIDRASLSTWHVLYSPNVSVPSSSSTPLLYPSLALPAPHLTLPFNWTHCFDPQIQAIVSSPCHNSLILPP
**HAM8**	ILEPSIPWKSKLLTLVQLTLQSTNYTCIVCIDRASLSTWHVLYSPNVSVPSSSSTPLLYPSLALPAPHLTLPFNWTHCFDPQIQAIVSSPCHNSLILPP
**HAM9**	ILEPSIPWKSKLLTLVQLTLQSTNYTCIVCIDRASLSTWHVLYSPNVSVPSSSSTPLLYPSLALPAPHLTLPFNWTHCFDPQIQAIVSSPCHNSLILPP
**HAM10**	ILEPSIPWKSKLLTLVQLTLQSTNYTCIVCIDRASLSTWHVLYSPNVSVPSSSSTPLLYPSLALPAPHLTLPFNWTHCFDPQIQAIVSSPCHNSLILPP

**ACH**	ILEPSIPWKSKLLTLVQLTLQSTNYTCIVCIDRASLSTWHVLYSPNVSVPSSSSTPLLYPSLALPAPHLTLPFNWTHCFDPQIQAIVSSPCHNSLILPP
**K30p**	ILEPSIPWKSKLLTLVQLTLQSTNYTCIVCIDRASLSTWHVLYSPNVSVPSSSSTPLLYPSLALPAPHLTLPFNWTHCFDPQIQAIVSSPCHNSLILPP

	FSLSPVPTLGSRSRRAVPVAVWLVSALAMGAGVAGGITGSMSLASGKSLLHEVDKDISQLTQAIVKNHKNLLKIAQYAAQNRRGLDLLFWEQGGLCKAL

**HAM1**	FSLSPVPTLGSRSRRAVPVAVWLVSALAMGAGVAGGITGSMSLASGKSLLHEVDKDISQLTQAIVKNHKNLLKIAQYAAQNRRGLDLLFWEQGGLCKAL
**HAM2**	FSLSPVPTLGSRSRRAVPVAVWLVSALAMGAGVAGGITGSMSLASGKSLLHEVDKDISQLTQAIVKNHKNLLKIAQYAAQNRRGLDLLFWEQGGLCKAL
**HAM3**	FSLSPVPTLGSRSRRAVPVAVWLVSALAMGAGVAGGITGSMSLASGKSLLHEVDKDISQLTQAIVKNHKNLLKIAQYAAQNRRGLDLLFWEQGGLCKAL
**HAM4**	FSLSPVPTLGSRSRRAVPVAVWLVSALAMGAGVAGGITGSMSLASGKSLLHEVDKDISQLTQAIVKNHKNLLKIAQYAAQNRRGLDLLFWEQGGLCKAL
**HAM5**	FSLSPVPTLGSRSRRAVPVAVWLVSALAMGAGVAGGITGSMSLASGKSLLHEVDKDISQLTQAIVKNHKNLLKIAQYAAQNRRGLDLLFWEQGGLCKAL
**HAM6**	FSLSPVPTLGSRSRRAVPVAVWLVSALAMGAGVAGGITGSMSLASGKSLLHEVDKDISQLTQAIVKNHKNLLKIAQYAAQNRRGLDLLFWEQGGLCKAL
**HAM7**	FSLSPVPTLGSRSRRAVPVAVWLVSALAMGAGVAGGITGSMSLASGKSLLHEVDKDISQLTQAIVKNHKNLLKIAQYAAQNRRGLDLLFWEQGGLCKAL
**HAM8**	FSLSPVPTLGSRSRRAVPVAVWLVSALAMGAGVAGGITGSMSLASGKSLLHEVDKDISQLTQAIVKNHKNLLKIAQYAAQNRRGLDLLFWEQGGLCKAL
**HAM9**	FSLSPVPTLGSRSRRAVPVAVWLVSALAMGAGVAGGITGSMSLASGKSLLHEVDKDISQLTQAIVKNHKNLLKIAQYAAQNRRGLDLLFWEQGGLCKAL
**HAM10**	FSLSPVPTLGSRSRRAVPVAVWLVSALAMGAGVAGGITGSMSLASGKSLLHEVDKDISQLTQAIVKNHKNLLKIAQYAAQNRRGLDLLFWEQGGLCKAL
**HAM11**	FSLSPVPTLGSRSRRAVPVAVWLVSALAMGAGVAGGITGSMSLASGKSLLHEVDKDISQLTQAIVKNHKNLLKIAQYAAQNRRGLDLLFWEQGGLCKAL
**HAM12**	FSLSPVPTLGSRSRRAVPVAVWLVSALAMGAGVAGGITGSMSLASGKSLLHEVDKDISQLTQAIVKNHKNLLKIAQYAAQNRRGLDLLFWEQGGLCKAL

**HAM1**	FSLSPVPTLGSRSRRAVPVAVWLVSALAMGAGVAGGITGSMSLASGKSLLHEVDKDISQLTQAIVKNHKNLLKIAQYAAQNRRGLDLLFWEQGGLCKAL
**HAM2**	FSLSPVPTLGSRSRRAVPVAVWLVSALAMGAGVAGGITGSMSLASGKSLLHEVDKDISQLTQAIVKNHKNLLKIAQYAAQNRRGLDLLFWEQGGLCKAL
**HAM3**	FSLSPVPTLGSRSRRAVPVAVWLVSALAMGAGVAGGITGSMSLASGKSLLHEVDKDISQLTQAIVKNHKNLLKIAQYAAQNRRGLDLLFWEQGGLCKAL
**HAM4**	FSLSPVPTLGSRSRRAVPVAVWLVSALAMGAGVAGGITGSMSLASGKSLLHEVDKDISQLTQAIVKNHKNLLKIAQYAAQNRRGLDLLFWEQGGLCKAL
**HAM5**	FSLSPVPTLGSRSRRAVPVAVWLVSALAMGAGVAGGITGSMSLASGKSLLHEVDKDISQLTQAIVKNHKNLLKIAQYAAQNRRGLDLLFWEQGGLCKAL
**HAM6**	FSLSPVPTLGSRSRRAVPVAVWLVSALAMGAGVAGGITGSMSLASGKSLLHEVDKDISQLTQAIVKNHKNLLKIAQYAAQNRRGLDLLFWEQGGLCKAL
**HAM7**	FSLSPVPTLGSRSRRAVPVAVWLVSALAMGAGVAGGITGSMSLASGKSLLHEVDKDISQLTQAIVKNHKNLLKIAQYAAQNRRGLDLLFWEQGGLCKAL
**HAM8**	FSLSPVPTLGSRSRRAVPVAVWLVSALAMGAGVAGGITGSMSLASGKSLLHEVDKDISQLTQAIVKNHKNLLKIAQYAAQNRRGLDLLFWEQGGLCKAL
**HAM9**	FSLSPVPTLGSRSRRAVPVAVWLVSALAMGAGVAGGITGSMSLASGKSLLHEVDKDISQLTQAIVKNHKNLLKIAQYAAQNRRGLDLLFWEQGGLCKAL
**HAM10**	FSLSPVPTLGSRSRRAVPVAVWLVSALAMGAGVAGGITGSMSLASGKSLLHEVDKDISQLTQAIVKNHKNLLKIAQYAAQNRRGLDLLFWEQGGLCKAL

**ACH**	FSLSPVPTLGSRSRRAVPVAVWLVSALAMGAGVAGGITGSMSLASGKSLLHEVDKDISQLTQAIVKNHKNLLKIAQYAAQNRRGLDLLFWEQGGLCKAL
**K30p**	FSLSPVPTLGSRSRRAVPVAVWLVSALAMGAGVAGGITGSMSLASGKSLLHEVDKDISQLTQAIVKNHKNLLKIAQYAAQNRRGLDLLFWEQGGLCKAL

QEQCCFLNITNSHVSILQERPPLENRVLTG*GLNWDLGLSQWAREALQTGITLVALLLLVILAGPCILRQLRHLPSRVRYPHYSLINPESSL

**HAM1**	QEQCCFLNITNSHVSILQERPPLENRVLTGWGLNWDLGLSQWAREALQTGITLVALLLLVILAGPCILRQLRHLPSRVRYPHYSLINPESSL
**HAM2**	QEQCCFLNITNSHVSILQERPPLENRVLTGWGLNWDLGLSQWAREALQTGITLVALLLLVILAGPCILRQLRHLPSRVRYPHYSLINPESSL
**HAM3**	QEQCCFLNITNSHVSILQERPPLENRVLTGWGLNWDLGLSQWAREALQTGITLVALLLLVILAGPCILRQLRHLPSRVRYPHYSLINPESSL
**HAM4**	QEQCCFLNITNSHVSILQERPPLENRVLTGWGLNWDLGLSQWAREALQTGITLVALLLLVILAGPCILRQLRHLPSRVRYPHYSLINPESSL
**HAM5**	QEQCCFLNITNSHVSILQERPPLENRVLTGWGLNWDLGLSQWAREALQTGITLVALLLLVILAGPCILRQLRHLPSRVRYPHYSLINPESSL
**HAM6**	QEQCCFLNITNSHVSILQERPPLENRVLTGWGLNWDLGLSQWAREALQTGITLVALLLLVILAGPCILRQLRHLPSRVRYPHYSLINPESSL
**HAM7**	QEQCCFLNITNSHVSILQERPPLENRVLTGWGLNWDLGLSQWAREALQTGITLVALLLLVILAGPCILRQLRHLPSRVRYPHYSLINPESSL
**HAM8**	QEQCCFLNITNSHVSILQERPPLENRVLTGWGLNWDLGLSQWAREALQTGITLVALLLLVILAGPCILRRLRHLPSRVRYPHYSLINPESSL
**HAM9**	QEQCCFLNITNSHVSILQERPPLENRVLTGWGLNWDLGLSQWAREALQTGITLVALLLLVILAGPCILRQLRHLPSRVRYPHYSLINPESSL
**HAM10**	QEQCCFLNITNSHVSILQERPPLENRVLTGWGLNWDLGLSQWAREALQTGITLVALLLLVILAGPCILRQLRHLPSRVRYPHYSLINPESSL
**HAM11**	QEQCCFLNITNSHVSILQERPPLENRVLTGWGLNWDLGLSQWAREALQTGITLVALLLLVILAGPCILRQLRHLPSRVRYPHYSLINPESSL
**HAM12**	QEQCCFLNITNSHVSILQERPPLENRVLTGWGLNWDLGLSQWAREALQTGITLVALLLLVILAGPCILRQLRHLPSRVRYPHYSLINPESSL

**HAM1**	QEQCCFLNITNSHVSILQERPPLENRVLTGWGLNWDLGLSQWAREALQTGITLVALLLLVILAGPCILRQLRHLPSRVRHPHYSLINPESSL
**HAM2**	QEQCCFLNITNSHVSILQERPPLENRVLTGWGLNWDLGLSQWAREALQTGITLVALLLLVILAGPCILRQLRHLPSRVRHPHYSLINPESSL
**HAM3**	QEQCCFLNITNSHVSILQERPPLENRVLTGWGLNWDLGLSQWAREALQTGITLVALLLLVILAGPCILRQLRHLPSRVRHPHYSLINPESSL
**HAM4**	QEQCCFLNITNSHVSILQERPPLENRVLTGWGLNWDLGLSQWAREALQTGITLVALLLLVILAGPCILRQLRHLPSRVRHPHYSLINPESSL
**HAM5**	QEQCCFLNITNSHVSILQERPPLENRVLTGWGLNWDLGLSQWAREALQTGITLVALLLLVILAGPCILRQLRHLPSRVRHPHYSLINPESSL
**HAM6**	QEQCCFLNITNSHVSILQERPPLENRVLTGWGLNWDLGLSQWAREALQTGITLVALLLLVILAGPCILRQLRHLPSRVRHPHYSLINPESSL
**HAM7**	QEQCCFLNITNSHVSILQERPPLENRVLTGWGLNWDLGLSQWAREALQTGITLVALLLLVILAGPCILRQLRHLPSRVRHPHYSLINPESSL
**HAM8**	QEQCCFLNITNSHVSILQERPPLENRVLTGWGLNWDLGLSQWAREALQTGITLVALLLLVILAGPCILRQLRHLPSRVRHPHYSLINPESSL
**HAM9**	QEQCCFLNITNSHVSILQERPPLENRVLTGWGLNWDLGLSQWAREALQTGITLVALLLLVILAGPCILRQLRHLPSRVRHPHYSLINPESSL
**HAM10**	QEQCCFLNITNSHVSILQERPPLENRVLTGWGLNWDLGLSQWAREALQTGITLVALLLLVILAGPCILRQLRHLPSRVRHPHYSLINPESSL

**ACH**	QEQCCFLNITNSHVSILQERPPLENRVLTGWGLNWDLGLSQWAREALQTGITLVALLLLVILAGPCILRQLRHLPSRVRYPHYSLINPESSL
**K30p**	QEQCCFLNITNSHVSILQERPPLENRVLTGWGLNWDLGLSQWAREALQTGITLVALLLLVILAGPCILRQLRHLPSRVRYPHYSLINPESSL
