# Supplementary material for: Characterization of HTLV-1 Infectious Molecular Clone Isolated from Patient with HAM/TSP and Immortalization of Human Primary T-Cell Lines
Source: Viruses. 2024 Nov 9;16(11):1755. doi: 10.3390/v16111755 (PMC11599126; doi:10.3390/v16111755)
Supplement: Supplementary file 1 [file viruses-16-01755-s001.zip › Supplemental S8 P12 align.pdf]

HTLV-1 P12 ALIGNMENTS BETWEEN JAPANESE AND BRAZILIAN HAM/TSP ISOLATES (FULL GENOME SEQUENCE) AND OTHER HTLV-1 MOLECULAR CLONES

**PBST**	MLFRLLSPLSPLALTALLLFLLSPGDVSSLLLRLPPAPCLLLFLPFQILSNLLFLLFLPLFFSLPLLLSPSLPITMRFPARWRFLPWRAPSQPAAAFLF

**HAM1**	MLFRLLSPLSPLALTALLLFLLSPGDVSGLLLRPPPAPCLLLFLPFQILSNLLFLLFLPLFFSLPLLLSPSLPITMRFPARWRFLPWRALSQPAAAFLF
**HAM2**	MLFRLLSPLSPLALTALLLFLLSPGDVSGLLLRPPPAPCLLLFLPFQILSNLLFLLFLPLFFSLPLLLSPSLPITMRFPARWRFLPWRAPSQPAAAFLF
**HAM3**	MLFRLLSPLSPLALTALLLFLLSPGDVSGLLLRPPPAPCLLLFLPFQILSNLLFLLFLPLFFLLPPLLSPSLPITMRFPARWRFLPWRAPSQPAAAFLF
**HAM4**	MLFRLLSPLSPLALTALLLFLLSPGDVSGLLLRPPPAPCLLLFLPFQILSNLLFLLFLPLFFLLPLLLSPSLPITMRFPARWRFLPWRAPSQPAAAFLF
**HAM5**	MLFRLLSPLSPLALTALLLFLLSPGDVSGLLLRPPPAPCLLLFLPFQILSNLLFLLFLPLFFSLPLLLSPSLPITMRFPARWRFLPWRAPSQPAAAFLF
**HAM6**	MLFRLLSPLSPLALTALLLFLLSPGDVSGLLLRPPPAPCLLLFLPFQILSNLLFLLFLPLFFSLPLLLSPSLPITMRFPARWRFLPWRAPSQPAAAFLF
**HAM7**	MLFRLLSPLSPLALTALLLFLLSPGDVSGLLLRPPPAPCLLLFLPFQILSNLLFLLFLPLFFSLPLLLSPSLPITMRFPARWRFLPWRAPSQPAAAFLF
**HAM8**	MLFRLLSPLSPLALTALLLFLLSPGDVSGLLLRPPPAPCLLFFLPFQILSNLLFLLFLPLFFPLPLLLSPSLPITMRFPARWRFLPWRAPPQSAAAFLF
**HAM9**	MLFRLLSPLSPLALTALLLFLLSPGDVSGLLLRPPPAPCLLLFLPFQILSNLLFLLFLPLFFSLPLLLSPSLPITMRFPARWRFLPWRAPSQPAAAFLF
**HAM10**	MLFRLLSPLSPLALTALLLFLLSPGDVSGLLLRPPPAPCLLLFLPFQILSNLLFLLFLPLFFSLPLLLSPSLPLTMRFPARWRFLPWRAPSQPAAAFLF
**HAM11**	MLFRLLSPLSPLALTALLLFLLSPGDVSGPLLRPPPAPCLLLFLPFQILSNLLFLLFLPLFFSLPLLLSPSLPITMRFPARWRFLPWRAPSQPAAAFLF
**HAM12**	MLFRLLSPLSPLALTALLLFLLSPGDVSGLLLRPPPAPCLLLFLPFQILSNLLFLLFLPLFFSLPLLLSPSLPITMRFPARWRFLPWRALSQPAAAFLF

**HAM1**	MLFRLLSPLSPLALTALLLFLLSPGDVSGLLLRLPPAPCLLLFLPFQILSNLLFLLFLPLFFPLPLLLSPSLPITMRFPARWRFLPWRAPPQPAAAFLF
**HAM2**	MLFRLLSPLSPLALTALLLFLLSPSDVSSLLLRLPPAPCLLLFLPFQILSNLLLLLFLPLFFPLPLLLSPSLPITMRFPARWRFLPWRAPPQPAAAFLF
**HAM3**	MLFRLLSPLSPLALTALLLFLLSPGDVSGLLLRPPPAPCLLLFLPFQILSNLLFLLFLPLFFPLPLLLSPSLPITMRFPARWRFLPWRTPPQPAAAFLF
**HAM4**	MLFRLLSPLSPLALTALLLFLLSPGDVSSLLLRLPPAPCLLLFLPFQILSNLLFLLFLPLFLSLPLLLSPSLPITMRFPARWRFLPWRAPSQPAAAFLF
**HAM5**	MLFRLLSPLSPLALTALLLFLLSPGDVSGLLLRLPPAPCLLLFLPFQILSNLLFLLFLPLFFPLPLLLSPSLPITMRFPARWRFLPWRAPPQPAAAFLF
**HAM6**	MLFRLLSPLSPLALTALLLFLLSPGDVSGLLLRLPPAPCLLLFLPFQILSNLLFLLFLPLFFPLPLLLSPSLPITMRFPARWRFLPWRAPPQPAAAFLF
**HAM7**	MLFRLLSPLSPLALTALLLFLLSPGDVSGLLLRPPPAPCLLLFLPFQILSNLLFLLFLPLFFPLPLLLSPSLPITMRFPARWRFLPWRAPPQPAAAFLF
**HAM8**	MLFRLLSPLSPLALTALLLFLLSPGDVSGLLLRPPPAPCLLLFLPFQILSNLLFLLFLPLFFPLPLLLSPSLPITMRFPARWRFLPWRAPPQPAAAFLF
**HAM9**	MLFRLLSPLSPLALTALLLFLLSPGDVSSLLLRLPPAPCLLLFLPFQILSNLLFLLFLPLFFPLPLLLSPSLPITMRFPARWRFLPWRAPPQPAAAFLF
**HAM10**	MLFRLLSPLSPLALTALLLFLLSPGDVSGLLLRLPPAPCLLLFLPFQILSNLLFLLFLPLFFPLPLLLSPSLPITMRFPARWRFLPWRAPPQPAAAFLF

**ACH**	MLFRLLSPLSPLALTALLLFLLSPGDVSSLLLRLPPAPCLLLFLPFQILSNLLFLLFLPLFFSLPLLLSPSLPITMRFPARWRFLPWRAPSQPAAAFLF
**K30p**	MLFRLLSPLSPLALTALLLFLLSPGNVSGLLLRLPPAPCLLLFLPFQILSNLLFLLFLPLFFSLPLLLSPSLPITMRFPARWRFFPWRAPSQPAAAFLF
