# Supplementary material for: Characterization of HTLV-1 Infectious Molecular Clone Isolated from Patient with HAM/TSP and Immortalization of Human Primary T-Cell Lines
Source: Viruses. 2024 Nov 9;16(11):1755. doi: 10.3390/v16111755 (PMC11599126; doi:10.3390/v16111755)
Supplement: Supplementary file 1 [file viruses-16-01755-s001.zip › Supplemental S9 P30 align.pdf]

HTLV-1 P30 ALIGNMENTS BETWEEN JAPANESE AND BRAZILIAN HAM/TSP ISOLATES (FULL GENOME SEQUENCE) AND OTHER HTLV-1 MOLECULAR CLONES

**PBST**	MALCCFAFSAPCLHLRSRRSCSSCFLRATSAAFFSACLLRRAFSSSFLFKYSAICFSSSFSRSFFRFLFSSARRCRSRCVSPRGGAFSPGGPRRSRPRLSSSK

**HAM1**	MALCCFAFSAPCLHLRSRRSCSSCFLRATSAAFFSARLLRRAFSSSFLFKYSAICFSSSFSRSFFRFLFSSARRCRSRCVSPRGGAFSPGGPCRSRPRLSSSK
**HAM2**	MALCCFAFSAPCLHLRSRRSCSSCFLRATSAAFFSARLLRRAFSSSFLFKYSAICFSSSFSRSFFRFLFSSARRCRSRCVSPRGGAFSPGGPRRSRPRLSSSK
**HAM3**	MALCCFAFSAPCLHLRSRRSCSSCFLRATSAAFFSARLLRRAFSSSFLFKYSAICFSSSFSRSFFCFLLSSARRCRSRCVSPRGGAFSPGGPRRSRPRLSSSK
**HAM4**	MALCCFAFSAPCLHLRSRRSCSSCFLRATSAAFFSARLLRCAFSSSFLFKYSAICFSSSFSRSFFCFLFSSARRCRSRCVSPRGGAFSPGGPRRSRPRLSSSK
**HAM5**	MALCCFAFSAPCLHLRSRRSCSSCFLRATSAAFFSARLLRRAFSSSFLFKYSAICFSSSFSRSFFRFLFSSARRCRSRCVSPRGGAFSPGGPRRSRPQLSSSK
**HAM6**	MALCCFAFSAPCLHLRSRRSCSSCFLRATSAAFFSARLLRRAFSSSFLFKYSAICFSSSFSRSFFRFLFSSARRCRSRCVSPRGGAFSPGGPRRSRPRLSSSK
**HAM7**	MALCCFAFSAPCLHLRSRRSCSSCFLRATSAAFFSARLLRRAFSSSFLFKYSAICFSSSFSRSFFRFLFSSARRCRSRCVSPRGGAFSPGGPRRSRPRLSSSK
**HAM8**	MALCCFAFSAPCLHLRSRRSCSSCFLRATSAAFFSARLLRRAFSSSFLFKYSAICFSSSFSRSFFRFLFSSARRCRSRCVSPRGGAFSPGGPRRSRPRLSSSK
**HAM9**	MALCCFAFSAPCLHLRSRRSCSSCFLRATSAAFFSARLLRRAFSSSFLFKYSAICFSSSFSRSFFRFLFSSARRCRSRCVSPRGGAFSPGGPRRSRPRLSSSK
**HAM10**	MALCCFAFSAPCLHLRSRRSCSSCFLRATSAAFFSARLLRRAFSSSFLFKYSAICFSSSFSRSFFRFLFSSARRCRSRCVSPRGGAFSPGGPRRSRPRLSSSK
**HAM11**	MALCCFAFSAPCLHLRSRRSCSSCFLRATSAALFSARLLRRAFSSSFLFKYSAICFSSSFSRSFFRFLFSSARRCRSRCVSPRGGAFSPGGPRRSRPRLSSSK
**HAM12**	MALCCFAFSAPCLHLRSRRSCSSCFLRATSAAFFSARLLRRAFSSSFLFKYSAICFSSSFSRSFFRFLFSSARRCRSRCVSPRGGAFSPGGPRRSRPRLSSSK

**HAM1**	MALCCFAFSAPCLHLRSRRSCSSCFLRATSAAFFSARLLRRAFSSSFLFKYSAICFSSSFSRSFFRFLFSSARRCRSRCVSPRGGAFSPGGPRRSRPRLSSSK
**HAM2**	MALCCFAFSAPCLHLRSRRSCSSCFLRATSAAFFSACLLRRAFSSSFLFKYSAICFSSSFSRSFFRFLFSSARRCRSRCVSPRGGAFSPGGPRRSRPRLSSSK
**HAM3**	MALCCFAFSAPCLHLRSRRSCSSCFLRATSAAFFSACLLRRAFSSSFLFKYSAICFSSSFSRSFFRFLFSSARRCRSRCVSPRGGAFSPGGPRRSRPRLSSSK
**HAM4**	MALCCFAFSAPCLHLRSRRSCSSCFLRATSAAFFSACLLRRAFSSSFLFKYSAICFSSSFSRSFFRFLFSSARRCRSRCVSPRGGAFSPGGPRRSRPRLSSSK
**HAM5**	MALCCFAFSAPCLHLRSRRSCSSCFLRATSAAFFSACLLRRAFSSSFLFKYSAICFSSSFSRSFFRFLFSSARRCRSRCVSPRGGAFSPGGPRRSRPRLSSSK
**HAM6**	MALCCFAFSAPCLHLRSRRSCSSCFLRATSAAFFSARLLRRAFSSSFLFKYSAICFSSSFSRSFFRFLFSSARRCRSRCVSPRGGAFSPGGPRRSRPRLSSSK
**HAM7**	MALCCFAFSAPCLHLRSRRSCSSCFLRATSAAFFSARLLRRAFSSSFLFKYSAICFSSSFSRSFFRFLFSSARRCRSRCVSPRGGAFSPGGPRRSRPRLSSSK
**HAM8**	MALCCFAFSAPCLHLRSRRSCSSCFLRATSAAFFSARLLRRAFSSSFLFKYSAICFSSSFSRSFFRFLFSSARRCRSRCVSPRGGAFSPGGPRRSRPRLSSSK
**HAM9**	MALCCFAFSAPCLHLRSRRSCSSCFLRATSAAFFSARLLRRAFSSSFLFKYSAICFSSSFSRSFFRFLFSSARRCRSRCVSPRGGAFSPGGPRRSRPRLSSSK
**HAM10**	MALCCFAFSAPCLHLRSRRSCSSCFLRATSAAFFSACLLRRAFSSSFLFKYSAICFSSSFSRSFFRFLFSSARRCRSRCVSPRGGAFSPGGPRRSRPRLSSSK

**ACH**	MALCCFAFSAPCLHLRSRRSCSSCFLRATSAAFFSACLLRRAFSSSFLFKYSAICFSSSFSRSFFRFLFSSARRCRSRCVSPRGGAFSPGGPRRSRPRLSSSK
**K30p**	MALCCFAFSAPCLHLRSRRSCSSCFLRATSAAFFSARLLRRAFSSSFLFKYSAICFSSSFSRSFFRFLFSSARRCRSRCVSPRGGAFSPGGPRRSRPRLSSSK

	DSKPSSTASSSSLSFNSSSKDNSPSTNSSTSRSSGHGTGKHRNSPTDTKLTMLIISPLPRVWTESSFRIPSLRV*RLCTRRLVPHLWGTMFGPPTSSRPTGHL

**HAM1**	DSKPSSTASSSSLSFNSSSKDNSPSTNSSTSRSSGHGPGKHRNSPTDTKLTMLIISPLPRVWTESSFRIPSLRVWRLCTRRLVPHLWGTMFGPPTSSRPTGHL
**HAM2**	DSKPSSTASSSSLSFNSSSKDNSPSTNSSTSRSSGHGTGKHRNGPTDTKLTMLIISPLPRVWTESSFRIPSLRVWRLCTRRLVPHLWGTMFGPPTSSRPTGHL
**HAM3**	DSKPSSTASSSSLSFNSSSKDNSPSTNSSTSRSSGHGTGKHRNSPTDTKLTMLIISPLPRVWTESSFRIPSLRVWRLCTRRLVPHLWGTMFGPPTSSRPTGHL
**HAM4**	DSKPSSTASSSSLSFNSSSKDNSPSTNSSTSRSSGHGTGKHRNSPTDTKLTMLIISPLPRVWTESSFRIPSLRVWRLCTRRLVPHLWGTMFGPPTSSRPTGHL
**HAM5**	DSKPSSTASSSSLSFNSSSKDNSPSTNSSTSRSSGHGTGKHRNSPTDTKLTMLIISPLPRVWTESSFRIPSLRVWRLCTRRLVPHLWGTMFGPPTSSRPTGHL
**HAM6**	DSKPSSTASSSSLSFNSSSKDNSPSTNSSTSRSSGHGAGKHRNSPTDTKLTMLIISPLPRVWTESSFRIPSLRVWRLCTRRLVPHLWGTMFGPPTSSRPTGHL
**HAM7**	DSKPSSTASSSSLSFNSSSKDNSPSTNSSTSRSSGHGTGKHRNSPTDTKLTMLIISPLPRVWTESSFRIPSLRVWRLCTRRLVPHLWGTMFGPPTSSRPTGHL
**HAM8**	DSKPSSTASSSSLSFNSSSKDNSPSTNSSTSRSSGHGTGKHRNSPTDTKLTMLIISPLPRVWTESSFRIPSLRVWRLCTRRLVPHLWGTMFGPPTSSRPTGHL
**HAM9**	DSKPSSTASSSSLSFNSSSKDNSPSTNSSTSRSSGHGIGKHRNSPTDTKLTMLIISPLPRVWTESSFRIPSLRVWRLCTRRLVPHLWGTMFGPPTSSRPTGHL
**HAM10**	DSKPSSTASSSSLSFNSSSKDNSPSTNSSTSRSSGHGTGKHRNSPTDTKLTMLIISPLPRVWTESSFRIPSLRVWRLCTRRLVPHLWGTMFGPPTSSRPTGHL
**HAM11**	DSKPSSTASSSSLSFNSSSKDNSPSTNSSTSRSSGHGTGKHRNSPTDTKLTMLIISPLPRVWTESSFRIPSLRVWRLCTRRLVPHLWGTMFGPPTSSRPTGHL
**HAM12**	DSKPSSTASSSSLSFNSSSKDNSPSTNSSTSRSSGHGIGKHRNSPTDTKLTMLIISPLPRVWTESSFRIPSLRVWRLCTRRLVPHLWGTMFGPPTSSRPTGHL

**HAM1**	DSKPSSTASSSSLSFNSSSKDNSPSTNSSTSRSSGHGTGRHRNSPTDTKLTMLIISPLPRVWTESSFRIPSLRVWRLCTRRLVPHLWGTMFGPPTSSRPTGHL
**HAM2**	DSKPSSTASSSSLSFNSSSKDNSPSTNSSTSRSSGHGTGKHRNSPTDTKLTMLIISPLPRVWTESSFRIPSLRVWRLCTRRLVPHLWGTMFGPPTSSRPTGHL
**HAM3**	DSKPSSTASSSSLSFNSSSKDNSPSTNSSTSRSSGHGTGRHRNSPTDTKLTMLIISPLPRVWTESSFRIPSLRVWRLCTRRLVPHLWGTMFGPPTSSRPTGHL
**HAM4**	DSKPSSTASSSSLSFNSSSKDNSPSTNSSTSRSSGHGIGKHRNSPTDTKLTMLIISPLPRVWTESSFRIPSLRVWRLCTRRLVPHLWGTMFGPPTSSRPTGHL
**HAM5**	DSKPSSTASSSSLSFNSSSKDNSPSTNSSTSRSSGHGTGRHRNSPTDTKLTMLIISPLPRVWTESSFRIPSLRVWRLCTRRLVPHLWGTMFGPPTSSRPTGHL
**HAM6**	DSKPSSTASSSSLSFNSSSKDNSPSTNSSTSRSSGHGTGRHRNSPTDTKLTMLIISPLPRVWTESSFRIPSLRVWRLCTRRLVPHLWGTMFGPPTSSRPTGHL
**HAM7**	DSKPSSTASSSSLSFNSSSKDNSPSTNSSTSRSSGHGTGRHRNSPTDTKLTMLIISPLPRVWTESSFRIPSLRVWRLCTRRLVPHLWGTMFGPPTSSRPTGHL
**HAM8**	DSKPSSTASSSSLSFNSSSKDNSPSTNSSTSRSSGHGTGRHRNSPTDTKLTMLIISPLPRVWTESSFRIPSLRVWRLCTRRLVPHLWGTMFGPPTSSRPTGHL
**HAM9**	DSKPSSTASSSSLSFNSSSKDNSPSTNSSTSRSSGHGTGKHRNSPTDTKLTMLIISPLPRVWTESSFRIPSLRVWRLCTRRLVPHLWGTMFGPPTSSRPTGHL
**HAM10**	DSKPSSTASSSSLSFNSSSKDNSPSTNSSTSRSSGHGTGRHRNSPTDTKLTMLIISPLPRVWTESSFRIPSLRVWRLCTRRLVPHLWGTMFGPPTSSRPTGHL

**ACH**	DSKPSSTASSSSLSFNSSSKDNSPSTNSSTSRSSGHGTGKHRNSPTDTKLTMLIISPLPRVWTESSFRIPSLRVWRLCTRRLVPHLWGTMFGPPTSSRPTGHL
**K30p**	DSKPSSTASSSSLSFNSSSKDNSPSTNSSTSRSSGHGTGKHRNSPTDTKLTMLIISPLPRVWTESSFWIPSLRVWRLCTRRLVPHLWGTMFGPPTSSRPTGHL

SRASDHLGPHRWTRYRLSSTVPYPSTPLLPHPENL

**HAM1**	SRASDHLGPHRWTRYRLSSTVPYPSTPLLPHPENL
**HAM2**	SRASDHLGPHRWTRYRLSSTVPYPSTPLLPHPENL
**HAM3**	SRASDHLGPHRWTRYRLSSTVPYPSTPLLPHPENL
**HAM4**	SRASDHLGPHRWTRYRLSSTVPYPSTPLLPHPENL
**HAM5**	SRASDHLGPHRWTRYRLSSTVPYPSTPLLPHPENL
**HAM6**	SRASDHLGPHRWTRYRLSSTVPYPSTPLLPHPENL
**HAM7**	SRASDHLGPHRWTRYRLSSTVPYPSTPLLPHPENL
**HAM8**	SRASDHLGPHRWTRYRLSSTVPYPSTPLLPHPENL
**HAM9**	SRASDHLGPHRWTRYRLSSTVPYPSTPLLPHPENL
**HAM10**	SRASDHLGPHRWTRYRLSSTVPYPSTPLLPHPENL
**HAM11**	SRASDHLGPHRWTRYRLSSTVPYPSTPLLPHPENL
**HAM12**	SRASDHLGPHRWTRYRLSSTVPYPSTPLLPHPENL

**HAM1**	SRASDHLGPHRWTRYRLSSTVPYPSTPLLPHPENL
**HAM2**	SRASDHLGPHRWTRYRLSSTVPYPSTPLLPHPENL
**HAM3**	SRASDHLGPHRWTRYRLSSTVPYPSTPLLPHPENL
**HAM4**	SRASDHLGPHRWTRYRLSSTVPYPSTPLLPHPENL
**HAM5**	SRASDHLGPHRWTRYRLSSTVPYPSTPLLPHPENL
**HAM6**	SRASDHLGPHRWTRYRLSSTVPYPSTPLLPHPENL
**HAM7**	SRASDHLGPHRWTRYRLSSTVPYPSTPLLPHPENL
**HAM8**	SRASDHLGPHRWTRYRLSSTVPYPSTPLLPHPENL
**HAM9**	SRASDHLGPHRWTRYRLSSTVPYPSTPLLPHPENL
**HAM10**	SRASDHLGPHRWTRYRLSSTVPYPSTPLLPHPENL

**ACH**	SRASDHLGPHRWTRYRLSSTVPYPSTPLLPHPENL
**K30p**	SRASDHLGPHRWTRYRLSSTVPYPSTPLLPHPENL
