# Supplementary material for: Characterization of HTLV-1 Infectious Molecular Clone Isolated from Patient with HAM/TSP and Immortalization of Human Primary T-Cell Lines
Source: Viruses. 2024 Nov 9;16(11):1755. doi: 10.3390/v16111755 (PMC11599126; doi:10.3390/v16111755)
Supplement: Supplementary file 1 [file Supplemental S11 Rex align.pdf › Supplemental S11 Rex align.pdf]

HTLV-1 REX ALIGNMENTS BETWEEN JAPANESE AND BRAZILIAN HAM/TSP ISOLATES (FULL GENOME SEQUENCE) AND OTHER HTLV-1 MOLECULAR CLONES

**PBST**	MPKTRRRPRRSQRKRPPTPWPTSQGLDRVFFSDTQSTCLETVYKATGAPSLGDYVRPAYIVTPYWPPVQSIRSPGTPSMDALSAQLYSSLSLDSPP

**HAM1**	MPKTRRRPRRSQRKRPPTPWPTSQGLDRVFFSDTQSTCLETVYKATGAPSLGDYVRPAYIVTPYWPPVQSIRSPGTPSMDALSAQLYSSLSLDSPP
**HAM2**	MPKTRRRPRRSQRKRPPTPWPTSQGLDRVFFSDTQSTCLETVYKATGAPSLGDYVRPAYIVTPYWPPVQSIRSPGTPSMDALSAQLYSSLSLDSPP
**HAM3**	MPKTRRRPRRSQRKRPPTPWPTSQGLDRVFFSDTQSTCLETVYKATGAPSLGDYVRPAYIVTPYWPPVQSIRSPGTPSMDALSAQLYSSLSLDSPP
**HAM4**	MPKTRRRPRRSQRKRPPTPWPTSQGLDRVFFSDTQSTCLETVYKATGAPSLGDYVRPAYIVTPYWPPVQSIRSPGTPSMDALSAQLYSSLSLDSPP
**HAM5**	MPKTRRRPRRSQRKRPPTPWPTSQGLDRVFFSDTQSTCLETVYKATGAPSLGDYVRPAYIVTPYWPPVQSIRSPGTPSMDALSAQLYSSLSLDSPP
**HAM6**	MPKTRRRPRRSQRKRPPTPWPTSQGLDRVFFSDTQSTCLETVYKATGAPSLGDYVRPAYIVTPYWPPVQSIRSPGTPSMDALSAQLYSSLSLDSPP
**HAM7**	MPKTRRRPRRSQRKRPPTPWPTSQGLDRVFFSDTQSTCLETVYKATGAPSLGDYVRPAYIVTPYWPPVQSIRSPGTPSMDALSAQLYSSLSLDSPP
**HAM8**	MPKTRRRPRRSQRKRPPTPWPTSQGLDRVFFSDTQSTCLETVYKAIGAPSLGDYVRPAYIVTPYWPPVQSIRSPGTPSMDALSAQLYSSLSLDSPP
**HAM9**	MPKTRRRPRRSQRKRPPTPWPTSQGLDRVFFSDTQSTCLETVYKATGAPSLGDYVRPAYIVTPYWPPVQSIRSPGTPSMDALSAQLYSSLSLDSPP
**HAM10**	MPKTRRRPRRSQRKRPPTPWPTSQGLDRVFFSDTQSTCLETVYKATGAPSLGDYVRPAYIVTPYWPPVQSIRSPGTPSMDALSAQLYSSLSLDSPP
**HAM11**	MPKTRRRPRRSQRKRPPTPWPTSQGLDRVFFSDTQSTCLETVYKATGAPSLGDYVRPAYIVTPYWPPVQSIRSPGTPSMDALSAQLYSSLSLDSPP
**HAM12**	MPKTRRRPRRSQRKRPPTPWPTSQGLDRVFFSDTQSTCLETVYKATGAPSLGDYVRPAYIVTPYWPPVQSIRSPGTPSMDALSAQLYSSLSLDSPP

**HAM1**	MPKTRRRPRRSQRKRPPTPWPTSQGLDRVFFSDTQSTCLETVYKATGAPSLGDYVRPAYIVTPYWPPVQSIRSPGTPSMDALSAQLYSSLSLDSPP
**HAM2**	MPKTRRRPRRSQRKRPPTPWPTSQGLDRVFFSDTQSTCLETVYKAIGAPSLGDYVRPAYIVTPYWPPVQSIRSPGTPSMDALSAQLYSSLSLDSPP
**HAM3**	MPKTRRRPRRSQRKRPPTPWPTSQGLDRVFFSDTQSTCLETVYKAIGAPSLGDYVRPAYIVTPYWPPVQSIRSPGTPSMDALSAQLYSSLSLDSPP
**HAM4**	MPKTRRRPRRSQRKRPPTPWPTSQGLDRVFFSDTQSTCLETVYKATGAPSLGDYVRPAYIVTPYWPPVQSIRSPGTPSMDALSAQLYSSLSLDSPP
**HAM5**	MPKTRRRPRRSQRKRPPTPWPTSQGLDRVFFSDTQSTCLETVYKAIGAPSLGDYVRPAYIVTPYWPPVQSIRSPGTPSMDALSAQLYSSLSLDSPP
**HAM6**	MPKTRRRPRRSQRKRPPTPWPTSQGLDRVFFSDTQSTCLETVYKAIGAPSLGDYVRPAYIVTPYWPPVQSIRSPGTPSMDALSAQLYSSLSLDSPP
**HAM7**	MPKTRRRPRRSQRKRPPTPWPTSQGLDRVFFSDTQSTCLETVYKAIGAPSLGDYVRPAYIVTPYWPPVQSIRSPGTPSMDALSAQLYSSLSLDSPP
**HAM8**	MPKTRRRPRRSQRKRPPTPWPTSQGLDRVFFSDTQSTCLETVYKAIGAPSLGDYVRPAYIVTPYWPPVQSIRSPGTPSMDALSAQLYSSLSLDSPP
**HAM9**	MPKTRRRPRRSQRKRPPTPWPTSQGLDRVFFSDTQSTCLETVYKAIGAPSLGDYVRPAYIVTPYWPPVQSIRSPGTPSMDALSAQLYSSLSLDSPP
**HAM10**	MPKTRRRPRRSQRKRPPTPWPTSQGLDRVFFSDTQSTCLETVYKAIGAPSLGDYVRPAYIVTPYWPPVQSIRSPGTPSMDALSAQLYSSLSLDSPP

**ACH**	MPKTRRRPRRSQRKRPPTPWPTSQGLDRVFFSDTQSTCLETVYKATGAPSLGDYVRPAYIVTPYWPPVQSIRSPGTPSMDALSAQLYSSLSLDSPP
**K30p**	MPKTRRRPRRSQRKRPPTPWPTSQGLDRVFFLDTQSTCLETVYKATGAPSLGDYVRPAYIVTPYWPPVQSIRSPGTPSMDALSAQLYSSLSLDSPP

	SPPREPLRPSRSLPRQSLIQPPTFHPPSSRPCANTPPSEMDTWNPPLGSTSQPCLFQTPDSGPKTCTPSGEAPLSACTSTSFPPPSPGPSCPT

**HAM1**	SPPREPLRPSRSLPRQSLIQPPTFHPPSSRPCANTPPSEMDTWNPPLGSTSQPCLFQTPDSGPRTCTPSGEAPLSACTSTSFPPPSPGPSCPT
**HAM2**	SPPREPLRPSRSLPRQSLIQPPTFHPPSSRPCANTPPSEMDTWNPPLGSTSQPCLFQTPDSGPKTCTPSGEAPLFACTSTSFPPPSPGPSCPT
**HAM3**	SPPREPLRPSRSLPRQSLIQPPTFHPPSSRPCANTPPSEMDTWNPPLGSTSQPCLFQTPDSGPKTCTPSGEAPLSACTSTSFPPPSPGPSCPT
**HAM4**	SPPREPLRPSRSLPRQSLIQPPTFHPPSSRPCANTPPSEMDTWNPPLGSTSQPCLFQTPDSGPRTCTPSGEAPLSACTSTSFPPPSPGPSCPT
**HAM5**	SPPREPLRPSRSLPRQSLIQPPTFHPPSSRPCANTPPSEMDTWNPPLGSTSQPCLFQTPDSGPKTCTPSGEAPLSACTSTSFPPPSPGPSCPT
**HAM6**	SPPREPLRPSRSLPRQSLIQPPTFHPPSSRPCANTPPSEMDTWNPPLGSTSQPCLFQTPDSGPKTCTPSGEAPLSACTSTSFPPPSPGPSCPT
**HAM7**	SPPREPLRPSRSLPRQSLIQPPTFHPPSSRPCANTPPSEMDTWNPPLGSTSQPCLFQTPDSGPRTCTPSGEAPLSACTSTSFPPPSPGPSCPT
**HAM8**	SPPREPLRPSRSLPRQSLIQPPTFHPPSSRPCANTPPSEMDTWNPPLGSTSQPCLFQTPDSGPKTCTPSGEAPLSACTSTSFPPPSPGPSCPT
**HAM9**	SPPREPLRPSRSLPRQSLIQPPTFHPPSSRPCANTPPSEMDTWNLPLGSTSQPCLFQTPDSGPKTCTPSGEAPLSACTSTSFPPPSPGPSCPT
**HAM10**	SPPREPLRPSRSLPRQSLIQPPTFHPPSSRPCANTPPSEMDTWNPPLGSTSQPCLFQTPDSGPKTCTPSGEAPLSACTSTSFPPPSPGPSCPT
**HAM11**	SPPREPLRPSRSLPRQSLIQPPTFHPPSSRPCANTPPSEMDTWNPPLGSTSQPCLFQTPDSGPKTCTPSGEAPLSACTSTSFPPPSPGPSCPT
**HAM12**	SPPREPLRPSRSLPRQSLIQPPTFHPPSSRPCANTPPSEMDTWNPPLGSTSQPCLFQTPDSGPKTCTPSGEAPLSACTSTSFPPPSPGPSCPT

**HAM1**	SPPREPLRPSRSLPRQSLIQPPTFHPPSSRPCANTPPSEMDTWNPPLGSTSQPCLFQTPDSGPKTCTPSGEAPLSACTSTSFPPPSPGPSCPT
**HAM2**	SPPREPLRPSRSLPRQSLIQPPTFHPPSSRPCANTPPSEMDTWNPPLGSTSQPCLFQTPDSGPKTCTPSGEAPLSACTSTSFPPPSPGPSCPT
**HAM2**	SPPREPLRPSRSLPRQSLIQPPTFHPPSSRPCANTPPSEMDTWNPPLGSTSQPCLFQTPDSGPKTCTPSGEAPLSACTSTSFPPPSPGPSCPT
**HAM4**	SPPREPLRPSRSLPRQSLIQPPTFHPPSSRPCANTPPSEMDTWNPPLGSTSQPCLFQTPDSGPRTCTPSGEAPLSACTSTSFPPPSPGPSCPT
**HAM5**	SPPREPLRPSRSLPRQSLIQPPTFHPPSSRPCANTPPSEMDTWNPPLGSTSQPCLFQTPDSGPKTCTPSGEAPLSACTSTSFPPPSPGPSCPT
**HAM6**	SPPREPLRPSRSLPRQSLIQPPTFHPPSSRPCANTPPSEMDTWNPPLGSTSQPCLFQTPDSGPKTCTPSGEAPLSACTSTSFPPPSPGPSCPT
**HAM7**	SPPREPLRPSRSLPRQSLIQPPTFHPPSSRPCANTPPSEMDTWNPPLGSTSQPCLFQTPDSGPKTCTPSGEAPLSACTSTSFPPPSPGPSCPT
**HAM8**	SPPREPLRPSRSLPRQSLIQPPTFHPPSSRPCANTPPSEMDTWNPPLGSTSQPCLFQTPDSGPKTCTPSGEAPLSACTSTSFPPPSPGPSCPT
**HAM9**	SPPREPLRPSRSLPRQSLIQPPTFHPPSSRPCANTPPSEMDTWNPLLGSTSQPCLFQTPDSGPKTCTPSGEAPLSACTSTSFPPPSPGPSCPT
**HAM10**	SPPREPLRPSRSLPRQSLIQPPTFHPPSSRPCANTPPSEMDTWNPPLGSTSQPCLFQTPDSGPKTYTPSGEAPLSACTSTSFPPPSPGPSCPT

**ACH**	SPPREPLRPSRSLPRQSLIQPPTFHPPSSRPCANTPPSEMDTWNPPLGSTSQPCLFQTPDSGPKTCTPSGEAPLSACTSTSFPPPSPGPSCPT
**K30p**	SPPREPLRPSRSLPRQSLIQPPTFHPPSSRPCANTPPSEMDTWNPPLGSTSQPCLFQTPDSGPKTCTPSGEAPLSACTSTSFPPPSPGPSCPT
